# Stereo- and Enantioselective Syntheses of (*Z*)-1,3-Butadienyl-2-carbinols via Brønsted Acid Catalysis

**DOI:** 10.1021/acs.orglett.4c04663

**Published:** 2025-01-09

**Authors:** Ming Chen

**Affiliations:** Department of Chemistry, Virginia Tech, Blacksburg, Virginia 24061, United States

## Abstract

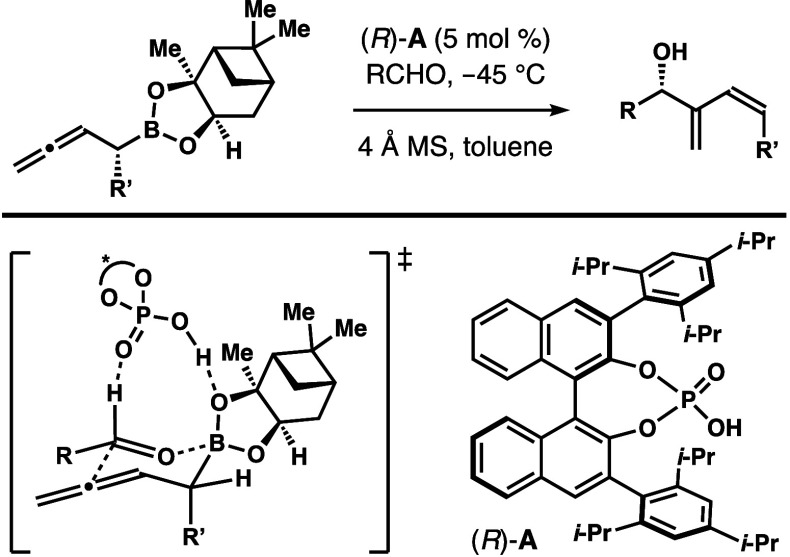

A Brønsted acid-catalyzed enantioselective synthesis of (*Z*)-1,3-butadienyl-2-carbinols is developed. By employing a chiral phosphoric acid as the catalyst, a variety of 1,3-butadienyl-2-carbinols were obtained in good yields with excellent *Z*-selectivities and enantiopurities from α-alkyl-substituted homoallenyl boronates.

The 1,3-butadienyl-2-carbinols with a stereochemically defined 1,2-disubstituted alkene unit and their deoxy-analogs are important building blocks for a variety of natural products that are of great biological importance (Figure 1).^1^ Several methods have been developed for stereoselective syntheses of these structural motifs in the context of complex molecule synthesis.^2,3^ As shown in Scheme 1, the Nicolaou group utilized a sequential olefination approach to access diene **C** in the total synthesis of des-epoxy caribenolide I.^2a^ Horner–Wadsworth–Emmons olefination of an aldehyde with β-ketophosphonate **A** was utilized to generate α,β-unsaturated ketone **B**. Subsequent Wittig olefination to install the methylene group gave diene **C**. In the total synthesis of pteriatoxin,^2b^ Kishi showed that Nozaki–Hiyama–Kishi coupling of vinyl bromide **D** with the aldehyde furnished a diastereomeric mixture of allylic alcohols **E**. Acylation of the hydroxyl group of **E** followed by Pd-mediated elimination afforded diene **C**. An enyne metathesis approach to diene **C** was developed by the Trost group in their synthesis of des-epoxy-amphidinolide N.^2c^ Enyne metathesis of enantioenriched propargylic ether **F** with the alkene substrate using Grubbs’ II catalyst formed an *E*/*Z* mixture of dienes **G**.^2d^ The mixture equilibrated under the reaction conditions over time to form diene **C** with a high *E*-selectivity. The synthesis of racemic (*Z*)-1,3-butadienyl-2-carbinol **K** was reported by Diver and co-workers by employing a multistep reaction sequence.^2d^ Vinyl bromide **H** was converted to allylic alcohol **I** using a Nozaki–Hiyama–Kishi coupling reaction. Alcohol **I** was transformed into aldehyde **J** in four steps, which reacted under the Wittig olefination conditions to give racemic diene **K**. With our research focus in organoboron chemistry,^4^ we became interested in whether asymmetric aldehyde addition with homoallenyl boronate could generate enantioenriched 1,3-butadienyl-2-carbinols (bottom panel, Scheme 1).^5^ Accordingly, we have developed and describe herein chiral phosphoric-acid-catalyzed enantioselective syntheses of *Z*-1,3-butadienyl-2-carbinols **2** from homoallenyl boronate **1**.

**Figure 1:**
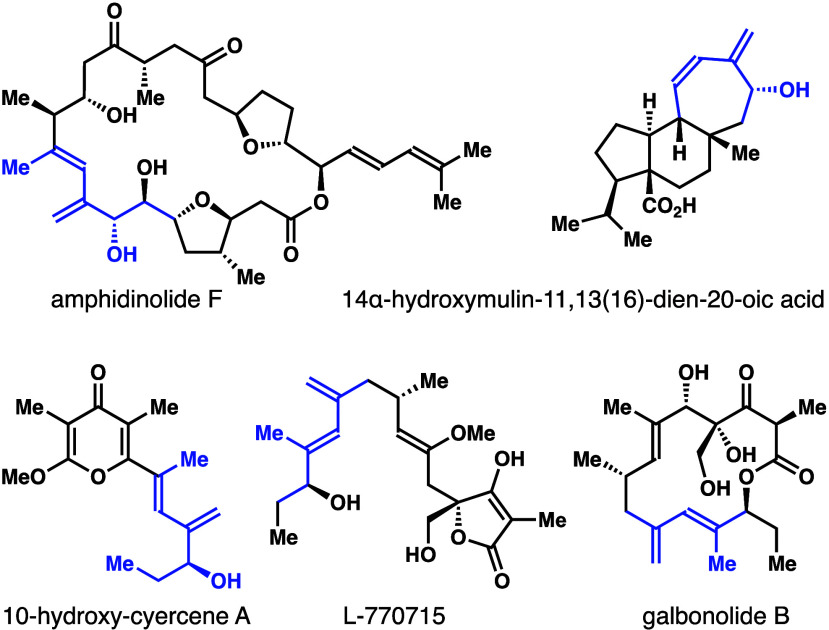
Selected natural products containing the 1,3-butadienyl-2-carbinol motif or the deoxy-analog.

**Scheme 1:**
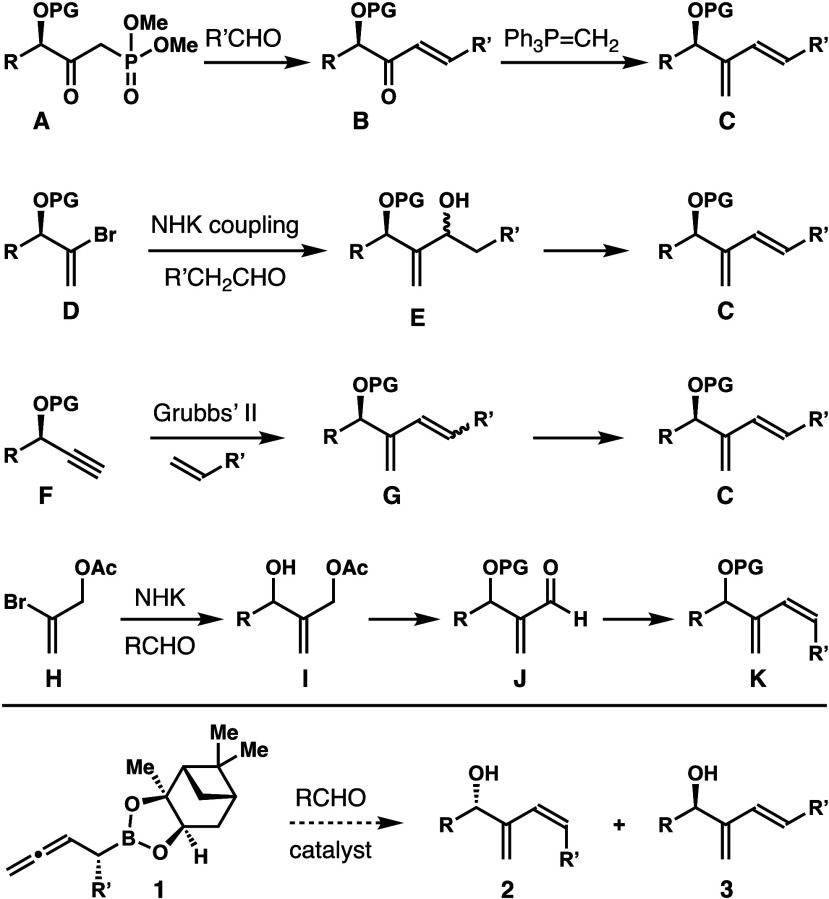
Approaches to 1,3-Butadienyl-2-carbinols

As shown in Scheme 2, homoallenyl boronates **1** were synthesized from pinanediol boronic esters **5** using the conditions developed by Matteson and co-workers.^6^ Treatment of boronic ester **5** at −100 °C with lithiated dichloromethane followed by the addition of a solution of ZnCl_2_ gave an α-chloroboronate intermediate. Subsequent addition of allenyl Grignard reagents to the α-chloroboronate intermediate generated enantioenriched homoallenyl boronates **1** in 72–89% yield.

**Scheme 2:**
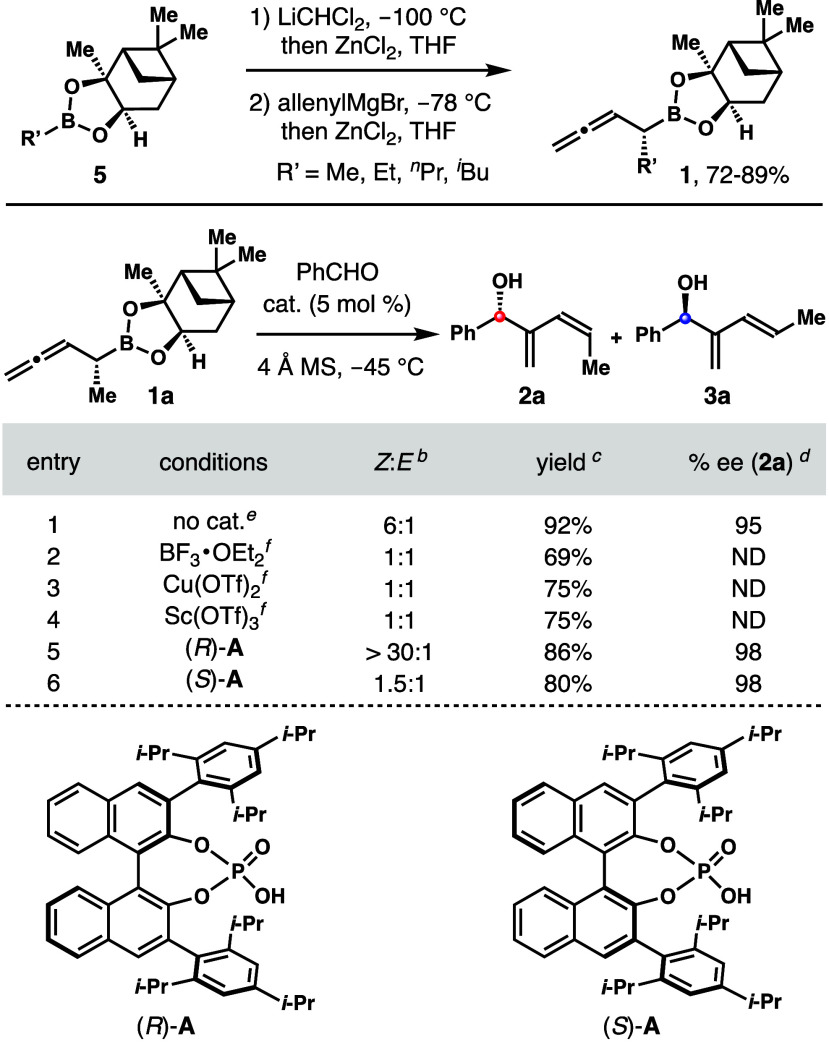
Syntheses of Boron Reagents **1** and Evaluation of the Conditions for Reactions with Boronate **1a** Reaction conditions: boronate **1a** (0.12 mmol, 1.2 equiv), benzaldehyde (0.1 mmol, 1.0 equiv), catalyst (5 mol %), 4 Å molecular sieves (50 mg), toluene (0.3 mL), −45 °C. The *Z*/*E* ratios were determined by ^1^H NMR analyses of the crude reaction products. Yields of isolated products are listed. The enantiomeric excesses were determined by modified Mosher ester analyses. The reaction was conducted at rt. The reaction was conducted in CH_2_Cl_2_.

After successful preparation of reagents **1**, we chose boronate **1a** and benzaldehyde as the model system to study the reactions. As shown in Scheme 2, the reaction of **1a** and benzaldehyde in the absence of any catalyst gave a 6:1 mixture of dienols **2a** and **3a** in a 92% combined yield (entry 1). The enantiomeric purity of major isomer **2a** is excellent (95% ee).^7^ The results indicate that without any catalyst, the reaction of reagent **1a** and benzaldehyde has a 6:1 inherent *Z*-selectivity. Next we sought to identify suitable conditions to improve the *Z*-selectivity. It has been shown that addition of a Lewis acid can drastically affect the *E*/*Z* selectivities of aldehyde addition with α-substituted allylboronates.^8,9^ However, the vast majority of prior studies focused on reactions with pinacol boronates. At the outset of our studies, it is not apparent whether these conditions will be applicable to boronates **1** bearing a pinanediol unit that is considerably larger than pinacol. Indeed, the reaction with BF_3_·OEt_2_ as the catalyst, which has been shown to promote highly stereoselective allylation with several α-substituted allylboronates, formed only a 1:1 mixture of **2a** and **3a** in a combined 69% yield (entry 2). Similar selectivities were observed with either Cu(OTf)_2_ or Sc(OTf)_3_ as the catalyst (entries 3 and 4). Brønsted acids, such as chiral phosphoric acids, have been utilized to catalyze aldehyde addition with a variety of unsaturated organoboronates with excellent enantioselectivities.^10−13^ More recently, they have also been shown to affect the *E*/*Z* selectivity in reactions with α-substituted allylboronates.^14^ Because pinanediol is much larger than pinacol, whether these acid catalysts will tolerate the large pinanediol group is the key to controlling the alkene geometry of alcohols **2** and **3**. In the event, 5 mol % acid (*R*)-**A** was used as the catalyst for the reaction of **1a** with benzaldehyde at −45 °C. Gratifyingly, the reaction generated alcohol **2a** as the only product (*Z*:*E* > 30:1). Alcohol **2a** was isolated in 86% yield with 98% ee (entry 5). By contrast, the reaction with enantiomeric acid (*S*)-**A** as the catalyst only formed a 1.5:1 mixture of **2a** and **3a**, slightly favoring *Z*-isomer **2a**. These data indicate that the asymmetric induction from acid catalyst (*R*)-**A** is the same as the inherent selectivity of reagent **1a** in the reaction with benzaldehyde. The reaction of **1a** with acid (*R*)-**A** as the catalyst is a matched case, and therefore, alcohol **2a** was produced with excellent selectivity. On the other hand, the reaction of **1a** with (*S*)-**A** as the catalyst is mismatched, as the asymmetric induction from acid catalyst (*S*)-**A** is the opposite to the inherent selectivity of reagent **1a**. Ultimately, the mismatched reaction led to the formation of a mixture of **2a** and **3a** with poor selectivity.

With suitable conditions identified, we explored the scope of the reaction. As summarized in Scheme 3, in the presence of 5 mol % acid (*R*)-**A**, the reactions worked well with a wide array of aldehydes to generate 1,3-butadienyl-2-carbinols **2** with excellent *Z*-selectivities and enantiopurities. For example, *para*-substituted aromatic aldehydes reacted smoothly with **1a** to afford products **2b**–**f** in 74–92% yields with 96–99% ee. Aromatic aldehydes with a substituent at the *meta*- or *ortho*-position also reacted to form alcohols **2g**–**j** in 74–88% yields with 97–99% ee. The reactions with α,β-unsaturated aldehydes occurred to give products **2k**–**m** in 71–78% yields with 95–99% ee. Aldehydes with a heterocycle, such as benzothiophene or Boc-protected indole, reacted with **1a** to generate alcohols **2n**–**p** in 73–91% yields with 94–98% ee. Moreover, reactions with aliphatic aldehydes also worked well, furnishing products **2q**–**r** in 74–77% yield with 94–99% ee. In all cases, *Z*-isomers were obtained with excellent selectivities (>30:1).

**Scheme 3:**
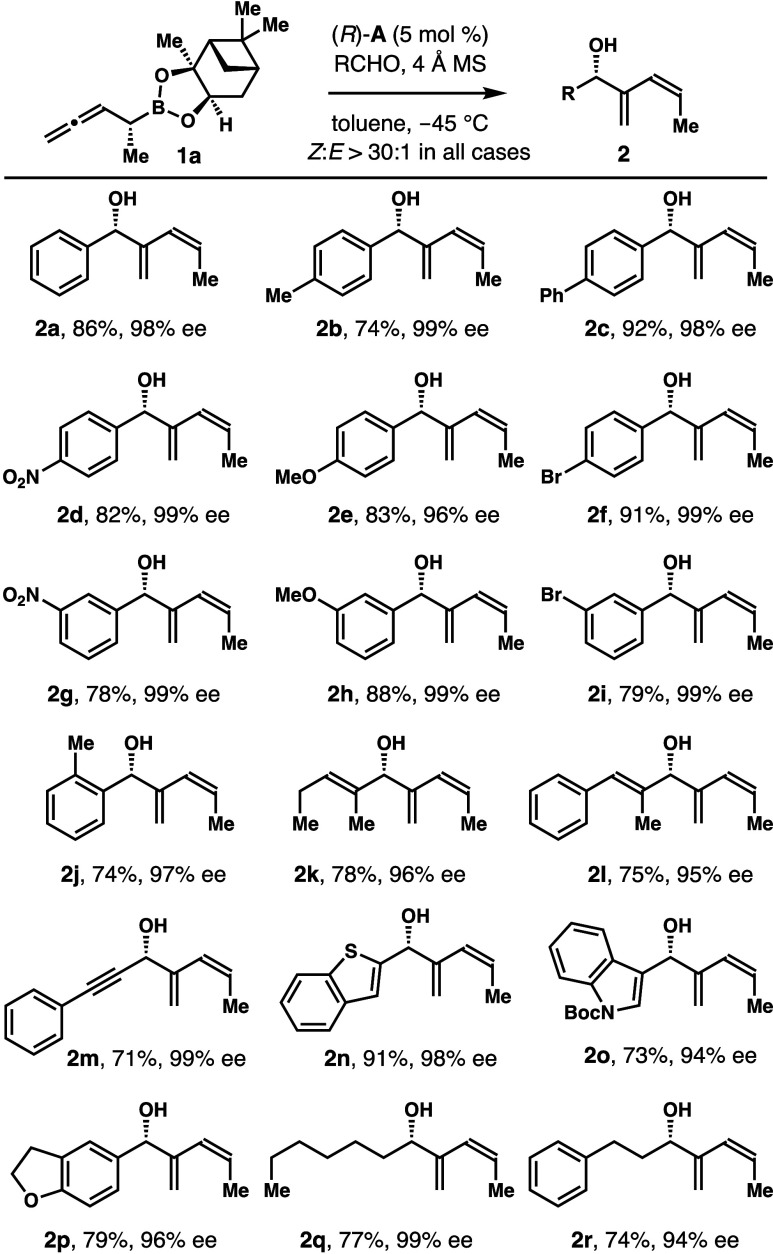
Scope of the Aldehyde for Acid (*R*)-**A**-Catalyzed Asymmetric Allylation with Boronate **1a**^–^ Reaction conditions: boronate **1a** (0.12 mmol, 1.2 equiv), aldehyde (0.1 mmol, 1.0 equiv), phosphoric acid (*R*)-**A** (5 mol %), 4 Å molecular sieves (50 mg), toluene (0.3 mL), −45 °C. Yields of isolated products **2** are listed. The *Z*/*E* ratios were determined by ^1^H NMR analyses of the crude reaction products. The enantiomeric excesses were determined by modified Mosher ester analyses.

To evaluate whether homoallenyl boronates with a substituent other than the methyl group at the α-position could also react with aldehydes to form 1,3-butadienyl-2-carbinols with high *Z*-selectivities, reactions of reagents **1b**–**d** with several representative aldehydes were conducted. As summarized in Scheme 4, the reactions tolerate several alkyl groups, including ethyl, *n*-propyl, and *i*-butyl groups, at the α-position of homoallenyl boronates **1**. Several aldehydes participated in the reactions with reagents **1b**–**d**, affording alcohol products **4a**–**i** in 73–94% yield with 97–99% ee. Again, the *Z*-isomers were obtained with excellent selectivities (>30:1) in all cases.

**Scheme 4:**
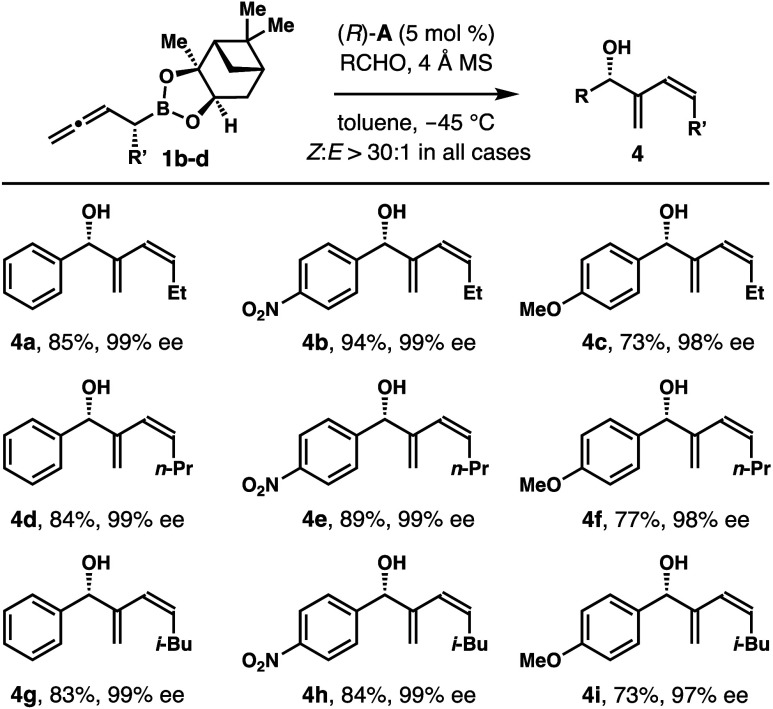
Chiral Phosphoric-Acid-Catalyzed Asymmetric Aldehyde Addition with Homoallenyl boronates **1b**–**d**^–^ Reaction conditions: allylboronate **1a** (0.12 mmol, 1.2 equiv), aldehyde (0.1 mmol, 1.0 equiv), phosphoric acid (*R*)-**A** (5 mol %), 4 Å molecular sieves (50 mg), toluene (0.3 mL), −45 °C. Yields of isolated products are listed. The *Z*/*E* ratios were determined by ^1^H NMR analyses of the crude reaction products. The enantiomeric excesses were determined by modified Mosher ester analysis.

The reaction of allylboronate **5** with benzaldehyde formed a 1.5:1 mixture of homoallylic alcohols **6** and **7**. The observed 6:1 *Z*-selectivity in the uncatalyzed reaction with boronate **1a** is somewhat unexpected (entry 1, Scheme 2). To establish the origin of such selectivity, we analyzed the transition states of these reactions. As depicted in Scheme 5, in transition state **TS**-**2** that leads to *E*-isomer **7**, the α-methyl group adopts a pseudoequatorial position. Such a spatial arrangement will suffer unfavorable gauche interactions between the methyl group of reagent **5** and the pinanediol group on boron (shown with a red arrow in **TS**-**2**). In comparison, in transition state **TS**-**1** that forms *Z*-isomer **6**, the methyl group is oriented in a pseudoaxial position, and A^1,3^ strain between the methyl group and the vinyl hydrogen is developed.^15^ Two competing transition states, **TS**-**1** and **TS**-**2**, are similar in energy. Therefore, the reaction forms a mixture of *Z* and *E* isomers, **6** and **7**, with low selectivity. The energy difference of **TS**-**1** and **TS**-**2** is estimated to be 0.24 kcal/mol at 25 °C. The energy penalty for the A^1,3^ strain in **TS**-**1** is about 1 kcal/mol. Therefore, the energy penalty for the gauche interactions in **TS**-**2** is estimated to be 1.24 kcal/mol at 25 °C.

**Scheme 5:**
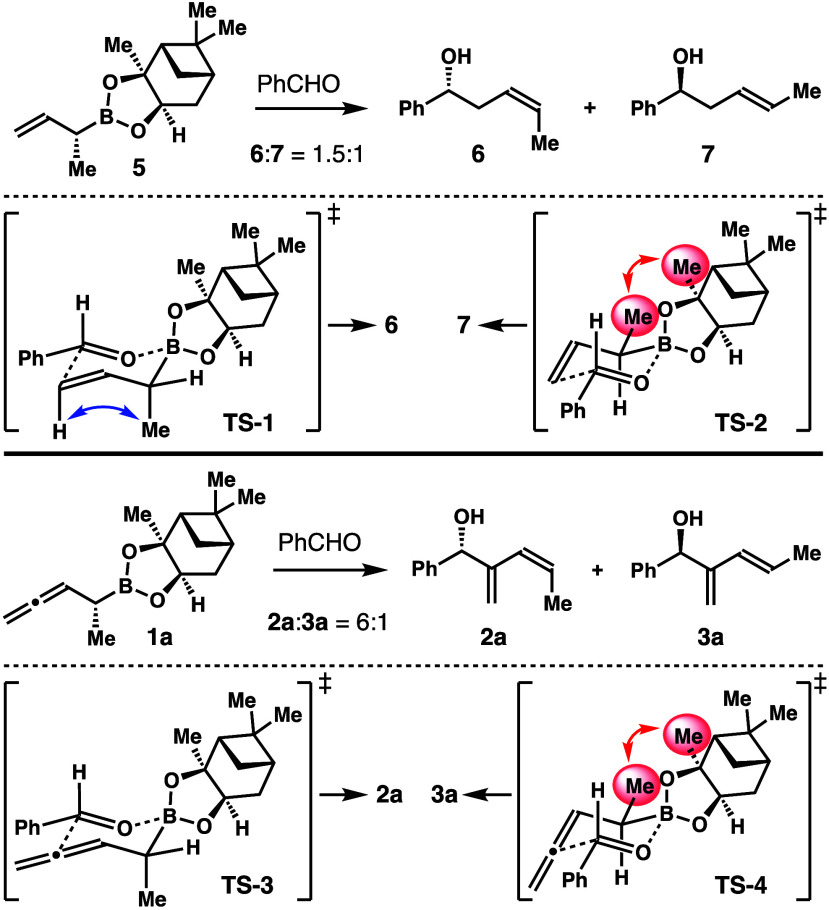
Transition State Analyses of Aldehyde Addition with Boron Reagents **5** and **1a**

In the uncatalyzed reaction of **1a** with benzaldehyde, two competing transition states, **TS**-**3** and **TS**-**4**, lead to the formation of *Z*-isomer **2a** and *E*-isomer **3a**, respectively (Scheme 5). Close inspection of transition states **TS**-**4** and **TS**-**2** revealed that similar unfavorable gauche interactions also exist in **TS**-**4**. By contrast, the A^1,3^ strain as in **TS**-**1** is not present in **TS**-**3**, owing to the lack of the vinyl hydrogen. Therefore, the energy difference of **TS**-**3** and **TS**-**4** is estimated to be 1.24 kcal/mol at 25 °C due to the gauche interactions in **TS**-**4**. The observed 6:1 selectivity of **2a** and **3a** in the reaction with **1a** corresponds to a 1.06 kcal/mol energy difference at 25 °C, which is in good accord with the 1.24 kcal/mol caused by the gauche interactions.

In the chiral phosphoric acid (*R*)-**A**-catalyzed reaction of **1a**, the asymmetric induction from the catalyst is the same as the inherent selectivity of reagent **1a** as in transition state **TS**-**5**. The additive effect of these two stereodirecting factors is estimated to be at least 3 kcal/mol at 25 °C. Moreover, the steric interactions between the pinanediol group on boron and the acid catalyst (similar to the pinacol boronate case) further destabilize transition state **TS**-**6** (Scheme 6). Therefore, acid (*R*)-**A**-catalyzed reaction of **1a** with aldehydes proceeded with the favored transition state **TS**-**5** to give *Z*-isomers **2** with excellent selectivities.

**Scheme 6:**
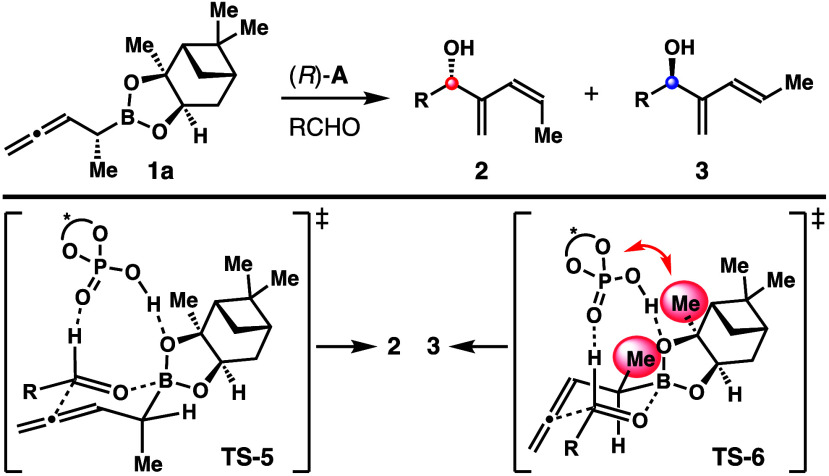
Transition State Analyses of Chiral Phosphoric-Acid-Catalyzed Aldehyde Addition with Reagent **1a**

In summary, we developed a chiral phosphoric acid (*R*)-**A**-catalyzed reaction of homoallenyl boronates with aldehydes. A variety of 1,3-butadienyl-2-carbinols were obtained with excellent *Z*-selectivities and enantioselectivities. Synthetic applications of the method will be reported in due course.

## Data Availability

The data underlying this study are available in the published article and its Supporting Information.

## Supporting Information Available

Experimental procedures, spectra for all new compounds (PDF)

## References

[ref1] aKobayashi J.; Tsuda M.; Ishibashi M.; Shigemori H.; Yamasu T.; Hirota H.; Sasaki T. Amphidinolide F, a new cytotoxic macrolide from the marine dinoflagellate Amphidinium sp. J. Antibiot. 1991, 44, 1259 10.7164/antibiotics.44.1259. 1761423

[ref2] aNicolaou K. C.; Bulger P. G.; Brenzovich W. E. Synthesis of *iso-*epoxy-amphidinolide N and de*s-e*poxy-caribenolide I structures. Revised strategy and final stages. Org. Biomol. Chem. 2006, 4, 2158 10.1039/b602021f. 16729127

[ref3] aTrost B. M.; Papillon J. P. N. Alkene–Alkyne Coupling as a Linchpin: An efficient and convergent synthesis of amphidinolide P. J. Am. Chem. Soc. 2004, 126, 13618 10.1021/ja045449x. 15493910

[ref4] aWang M.; Gao S.; Chen M. Stereoselective syntheses of (*E*)-γ′,δ-bisboryl-substituted *syn*-homoallylic alcohols via chemoselective aldehyde allylboration. Org. Lett. 2019, 21, 2151 10.1021/acs.orglett.9b00461. 30864811

[ref5] aLachance H.; Hall D. G. Allylboration of carbonyl compounds. Org. React. 2009, 73, 1 10.1002/0471264180.or073.01.

[ref6] aMatteson D. S.; Ray R. alpha-Chloro boronic esters from homologation of boronic esters. J. Am. Chem. Soc. 1980, 102, 7590 10.1021/ja00545a046.

[ref7] aDale J. A.; Mosher H. S. Nuclear magnetic resonance enantiomer regents. Configurational correlations via nuclear magnetic resonance chemical shifts of diastereomeric mandelate, O-methylmandelate, and.alpha.-methoxy-.alpha.-trifluoromethylphenyl-acetate (MTPA) esters. J. Am. Chem. Soc. 1973, 95, 512 10.1021/ja00783a034.

[ref8] aKennedy J. W. J.; Hall D. G. Dramatic rate enhancement with preservation of stereospecificity in the first metal-catalyzed additions of allylboronates. J. Am. Chem. Soc. 2002, 124, 11586 10.1021/ja027453j. 12296710

[ref9] aPeng F.; Hall D. G. Simple, stable, and versatile double-allylation reagents for the stereoselective preparation of skeletally diverse compounds. J. Am. Chem. Soc. 2007, 129, 3070 10.1021/ja068985t. 17315879

[ref10] aJain P.; Antilla J. C. Chiral Brønsted acid-catalyzed allylboration of aldehydes. J. Am. Chem. Soc. 2010, 132, 11884 10.1021/ja104956s. 20690662 PMC2928988

[ref11] aJain P.; Wang H.; Houk K. N.; Antilla J. C. Brønsted acid catalyzed asymmetric propargylation of aldehydes. Angew. Chem., Int. Ed. 2012, 51, 1391 10.1002/anie.201107407. PMC3334334 22223476

[ref12] aReddy L. R. Chiral Brønsted acid catalyzed enantioselective allenylation of aldehydes. Chem. Commun. 2012, 48, 9189 10.1039/c2cc34371a. 22872133

[ref13] aGrayson M. N.; Pellegrinet S. C.; Goodman J. M. Mechanistic insights into the BINOL-derived phosphoric acid-catalyzed asymmetric allylboration of aldehydes. J. Am. Chem. Soc. 2012, 134, 2716 10.1021/ja210200d. 22239113

[ref14] aMiura T.; Nakahashi J.; Zhou W.; Shiratori Y.; Stewart S. G.; Murakami M. Enantioselective synthesis of *anti*-1,2-oxaborinan-3-enes from aldehydes and 1,1-di(boryl)alk-3-enes using ruthenium and chiral phosphoric acid catalysts. J. Am. Chem. Soc. 2017, 139, 10903 10.1021/jacs.7b06408. 28708391

[ref15] Hoffmann R. W. Allylic 1,3-strain as a controlling factor in stereoselective transformations. Chem. Rev. 1989, 89, 1841 10.1021/cr00098a009.

