# Supplementary material for: Stereo- and Enantioselective Syntheses of (Z)-1,3-Butadienyl-2-carbinols via Brønsted Acid Catalysis
Source: Org Lett. 2025 Jan 9;27(3):887–91. doi: 10.1021/acs.orglett.4c04663 (PMC11773566; doi:10.1021/acs.orglett.4c04663)
Supplement: Supplementary file 1 — ol4c04663_si_001.pdf [file ol4c04663_si_001.pdf]

Supporting Information

Stereo- and Enantioselective Syntheses of (*Z*)-1,3-Butadienyl-2-carbinols via Brønsted Acid Catalysis

*Ming Chen**

Department of Chemistry, Virginia Tech

E-mail: mzc0102@vt.edu

Table of Contents

S2	General Experimental Details
S3	General Procedure for the Syntheses of Allenylboronates **1**
S4	General Procedure for the Syntheses of Alcohols **2** or **4**
S5	Tabulated Spectroscopic Data
S13	References
S14	Assignment of the Absolute Configuration
S16	^{1}H and ^{13}C Spectra of New Compounds

General Experimental Details. All reaction solvents were purified before use. Dichloromethane, THF and toluene were purified by passing through a solvent column composed of activated A-1 alumina. Unless indicated otherwise, all reactions were conducted under an atmosphere of argon using flame-dried or oven-dried (140 °C) glassware. The term “concentrated under reduced pressure” refers to the removal of solvents and other volatile materials using a rotary evaporator with the water bath temperature below 30 °C, followed by the removal of residual solvents at high vacuum ($<$ 0.2 mbar).

Proton nuclear magnetic resonance (^{1}H NMR) spectra were acquired on commercial instruments at 400, 500 and 600 MHz. Carbon-13 nuclear magnetic resonance (^{13}C NMR) spectra were acquired at 101, 126 and 151 MHz. The proton signal for the residual non-deuterated solvent (δ 7.26 for $CHCl_3$) was used as an internal reference for ^{1}H NMR spectra. For ^{13}C NMR spectra, chemical shifts are reported relative to the δ 77.36 resonance of $CHCl_3$. Coupling constants are reported in Hz. High-resolution mass spectra were recorded on a commercial high-resolution mass spectrometer (mass analyzer type: QTOF). Optical rotations of the products were measured by using a Perkin Elmer 241 Automatic Polarimeter from Rudolph Research Analytical.

Analytical thin layer chromatography (TLC) was performed on Kieselgel 60 F254 glass plates precoated with a 0.25 mm thickness of silica gel. The TLC plates were visualized with UV light and/or by staining with Hanessian solution (ceric sulfate and ammonium molybdate in aqueous sulfuric acid) or $KMnO_4$. Column chromatography was generally performed using Kieselgel 60 (230-400 mesh) silica gel, typically using a 50-100:1 weight ratio of silica gel to crude product.

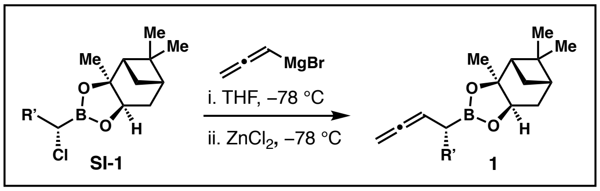

General procedure for syntheses of allenylboronates 1: To a solution of boronic ester **SI-1** (1.0 mmol) in dry THF (5.0 mL) was added freshly prepared allenylmagnesium bromide[1] (1.0 mmol, in anhydrous THF, 1 equiv) at −78 °C. After stirring at −78 °C for 30 min, $ZnCl_2$ (1 mL, 1.0 mmol, 1.0 M in Et_2O, 1 equiv) was added. The reaction mixture was kept stirring at −78 °C for 3 h. Hexane (15 mL) was added, and the resulting mixture was allowed to warm to ambient temperature. The reaction mixture was filtered through a pad of silica gel. The filtrate was concentrated under reduced pressure. Purification of the crude product was performed by flash column chromatography to provide boronate **1**.

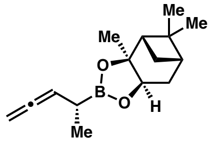

(3a*S*,4*R*,6*R*,7a*R*)-3a,5,5-trimethyl-2-((*R*)-penta-3,4-dien-2-yl)hexahydro-4,6-methanobenzo[*d*][1,3,2]dioxaborole (**1a**) Prepared according to the general procedure. The crude mixture was purified by flash column chromatography (gradient elution with hexane and Et_2O, 50:1 to 10:1) to give compound **1a** as colorless oil in 89% yield (220 mg). ^{1}H NMR (600 MHz, $CDCl_3$) δ 5.29 (dt, *J* = 6.7, 6.7 Hz, 1H), 4.72 (dd, *J* = 6.5, 3.7 Hz, 2H), 4.28 (d, *J* = 8.7 Hz, 1H), 2.31 – 2.35 (m, 1H), 2.21 (dt, *J* = 11.7, 6.1 Hz, 1H), 2.05 (t, *J* = 5.5 Hz, 1H), 1.89 – 1.92 (m, 1H), 1.82 – 1.86 (m, 2H), 1.38 (s, 3H), 1.28 (s, 3H), 1.11 (d, *J* = 7.2 Hz, 3H), 1.10 (d, *J* = 10.9 Hz, 1H), 0.83 (s, 3H). ^{13}C NMR (151 MHz, CDCl3) δ 208.1, 93.3, 86.1, 78.2, 76.4, 51.5, 39.7, 38.5, 35.8, 28.9, 27.4, 26.7, 24.3, 15.1. HRMS (EI$^+$) *m/z*: [M]$^+$ calcd. for $C_{15}H_{23}BO_2$ 246.1791; found: 246.1802.

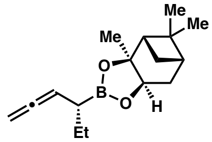

(3a*S*,4*R*,6*R*,7a*R*)-2-((*R*)-hexa-4,5-dien-3-yl)-3a,5,5-trimethylhexahydro-4,6-methano benzo[*d*][1,3,2]dioxaborole (**1b**) Prepared according to the general procedure. The crude mixture was purified by flash column chromatography (gradient elution with hexane and Et_2O, 50:1 to 10:1) to give compound **1b** as colorless oil in 82% yield (213 mg). ^{1}H NMR (600 MHz, $CDCl_3$) δ 5.16 (dt, *J* = 6.9, 6.9 Hz, 1H), 4.69 (dd, *J* = 6.4, 1.9 Hz, 2H), 4.29 (d, *J* = 8.5 Hz, 1H), 2.32 – 2.34 (m, 1H), 2.19 – 2.23 (m, 1H), 2.06 (t, *J* = 5.2 Hz, 1H), 1.91 (brs, 1H), 1.84 (d, *J* = 14.7 Hz, 1H), 1.68 – 1.72 (m, 1H), 1.60 – 1.64 (m, 1H), 1.46 – 1.53 (m, 1H), 1.38 (s, 3H), 1.28 (s, 3H), 1.13 (d, *J* = 10.9 Hz, 1H), 0.96 (t, *J* = 7.2 Hz, 3H), 0.84 (s, 3H). ^{13}C NMR (151 MHz, $CDCl_3$) δ 208.5, 91.4, 86.1, 78.0, 75.6, 51.4, 39.7, 38.5, 35.8, 29.0,

27.4, 26.8, 24.4, 24.1, 14.0. HRMS (EI^+) *m/z*: $[M]^+$ calcd. for $C_{16}H_{25}BO_2$ 260.1948; found: 260.1961.

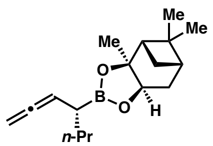

(3a*S*,4*R*,6*R*,7a*R*)-2-((*R*)-hepta-1,2-dien-4-yl)-3a,5,5-trimethylhexahydro-4,6-methanobenzo[*d*][1,3,2]dioxaborole (**1c**) Prepared according to the general procedure. The crude mixture was purified by flash column chromatography (gradient elution with hexane and Et_2O, 50:1 to 10:1) to give compound **1c** as colorless oil in 72% yield (198 mg). ^1H NMR (600 MHz, $CDCl_3$) δ 5.15 (dt, *J* = 6.7, 6.7 Hz, 1H), 4.68 (dd, *J* = 6.6, 2.5 Hz, 2H), 4.28 (dd, *J* = 8.7, 1.6 Hz, 1H), 2.31 – 2.36 (m, 1H), 2.20 – 2.22 (m, 1H), 2.06 (t, *J* = 5.4 Hz, 1H), 1.90 – 1.92 (m, 1H), 1.84 – 1.86 (m, 1H), 1.74 – 1.83 (m, 1H), 1.52 – 1.55 (m, 1H), 1.32 – 1.48 (m, 3H), 1.38 (s, 3H), 1.28 (s, 3H), 1.12 (d, *J* = 10.9 Hz, 1H), 0.89 (t, *J* = 7.2 Hz, 3H), 0.83 (s, 3H). ^{13}C NMR (151 MHz, $CDCl_3$) δ 208.4, 91.5, 86.1, 78.0, 75.6, 51.4, 39.7, 38.5, 35.8, 33.1, 29.0, 27.4, 26.7, 24.4, 22.5, 14.5. HRMS (EI^+) *m/z*: $[M]^+$ calcd. for $C_{17}H_{27}BO_2$ 274.2104; found: 274.2118.

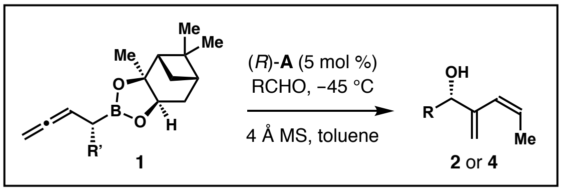

General procedure for the syntheses of alcohols 2 or 4: To a reaction flask containing a stirring bar and freshly activated 4 Å MS (50 mg) was added phosphoric acid (*R*)-**A** (4 mg, 5 mol %). Toluene (0.3 mL) was added to the flask followed by addition of boronate **1** (0.12 mmol, 1.2 equiv). The reaction flask was placed in a −45 °C cold bath and stirred for 10 min. Then a solution of freshly distilled aldehyde (0.1 mmol, 1.0 equiv, if it is a liquid) in toluene (0.2 mL) was added slowly *via* a microliter syringe. The reaction mixture was kept stirring at −45 °C for 48 h. After complete consumption of the aldehyde, 3N NaOH (1 mL) was added followed by slow addition of 30% H_2O_2 (0.5 mL) at 0 °C. The resulting mixture was stirred vigorously at 0 °C for 2 h. Brine (1 mL) and Et_2O (0.5 mL) were added, the organic layer was separated and the aqueous layer was extracted with Et_2O (3 x 1 mL). The combined organic extracts were dried over anhydrous sodium sulfate and filtered. The filtrate was concentrated under reduced pressure. Purification of the crude product was performed by flash column chromatography (gradient elution with hexane and Et_2O) provided products **2** or **4**. The enantiopurities of **2** or **4** were determined by Mosher ester analysis.[2]

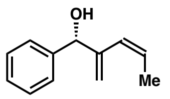

(*R*,*Z*)-2-methylene-1-phenylpent-3-en-1-ol (2a) Prepared according to the general procedure from **1a**. The crude mixture was purified by flash column chromatography (gradient elution with hexane and Et_2O, 20:1 to 5:1) to give the title compound as colorless oil in 86% yield (15 mg). A 1 mmol-scale reaction was also conducted with PhCHO (106 mg, 1 mmol), and product **2a** was isolated in 83% yield (145 mg). The enantiomeric excess was determined to be 98% ee by Mosher ester analysis; $[\alpha]_D^{25}$ = + 42.2 (c 0.40, $CHCl_3$); ^{1}H NMR (400 MHz, $CDCl_3$) δ 7.32 – 7.39 (m, 4H), 7.25 – 7.29 (m, 1H), 5.64 – 5.72 (m, 2H), 5.49 (s, 1H), 5.21 (d, *J* = 4.0 Hz, 1H), 5.13 (s, 1H), 1.98 (d, *J* = 4.1 Hz, 1H), 1.75 (d, *J* = 5.1 Hz, 3H). ^{13}C NMR (151 MHz, $CDCl_3$) δ 146.4, 142.1, 129.6, 128.7, 127.9, 126.8 (two overlapping ^{13}C signals), 114.3, 77.6, 15.2. HRMS (EI$^+$) *m/z*: [M]$^+$ calcd. for $C_{12}H_{14}O$ 174.1045; found: 174.1039.

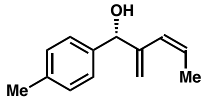

(*R*,*Z*)-2-methylene-1-(*p*-tolyl)pent-3-en-1-ol (2b) Prepared according to the general procedure from **1a**. The crude mixture was purified by flash column chromatography (gradient elution with hexane and Et_2O, 20:1 to 5:1) to give the title compound as colorless oil in 74% yield (14 mg). The enantiomeric excess was determined to be 99% ee by Mosher ester analysis; $[\alpha]_D^{25}$ = + 51.7 (c 0.40, $CHCl_3$); ^{1}H NMR (600 MHz, $CDCl_3$) δ 7.26 (d, *J* = 7.7 Hz, 2H), 7.15 (d, *J* = 7.8 Hz, 2H), 5.64 – 5.71 (m, 2H), 5.49 (s, 1H), 5.17 (s, 1H), 5.12 (s, 1H), 2.34 (s, 3H), 1.96 (brs, 1H), 1.76 (d, *J* = 4.4 Hz, 3H). ^{13}C NMR (151 MHz, $CDCl_3$) δ 146.4, 139.2, 137.7, 129.5, 129.4, 126.9, 126.8, 114.1, 77.4, 21.5, 15.2. HRMS (EI$^+$) *m/z*: [M]$^+$ calcd. for $C_{13}H_{16}O$ 188.1201; found: 188.1192.

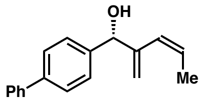

(*R*,*Z*)-1-([1,1'-biphenyl]-4-yl)-2-methylenepent-3-en-1-ol (2c) Prepared according to the general procedure from **1a**. The crude mixture was purified by flash column chromatography (gradient elution with hexane and Et_2O, 20:1 to 5:1) to give the title compound as colorless oil in 92% yield (23 mg). The enantiomeric excess was determined to be 98% ee by Mosher ester analysis; $[\alpha]_D^{25}$ = + 44.5 (c 0.85, $CHCl_3$); ^{1}H NMR (600 MHz, $CDCl_3$) δ 7.57 – 7.60 (m, 4H), 7.43 – 7.46 (m, 4H), 7.34 – 7.36 (m, 1H), 5.69 – 5.75 (m, 2H), 5.54 (s, 1H), 5.26 (s, 1H), 5.17 (s, 1H), 2.06 (brs, 1H), 1.78 (d, *J* = 5.1 Hz, 3H). ^{13}C NMR (151 MHz, $CDCl_3$) δ 146.3, 141.2, 141.1, 140.8, 129.8, 129.1, 127.6, 127.43, 127.40, 127.2, 126.7, 114.5, 77.4, 15.3. HRMS (EI$^+$) *m/z*: [M]$^+$ calcd. for $C_{18}H_{18}O$ 250.1358; found: 250.1365.

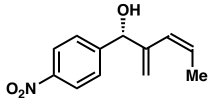

(*R*,*Z*)-2-methylene-1-(4-nitrophenyl)pent-3-en-1-ol (2d) Prepared according to the general procedure from **1a**. The crude mixture was

purified by flash column chromatography (gradient elution with hexane and Et_2O, 20:1 to 2:1) to give the title compound as colorless oil in 82% yield (18 mg). The enantiomeric excess was determined to be 99% ee by Mosher ester analysis; $[\alpha]_D^{25}$ = – 9.4 (c 0.58, $CHCl_3$); ^{1}H NMR (400 MHz, $CDCl_3$) δ 8.19 (d, *J* = 8.5 Hz, 2H), 7.56 (d, *J* = 8.5 Hz, 2H), 5.70 – 5.78 (m, 1H), 5.64 (d, *J* = 11.9 Hz, 1H), 5.49 (s, 1H), 5.32 (s, 1H), 5.17 (s, 1H), 2.11 (brs, 1H), 1.72 (dd, *J* = 6.8, 1.6 Hz, 3H). ^{13}C NMR (151 MHz, $CDCl_3$) δ 149.4, 147.5, 145.6, 131.0, 127.4, 125.6, 123.8, 116.0, 77.1, 15.2. HRMS (EI^+) *m/z*: $[M]^+$ calcd. for $C_{12}H_{13}NO_3$ 219.0895; found: 219.0906.

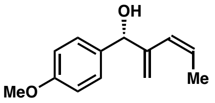

(*R*,*Z*)-1-(4-methoxyphenyl)-2-methylenepent-3-en-1-ol (2e) Prepared according to the general procedure from **1a**. The crude mixture was purified by flash column chromatography (gradient elution with hexane and Et_2O, 20:1 to 2:1) to give the title compound as colorless oil in 83% yield (17 mg). The enantiomeric excess was determined to be 96% ee by Mosher ester analysis; $[\alpha]_D^{25}$ = + 53.9 (c 0.55, $CHCl_3$);^{1}H NMR (600 MHz, $CDCl_3$) δ 7.29 (d, *J* = 8.5 Hz, 2H), 6.87 (d, *J* = 8.6 Hz, 2H), 5.66 – 5.69 (m, 2H), 5.49 (s, 1H), 5.15 (s, 1H), 5.12 (s, 1H), 3.80 (s, 3H), 1.94 (brs, 1H), 1.76 (d, *J* = 4.8 Hz, 3H). ^{13}C NMR (151 MHz, $CDCl_3$) δ 159.3, 146.5, 134.3, 129.4, 128.2, 127.0, 114.0, 113.9, 77.1, 55.6, 15.2. HRMS (EI^+) *m/z*: $[M]^+$ calcd. for $C_{13}H_{16}O_2$ 204.1150; found: 204.1152.

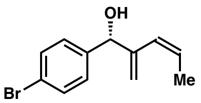

(*R*,*Z*)-1-(4-bromophenyl)-2-methylenepent-3-en-1-ol (2f) Prepared according to the general procedure from **1a**. The crude mixture was purified by flash column chromatography (gradient elution with hexane and Et_2O, 20:1 to 4:1) to give the title compound as colorless oil in 91% yield (23 mg). The enantiomeric excess was determined to be 99% ee by Mosher ester analysis; $[\alpha]_D^{25}$ = + 24.1 (c 0.85, $CHCl_3$); ^{1}H NMR (600 MHz, $CDCl_3$) δ 7.46 (d, *J* = 8.4 Hz, 2H), 7.25 (d, *J* = 8.6 Hz, 2H), 5.68 – 5.73 (m, 1H), 5.64 (d, *J* = 11.7 Hz, 1H), 5.47 (s, 1H), 5.17 (s, 1H), 5.13 (s, 1H), 2.04 (brs, 1H), 1.74 (dd, *J* = 6.9, 1.8 Hz, 3H). ^{13}C NMR (151 MHz, $CDCl_3$) δ 146.0, 141.1, 131.7, 130.1, 128.5, 126.3, 121.7, 114.8, 77.1, 15.2. HRMS (EI^+) *m/z*: $[M]^+$ calcd. for $C_{12}H_{13}OBr$ 252.0150; found: 252.0148.

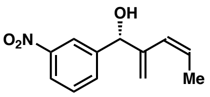

(*R*,*Z*)-2-methylene-1-(3-nitrophenyl)pent-3-en-1-ol (2g) Prepared according to the general procedure from **1a**. The crude mixture was purified by flash column chromatography (gradient elution with hexane and Et_2O, 20:1 to 2:1) to give the title compound as colorless oil in 78% yield (17 mg). The enantiomeric excess was determined to be 99% ee by Mosher ester analysis; $[\alpha]_D^{25}$ =

+ 4.4 (c 0.45, $CHCl_3$); ^{1}H NMR (600 MHz, $CDCl_3$) δ 8.26 (s, 1H), 8.13 (d, *J* = 8.0 Hz, 1H), 7.72 (d, *J* = 7.7 Hz, 1H), 7.51 (dd, *J* = 7.9, 7.9 Hz, 1H), 5.71 – 5.77 (m, 1H), 5.64 (d, *J* = 11.7 Hz, 1H), 5.52 (s, 1H), 5.32 (s, 1H), 5.18 (s, 1H), 2.18 (brs, 1H), 1.73 (dd, *J* = 7.0, 1.9 Hz, 3H). ^{13}C NMR (151 MHz, $CDCl_3$) δ 148.5, 145.6, 144.2, 132.8, 130.9, 129.5, 125.6, 122.9, 121.7, 115.9, 76.9, 15.2. HRMS (EI^+) *m/z*: $[M]^+$ calcd. for $C_{12}H_{13}NO_3$ 219.0895; found: 219.0902.

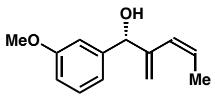

(*R*,*Z*)-1-(3-methoxyphenyl)-2-methylenepent-3-en-1-ol (2h) Prepared according to the general procedure from **1a**. The crude mixture was purified by flash column chromatography (gradient elution with hexane and Et_2O, 20:1 to 2:1) to give the title compound as colorless oil in 88% yield (18 mg). The enantiomeric excess was determined to be 99% ee by Mosher ester analysis; $[\alpha]_D^{25}$ = + 35.9 (c 0.60, $CHCl_3$); ^{1}H NMR (600 MHz, $CDCl_3$) δ 7.24 – 7.27 (m, 1H), 6.93 – 6.96 (m, 2H), 6.81 (dd, *J* = 8.1, 2.7 Hz, 1H), 5.66 – 5.72 (m, 2H), 5.49 (s, 1H), 5.18 (s, 1H), 5.13 (s, 1H), 3.80 (s, 3H), 2.00 (brs, 1H), 1.76 (d, *J* = 5.3 Hz, 3H). ^{13}C NMR (151 MHz, $CDCl_3$) δ 159.9, 146.2, 143.8, 129.74, 129.69, 126.7, 119.2, 114.5, 113.4, 112.1, 77.5, 55.5, 15.2. HRMS (EI^+) *m/z*: $[M]^+$ calcd. for $C_{13}H_{16}O_2$ 204.1150; found: 204.1138.

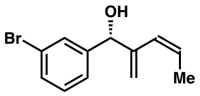

(*R*,*Z*)-1-(3-bromophenyl)-2-methylenepent-3-en-1-ol (2i) Prepared according to the general procedure from **1a**. The crude mixture was purified by flash column chromatography (gradient elution with hexane and Et_2O, 20:1 to 4:1) to give the title compound as colorless oil in 79% yield (20 mg). The enantiomeric excess was determined to be 99% ee by Mosher ester analysis; $[\alpha]_D^{25}$ = + 10.4 (c 0.45, $CHCl_3$); ^{1}H NMR (600 MHz, $CDCl_3$) δ 7.54 (s, 1H), 7.40 (d, *J* = 7.5 Hz, 1H), 7.30 (d, *J* = 7.6 Hz, 1H), 7.21 (dd, *J* = 7.8, 7.8 Hz, 1H), 5.70 – 5.75 (m, 1H), 5.64 (d, *J* = 11.7 Hz, 1H), 5.48 (s, 1H), 5.18 (d, *J* = 3.8 Hz, 1H), 5.14 (s, 1H), 2.02 (d, *J* = 4.1 Hz, 1H), 1.75 (dd, *J* = 7.0, 1.8 Hz, 3H). ^{13}C NMR (151 MHz, $CDCl_3$) δ 145.9, 144.4, 130.9, 130.3, 130.2, 129.8, 126.2, 125.4, 122.8, 115.1, 77.1, 15.2. HRMS (EI^+) *m/z*: $[M]^+$ calcd; for $C_{12}H_{13}OBr$ 252.0150, found: 252.0146.

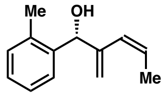

(*R*,*Z*)-2-methylene-1-(*o*-tolyl)pent-3-en-1-ol (2j) Prepared according to the general procedure from **1a**. The crude mixture was purified by flash column chromatography (gradient elution with hexane and Et_2O, 20:1 to 5:1) to give the title compound as colorless oil in 74% yield (14 mg). The enantiomeric excess was determined to be 97% ee by Mosher ester analysis; $[\alpha]_D^{25}$ = + 54.0 (c 0.70,

CHCl$_3$); ^{1}H NMR (600 MHz, CDCl$_3$) δ 7.43 (d, J = 7.0 Hz, 1H), 7.18 – 7.23 (m, 2H), 7.14 (d, J = 6.9 Hz, 1H), 5.73 (d, J = 11.8 Hz, 1H), 5.65 – 5.70 (m, 1H), 5.39 (s, 2H), 5.15 (s, 1H), 2.35 (s, 3H), 1.90 (brs, 1H), 1.77 (d, J = 6.7 Hz, 3H). ^{13}C NMR (151 MHz, CDCl$_3$) δ 145.3, 139.6, 135.9, 130.5, 128.9, 127.6, 127.2, 126.3, 126.1, 114.6, 73.8, 19.4, 14.9. HRMS (EI$^+$) *m/z*: [M]$^+$ calcd. for $C_{13}H_{16}O$ 188.1201; found: 188.1205.

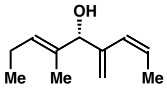

(*S*,2*Z*,6*E*)-6-methyl-4-methylenenona-2,6-dien-5-ol (2k) Prepared according to the general procedure from **1a**. The crude mixture was purified by flash column chromatography (gradient elution with hexane and Et$_2$O, 20:1 to 5:1) to give the title compound as colorless oil in 78% yield (13 mg). The enantiomeric excess was determined to be 96% ee by Mosher ester analysis; $[\alpha]_D^{25}$ = + 40.9 (c 0.65, CHCl$_3$); ^{1}H NMR (600 MHz, CDCl$_3$) δ 5.67 – 5.73 (m, 2H), 5.49 (t, J = 6.9 Hz, 1H), 5.39 (s, 1H), 5.09 (s, 1H), 4.46 (s, 1H), 2.02 – 2.07 (m, 2H), 1.77 – 1.81 (m, 3H), 1.65 (brs, 1H), 1.52 (s, 3H), 0.97 (t, J = 7.5 Hz, 3H). ^{13}C NMR (151 MHz, CDCl$_3$) δ 144.7, 134.1, 129.4, 128.5, 126.9, 113.6, 80.0, 21.1, 14.9, 14.1, 11.7. HRMS (EI$^+$) *m/z*: [M]$^+$ calcd. for $C_{11}H_{18}O$ 166.1358; found: 166.1363.

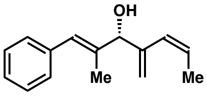

(*S*,1*E*,5*Z*)-2-methyl-4-methylene-1-phenylhepta-1,5-dien-3-ol (2l) Prepared according to the general procedure from **1a**. The crude mixture was purified by flash column chromatography (gradient elution with hexane and Et$_2$O, 20:1 to 5:1) to give the title compound as colorless oil in 75% yield (16 mg). The enantiomeric excess was determined to be 95% ee by Mosher ester analysis; $[\alpha]_D^{25}$ = + 23.5 (c 0.50, CHCl$_3$); ^{1}H NMR (600 MHz, CDCl$_3$) δ 7.32 – 7.35 (m, 2H), 7.28 – 7.30 (m, 2H), 7.21 – 7.23 (m, 1H), 6.61 (s, 1H), 5.81 (d, J = 11.7 Hz, 1H), 5.72 – 5.77 (m, 1H), 5.47 (s, 1H), 5.16 (s, 1H), 4.65 (s, 1H), 1.85 (brs, 1H), 1.82 (dd, J = 6.9, 1.5 Hz, 3H), 1.80 (s, 3H). ^{13}C NMR (151 MHz, CDCl$_3$) δ 144.6, 138.1, 137.8, 129.4, 129.3, 128.4, 126.80, 126.76, 126.71, 114.8, 80.7, 15.2, 14.2. HRMS (EI$^+$) *m/z*: [M]$^+$ calcd. for $C_{15}H_{18}O$ 214.1358; found: 214.1354.

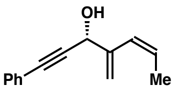

(*R*,*Z*)-4-methylene-1-phenylhept-5-en-1-yn-3-ol (2m) Prepared according to the general procedure from **1a**. The crude mixture was purified by flash column chromatography (gradient elution with hexane and Et$_2$O, 20:1 to 5:1) to give the title compound as colorless oil in 71% yield (14 mg). The enantiomeric excess was determined to be 99% ee by Mosher ester analysis; $[\alpha]_D^{25}$ = – 6.2 (c 0.40, CHCl$_3$); ^{1}H NMR (600 MHz, CDCl$_3$) δ 7.44 – 7.45 (m, 2H), 7.30 – 7.33 (m, 3H), 6.08 (d, J = 11.6 Hz, 1H), 5.88 (dq, J = 11.6, 7.1 Hz, 1H), 5.59 (s, 1H), 5.14 (s, 1H),

5.08 (s, 1H), 2.08 (brs, 1H), 1.85 (dd, *J* = 7.1, 2.0 Hz, 3H). ^{13}C NMR (151 MHz, $CDCl_3$) δ 143.6, 132.0, 130.5, 128.9, 128.6, 126.3, 122.7, 115.6, 88.1, 86.5, 66.7, 15.3. HRMS (EI^+) *m/z*: $[M]^+$ calcd. for $C_{14}H_{14}O$ 198.1045; found: 198.1053.

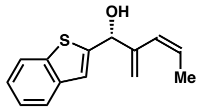

(*R*,*Z*)-1-(benzo[*b*]thiophen-2-yl)-2-methylenepent-3-en-1-ol (2n) Prepared according to the general procedure from **1a**. The crude mixture was purified by flash column chromatography (gradient elution with hexane and Et_2O, 20:1 to 2:1) to give the title compound as colorless oil in 91% yield (21 mg). The enantiomeric excess was determined to be 98% ee by Mosher ester analysis; $[\alpha]_D^{25}$ = + 35.0 (c 0.75, $CHCl_3$); ^{1}H NMR (600 MHz, $CDCl_3$) δ 7.80 (d, *J* = 7.9 Hz, 1H), 7.71 (d, *J* = 7.7 Hz, 1H), 7.28 – 7.35 (m, 2H), 7.22 (s, 1H), 5.83 (d, *J* = 11.8 Hz, 1H), 5.74 – 5.80 (dt, *J* = 11.7, 6.6 Hz, 1H), 5.58 (s, 1H), 5.51 (s, 1H), 5.21 (s, 1H), 2.29 (brs, 1H), 1.82 (dd, *J* = 6.8, 1.7 Hz, 3H). ^{13}C NMR (151 MHz, $CDCl_3$) δ 147.3, 145.4, 140.1, 139.8, 130.6, 126.0, 124.53, 124.49, 123.9, 122.8, 121.5, 115.2, 74.3, 15.4. HRMS (EI^+) *m/z*: $[M]^+$ calcd. for $C_{14}H_{14}OS$ 230.0765; found: 230.0768.

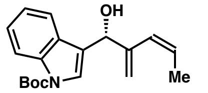

***tert*-butyl-(*R*,*Z*)-3-(1-hydroxy-2-methylenepent-3-en-1-yl)-1H-indole-1-carboxylate (2o)** Prepared according to the general procedure from **1a**. The crude mixture was purified by flash column chromatography (gradient elution with hexane and Et_2O, 20:1 to 2:1) to give the title compound as colorless oil in 73% yield (23 mg). The enantiomeric excess was determined to be 94% ee by Mosher ester analysis; $[\alpha]_D^{25}$ = + 10.7 (c 0.25, $CHCl_3$); ^{1}H NMR (600 MHz, $CDCl_3$) δ 8.14 (brs, 1H), 7.64 (d, *J* = 7.9 Hz, 1H), 7.54 (bs, 1H), 7.31 (dd, *J* = 7.8, 7.8 Hz, 1H), 7.22 (dd, *J* = 7.6, 7.6 Hz, 1H), 5.82 (d, *J* = 11.8 Hz, 1H), 5.70 – 5.75 (m, 1H), 5.61 (s, 1H), 5.46 (s, 1H), 5.24 (s, 1H), 2.05 (br, 1H), 1.81 (dd, *J* = 7.0, 1.9 Hz, 3H), 1.66 (s, 9H). ^{13}C NMR (151 MHz, $CDCl_3$) δ 149.8, 145.2, 136.1, 129.7, 128.9, 126.9, 124.8, 123.9, 122.9, 121.8, 120.2, 115.6, 115.2, 84.1, 71.6, 28.5, 15.3. HRMS (EI^+) *m/z*: $[M]^+$ calcd. for $C_{19}H_{23}O_3N$ 313.1678; found: 313.1689.

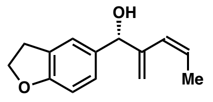

(*R*,*Z*)-1-(2,3-dihydrobenzofuran-5-yl)-2-methylenepent-3-en-1-ol (2p) Prepared according to the general procedure from **1a**. The crude mixture was purified by flash column chromatography (gradient elution with hexane and Et_2O, 20:1 to 2:1) to give the title compound as colorless oil in 79% yield (17 mg). The enantiomeric excess was determined to be 96% ee by Mosher ester analysis; $[\alpha]_D^{25}$ = + 56.8 (c 0.50, $CHCl_3$); ^{1}H NMR (600 MHz, $CDCl_3$) δ 7.21 (s, 1H), 7.09 (d, *J* = 8.0 Hz, 1H), 6.73 (d, *J* = 8.2 Hz, 1H), 5.65 – 5.70 (m, 2H), 5.50 (s, 1H), 5.12

(s, 2H), 4.56 (t, J = 8.7 Hz, 2H), 3.19 (t, J = 8.7 Hz, 2H), 1.96 (brs, 1H), 1.77 (d, J = 4.8 Hz, 3H). ^{13}C NMR (151 MHz, $CDCl_3$) δ 160.0, 146.6, 134.3, 129.3, 127.5, 127.11, 127.06, 123.7, 113.6, 109.2, 77.2, 71.7, 30.0, 15.3. HRMS (EI^+) *m/z*: $[M]^+$ calcd. for $C_{14}H_{16}O_2$ 216.1150; found: 216.1153.

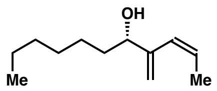

(*S*,*Z*)-4-methyleneundec-2-en-5-ol (2q) Prepared according to the general procedure from **1a**. The crude mixture was purified by flash column chromatography (gradient elution with hexane and Et_2O, 20:1 to 5:1) to give the title compound as colorless oil in 77% yield (14 mg). The enantiomeric excess was determined to be 99% ee by Mosher ester analysis; $[\alpha]_D^{25}$ = + 5.7 (c 0.40, $CHCl_3$); ^{1}H NMR (600 MHz, $CDCl_3$) δ 5.87 (d, J = 11.6 Hz, 1H), 5.76 (dq, J = 11.5, 6.8 Hz, 1H), 5.26 (s, 1H), 4.98 (s, 1H), 4.10 (t, J = 6.4 Hz, 1H), 1.79 (d, J = 6.9 Hz, 3H), 1.49 – 1.56 (m, 3H), 1.26 – 1.42 (m, 8H), 0.87 (t, J = 6.4 Hz, 3H). ^{13}C NMR (151 MHz, $CDCl_3$) δ 147.4, 129.2, 126.8, 113.7, 76.1, 36.0, 32.1, 29.5, 25.9, 22.9, 15.2, 14.5. HRMS (EI^+) *m/z*: $[M]^+$ calcd. for $C_{12}H_{22}O$ 182.1671; found: 182.1680.

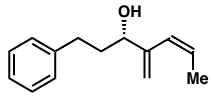

(*S*,*Z*)-4-methylene-1-phenylhept-5-en-3-ol (2r) Prepared according to the general procedure from **1a**. The crude mixture was purified by flash column chromatography (gradient elution with hexane and Et_2O, 20:1 to 5:1) to give the title compound as colorless oil in 74% yield (15 mg). The enantiomeric excess was determined to be 94% ee by Mosher ester analysis; $[\alpha]_D^{25}$ = + 6.0 (c 0.35, $CHCl_3$); ^{1}H NMR (600 MHz, $CDCl_3$) δ 7.27 – 7.29 (m, 2H), 7.18 – 7.20 (m, 3H), 5.88 (d, J = 11.7 Hz, 1H), 5.78 (dq, J = 11.6, 6.8 Hz, 1H), 5.30 (s, 1H), 5.02 (s, 1H), 4.13 – 4.15 (m, 1H), 2.69 – 2.74 (m, 1H), 2.61 – 2.66 (m, 1H), 1.83 – 1.90 (m, 2H), 1.80 (d, J = 6.7 Hz, 3H). ^{13}C NMR (151 MHz, $CDCl_3$) δ 146.9, 142.0, 129.1, 128.5, 128.4, 126.5, 125.9, 113.7, 74.9, 37.4, 31.9, 15.0. HRMS (EI^+) *m/z*: $[M–H_2O]^+$ calcd. for $C_{14}H_{16}$ 184.1252; found: 184.1257.

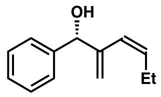

(*R*,*Z*)-2-methylene-1-phenylhex-3-en-1-ol (4a) Prepared according to the general procedure from **1b**. The crude mixture was purified by flash column chromatography (gradient elution with hexane and Et_2O, 20:1 to 5:1) to give the title compound as colorless oil in 85% yield (16 mg). The enantiomeric excess was determined to be 99% ee by Mosher ester analysis; $[\alpha]_D^{25}$ = + 8.8 (c 0.40, $CHCl_3$); ^{1}H NMR (600 MHz, $CDCl_3$) δ 7.33 – 7.38 (m, 4H), 7.26 – 7.28 (m, 1H), 5.62 (d, J = 11.7 Hz, 1H), 5.55 (dt, J = 11.7, 7.2 Hz, 1H), 5.45 (s, 1H), 5.20 (d, J = 3.7 Hz, 1H), 5.09 (s, 1H), 2.16 – 2.20 (m, 2H), 1.97 (d, J = 3.9 Hz, 1H), 0.91 (t, J = 7.5 Hz, 3H). ^{13}C

NMR (151 MHz, $CDCl_3$) δ 146.7, 142.1, 137.4, 128.7, 128.0, 126.8, 125.2, 113.9, 77.6, 22.5, 14.8. HRMS (EI^+) *m/z*: $[M]^+$ calcd. for $C_{13}H_{16}O$ 188.1201; found: 188.1197.

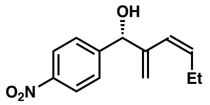

(*R*,*Z*)-2-methylene-1-(4-nitrophenyl)hex-3-en-1-ol (4b) Prepared according to the general procedure from **1b**. The crude mixture was purified by flash column chromatography (gradient elution with hexane and Et_2O, 20:1 to 2:1) to give the title compound as colorless oil in 94% yield (22 mg). The enantiomeric excess was determined to be 99% ee by Mosher ester analysis; $[\alpha]_D^{25}$ = – 2.4 (c 0.42, $CHCl_3$); ^1H NMR (600 MHz, $CDCl_3$) δ 8.20 (d, J = 8.6 Hz, 2H), 7.56 (d, J = 8.4 Hz, 2H), 5.57 – 5.63 (m, 2H), 5.46 (s, 1H), 5.31 (s, 1H), 5.13 (s, 1H), 2.11 – 2.16 (m, 3H), 0.90 (t, J = 7.5 Hz, 3H). ^{13}C NMR (151 MHz, $CDCl_3$) δ 149.4, 147.5, 145.9, 138.6, 127.4, 124.0, 123.8, 115.6, 77.1, 22.5, 14.7. HRMS (EI^+) *m/z*: $[M]^+$ calcd. for $C_{13}H_{15}NO_3$ 233.1052; found: 233.1060.

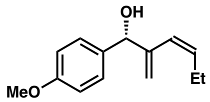

(*R*,*Z*)-1-(4-methoxyphenyl)-2-methylenehex-3-en-1-ol (4c) Prepared according to the general procedure from **1b**. The crude mixture was purified by flash column chromatography (gradient elution with hexane and Et_2O, 20:1 to 2:1) to give the title compound as colorless oil in 73% yield (16 mg). The enantiomeric excess was determined to be 98% ee by Mosher ester analysis; $[\alpha]_D^{25}$ = + 52.5 (c 0.40, $CHCl_3$); ^1H NMR (600 MHz, $CDCl_3$) δ 7.29 (d, J = 8.4 Hz, 2H), 6.87 (d, J = 8.6 Hz, 2H), 5.61 (d, J = 11.7 Hz, 1H), 5.53 (dt, J = 11.6, 7.1 Hz, 1H), 5.45 (s, 1H), 5.15 (d, J = 3.8 Hz, 1H), 5.09 (s, 1H), 3.80 (s, 3H), 2.16 – 2.21 (m, 2H), 1.91 (d, J = 3.9 Hz, 1H), 0.92 (t, J = 7.5 Hz, 3H). ^{13}C NMR (151 MHz, $CDCl_3$) δ 159.3, 146.8, 137.1, 134.3, 128.2, 125.5, 114.0, 113.5, 77.0, 55.6, 22.6, 14.8. HRMS (EI^+) *m/z*: $[M]^+$ calcd. for $C_{14}H_{18}O_2$ 218.1307; found: 218.1296.

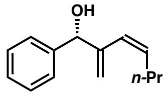

(*R*,*Z*)-2-methylene-1-phenylhept-3-en-1-ol (4d) Prepared according to the general procedure from **1c**. The crude mixture was purified (gradient elution with hexane and Et_2O, 20:1 to 5:1) to give the title compound as colorless oil in 84% yield (17 mg). The enantiomeric excess was determined to be 99% ee by Mosher ester analysis; $[\alpha]_D^{25}$ = + 16.4 (c 0.55, $CHCl_3$); ^1H NMR (600 MHz, $CDCl_3$) δ 7.32 – 7.38 (m, 4H), 7.28 – 7.27 (m, 1H), 5.66 (d, J = 11.8 Hz, 1H), 5.56 (dt, J = 11.7, 7.2 Hz, 1H), 5.46 (s, 1H), 5.20 (d, J = 3.9 Hz, 1H), 5.10 (s, 1H), 2.12 – 2.16 (m, 2H), 1.98 (d, J = 4.0 Hz, 1H), 1.27 – 1.36 (m, 2H), 0.83 (t, J = 7.4 Hz, 3H). ^{13}C NMR (151 MHz, $CDCl_3$) δ 146.7, 142.1, 135.8, 128.6, 127.9, 126.8, 125.8, 113.9, 77.6, 31.2, 23.4, 14.2. HRMS (EI^+) *m/z*: $[M]^+$ calcd. for $C_{14}H_{18}O$ 202.1358; found: 202.1353.

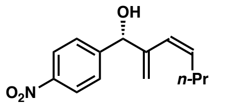

(*R*,*Z*)-2-methylene-1-(4-nitrophenyl)hept-3-en-1-ol (4e) Prepared according to the general procedure from **1c**. The crude mixture was purified by flash column chromatography (gradient elution with hexane and Et_2O, 20:1 to 2:1) to give the title compound as colorless oil in 89% yield (22 mg). The enantiomeric excess was determined to be 99% ee by Mosher ester analysis; $[\alpha]_D^{25}$ = – 5.7 (c 0.60, $CHCl_3$); ^{1}H NMR (600 MHz, $CDCl_3$) δ 8.19 (d, *J* = 8.7 Hz, 2H), 7.56 (d, *J* = 8.3 Hz, 2H), 5.59 – 5.63 (m, 2H), 5.46 (s, 1H), 5.31 (d, *J* = 3.6 Hz, 1H), 5.13 (s, 1H), 2.08 – 2.13 (m, 3H), 1.28 – 1.34 (m, 2H), 0.82 (t, *J* = 7.4 Hz, 3H). ^{13}C NMR (151 MHz, $CDCl_3$) δ 149.3, 147.5, 145.9, 137.0, 127.4, 124.7, 123.8, 115.6, 77.1, 31.1, 23.3, 14.1. HRMS (EI$^+$) *m/z*: [M]$^+$ calcd. for $C_{14}H_{17}NO_3$ 247.1208; found: 247.1207.

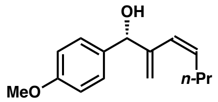

(*R*,*Z*)-1-(4-methoxyphenyl)-2-methylenehept-3-en-1-ol (4f) Prepared according to the general procedure from **1c**. The crude mixture was purified by flash column chromatography (gradient elution with hexane and Et_2O, 20:1 to 2:1) to give the title compound as colorless oil in 77% yield (18 mg). The enantiomeric excess was determined to be 98% ee by Mosher ester analysis; $[\alpha]_D^{25}$ = + 51.0 (c 0.35, $CHCl_3$); ^{1}H NMR (600 MHz, Chloroform-d) δ 7.28 (d, *J* = 8.4 Hz, 2H), 6.86 (d, *J* = 8.5 Hz, 2H), 5.64 (d, *J* = 11.7 Hz, 1H), 5.54 (dt, *J* = 11.6, 7.2 Hz, 1H), 5.45 (s, 1H), 5.14 (d, *J* = 3.8 Hz, 1H), 5.09 (s, 1H), 3.80 (s, 3H), 2.13 – 2.17 (m, 2H), 1.91 (d, *J* = 3.9 Hz, 1H), 1.30 – 1.34 (m, 2H), 0.85 (t, *J* = 7.4 Hz, 3H). ^{13}C NMR (151 MHz, $CDCl_3$) δ 159.3, 146.9, 135.5, 134.3, 128.2, 126.1, 114.0, 113.5, 77.1, 55.6, 31.2, 23.4, 14.2. HRMS (EI$^+$) *m/z*: [M]$^+$ calcd. for $C_{15}H_{20}O_2$ 232.1463; found: 232.1459.

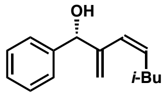

(*R*,*Z*)-6-methyl-2-methylene-1-phenylhept-3-en-1-ol (4g) Prepared according to the general procedure from **1d**. The crude mixture was purified by flash column chromatography (gradient elution with hexane and Et_2O, 20:1 to 5:1) to give the title compound as colorless oil in 83% yield (18 mg). The enantiomeric excess was determined to be 99% ee by Mosher ester analysis; $[\alpha]_D^{25}$ = + 51.2 (c 0.60, $CHCl_3$); ^{1}H NMR (600 MHz, $CDCl_3$) δ 7.32 – 7.37 (m, 4H), 7.27 – 7.28 (m, 1H), 5.69 (d, *J* = 11.8 Hz, 1H), 5.57 (dt, *J* = 11.8, 7.2 Hz, 1H), 5.46 (s, 1H), 5.19 (d, *J* = 3.9 Hz, 1H), 5.09 (s, 1H), 2.04 – 2.07 (m, 2H), 1.98 (d, *J* = 4.0 Hz, 1H), 1.51 – 1.58 (m, 1H), 0.83 (d, *J* = 6.7 Hz, 3H), 0.80 (d, *J* = 6.7 Hz, 3H). ^{13}C NMR (151 MHz, $CDCl_3$) δ 146.7, 142.1, 134.8, 128.6, 127.9, 126.8, 126.3, 114.0, 77.6, 38.1, 29.2, 22.8, 22.7. HRMS (EI$^+$) *m/z*: [M]$^+$ calcd. for $C_{15}H_{20}O$ 216.1514; found: 216.1523.

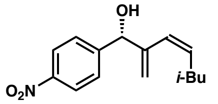

(*R*,*Z*)-6-methyl-2-methylene-1-(4-nitrophenyl)hept-3-en-1-ol (4h) Prepared according to the general procedure from **1d**. The crude mixture was purified by flash column chromatography (gradient elution with hexane and Et_2O, 20:1 to 2:1) to give the title compound as colorless oil in 84% yield (22 mg). The enantiomeric excess was determined to be 99% ee by Mosher ester analysis; $[\alpha]_D^{25}$ = – 4.7 (c 0.80, $CHCl_3$); ^1H NMR (600 MHz, $CDCl_3$) δ 8.19 (d, *J* = 8.7 Hz, 2H), 7.55 (d, *J* = 8.4 Hz, 2H), 5.60 – 5.67 (m, 2H), 5.46 (s, 1H), 5.31 (d, *J* = 3.5 Hz, 1H), 5.13 (s, 1H), 2.13 (d, *J* = 3.6 Hz, 1H), 2.02 (dd, *J* = 6.5, 6.5 Hz, 2H), 1.52 – 1.56 (m, 1H), 0.802 (d, *J* = 6.8 Hz, 3H), 0.797 (d, *J* = 6.7 Hz, 3H). ^{13}C NMR (151 MHz, $CDCl_3$) δ 149.3, 147.5, 145.9, 136.1, 127.4, 125.1, 123.8, 115.7, 77.1, 38.0, 29.1, 22.7, 22.6. HRMS (EI^+) *m/z*: $[M]^+$ calcd. for $C_{15}H_{19}NO_3$ 261.1365; found: 261.1357.

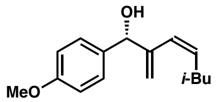

(*R*,*Z*)-1-(4-methoxyphenyl)-6-methyl-2-methylenehept-3-en-1-ol (4i) Prepared according to the general procedure from **1d**. The crude mixture was purified by flash column chromatography (gradient elution with hexane and Et_2O, 20:1 to 2:1) to give the title compound as colorless oil in 73% yield (18 mg). The enantiomeric excess was determined to be 97% ee by Mosher ester analysis; $[\alpha]_D^{25}$ = + 56.2 (c 0.30, $CHCl_3$); ^1H NMR (600 MHz, $CDCl_3$) δ 7.28 (d, *J* = 8.2 Hz, 2H), 6.86 (d, *J* = 8.6 Hz, 2H), 5.67 (d, *J* = 11.8 Hz, 1H), 5.56 (dt, *J* = 11.8, 7.1 Hz, 1H), 5.45 (s, 1H), 5.14 (d, *J* = 3.8 Hz, 1H), 5.08 (s, 1H), 3.80 (s, 3H), 2.07 (dd, *J* = 6.7, 6.7 Hz, 2H), 1.91 (d, *J* = 3.9 Hz, 1H), 1.52 – 1.58 (m, 1H), 0.85 (d, *J* = 6.6 Hz, 3H), 0.82 (d, *J* = 6.7 Hz, 3H). ^{13}C NMR (151 MHz, $CDCl_3$) δ 159.3, 146.9, 134.5, 134.3, 128.2, 126.5, 114.0, 113.5, 77.1, 55.6, 38.1, 29.2, 22.8, 22.7. HRMS (EI^+) *m/z*: $[M]^+$ calcd. for $C_{16}H_{22}O_2$ 246.1620; found: 246.1609.

References:

1. Hopf, H.; Böhm, I.; Kleinschroth, *Org. Syn.* **1981**, *60*, 41.

2. (a) Dale, J. A.; Mosher, H. S. *J. Am. Chem. Soc.* **1973**, *95*, 512. (b) Ohtani, I.; Kusumi, T.; Kashman, Y.; Kakisawa, H. *J. Am. Chem. Soc.* **1991**, *113*, 4092. (c) Hoye, T. R.; Jeffrey, C. S.; Shao, F. *Nat. Protoc.* **2007**, *2,* 2451.

Assignment of the absolute configuration using Mosher ester analysis: [2]

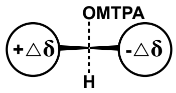

$$\triangle\delta = \delta_{S\text{-ester}} - \delta_{R\text{-ester}}$$

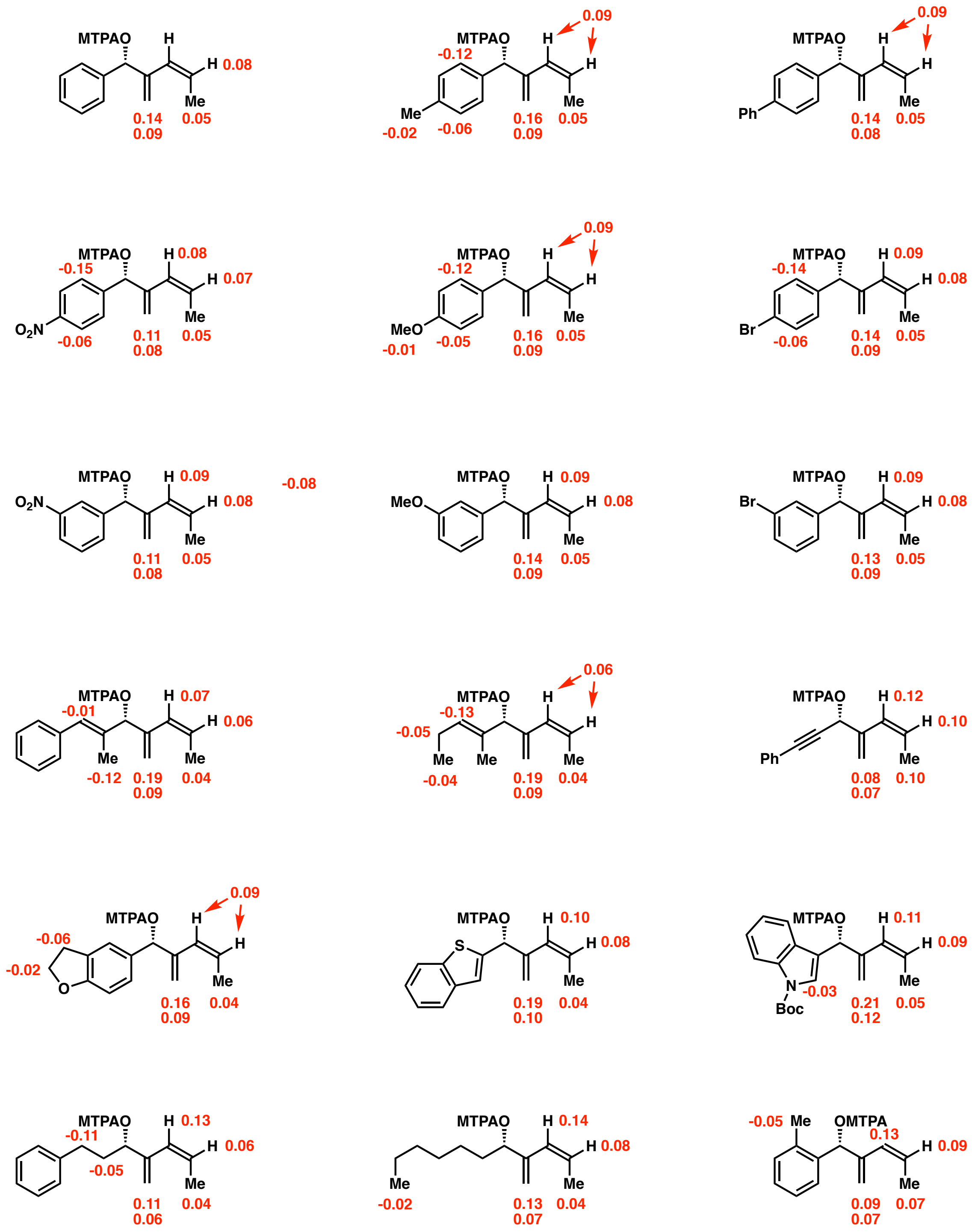

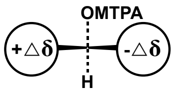

$$\triangle\delta = \delta_{S\text{-ester}} - \delta_{R\text{-ester}}$$

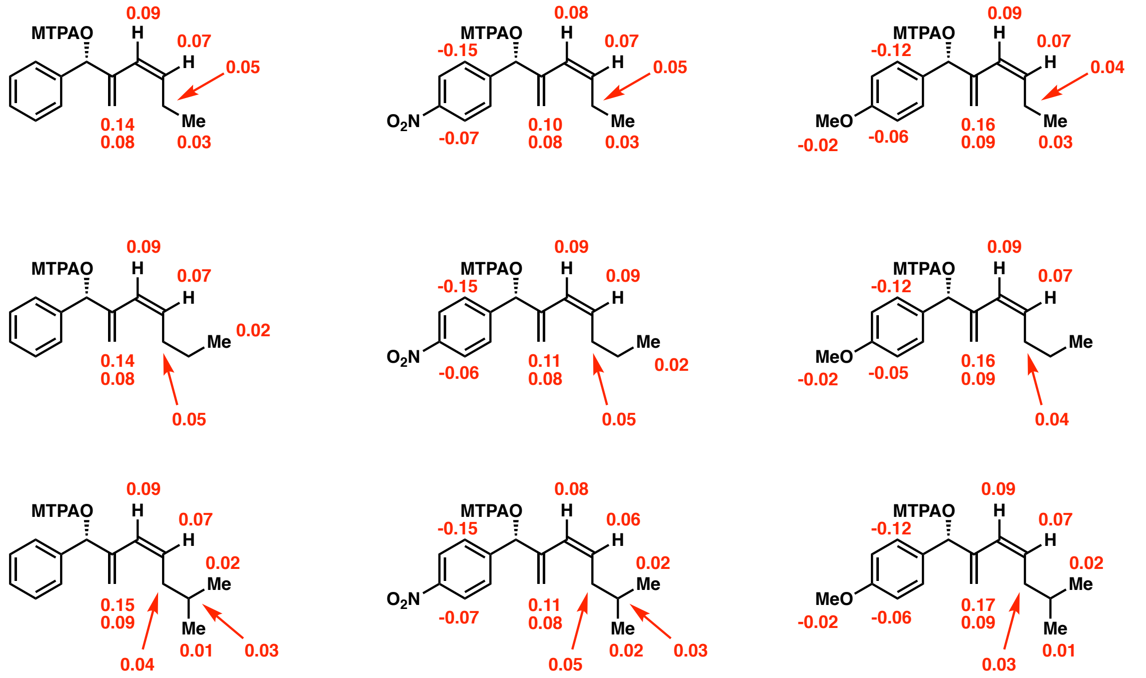

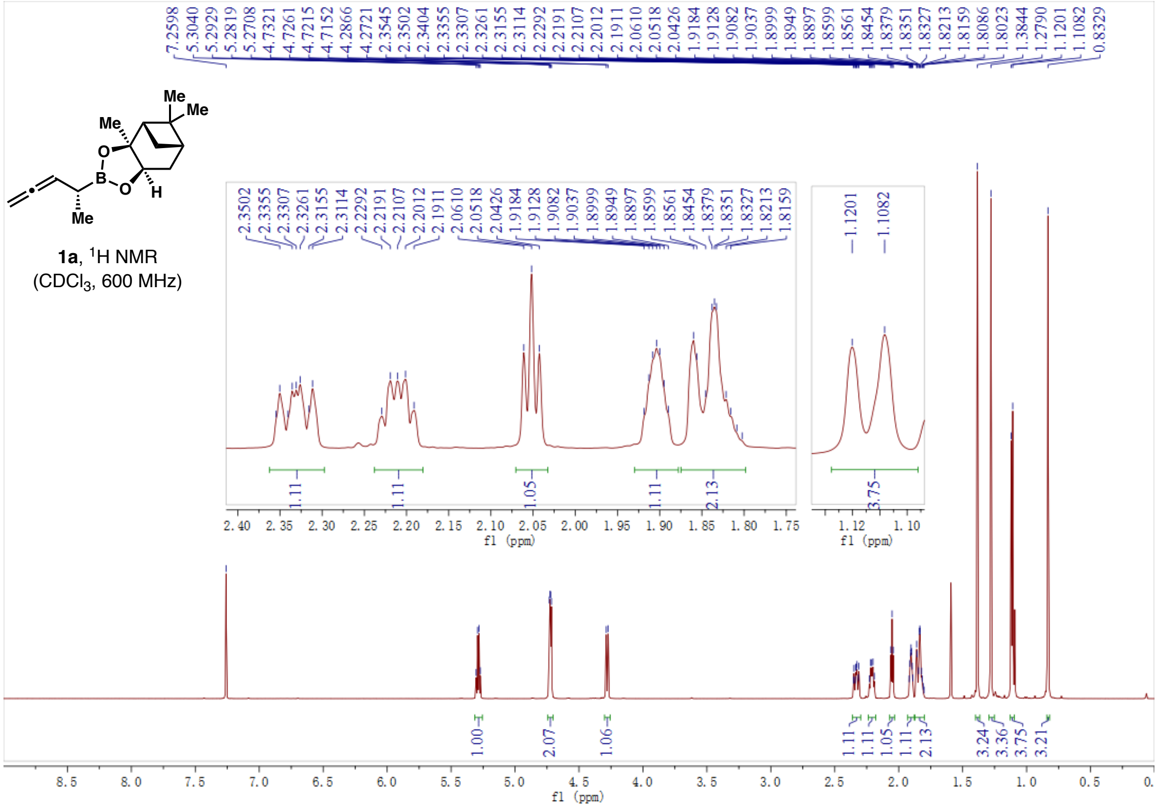
7.2598
5.3040
5.2929
5.2819
5.2708
4.7321
4.7261
4.7215
4.7152
4.2866
4.2721
2.3545
2.3502
2.3404
2.3355
2.3307
2.3261
2.3155
2.3114
2.2292
2.2191
2.2107
2.2012
2.1911
2.0610
2.0518
2.0426
1.9184
1.9128
1.9082
1.9037
1.8999
1.8949
1.8897
1.8599
1.8561
1.8454
1.8379
1.8351
1.8327
1.8213
1.8159
1.8086
1.8023
1.3844
1.2790
1.1201
1.1082
0.8329
Me
Me
Me
O
B
O
H
Me
1a, ^{1}H NMR
($CDCl_3$, 600 MHz)
1.11
1.11
1.05
1.11
2.13
3.75
1.00
2.07
1.06
1.11
1.11
1.05
1.11
2.13
3.24
3.36
3.75
3.21
f1 (ppm)

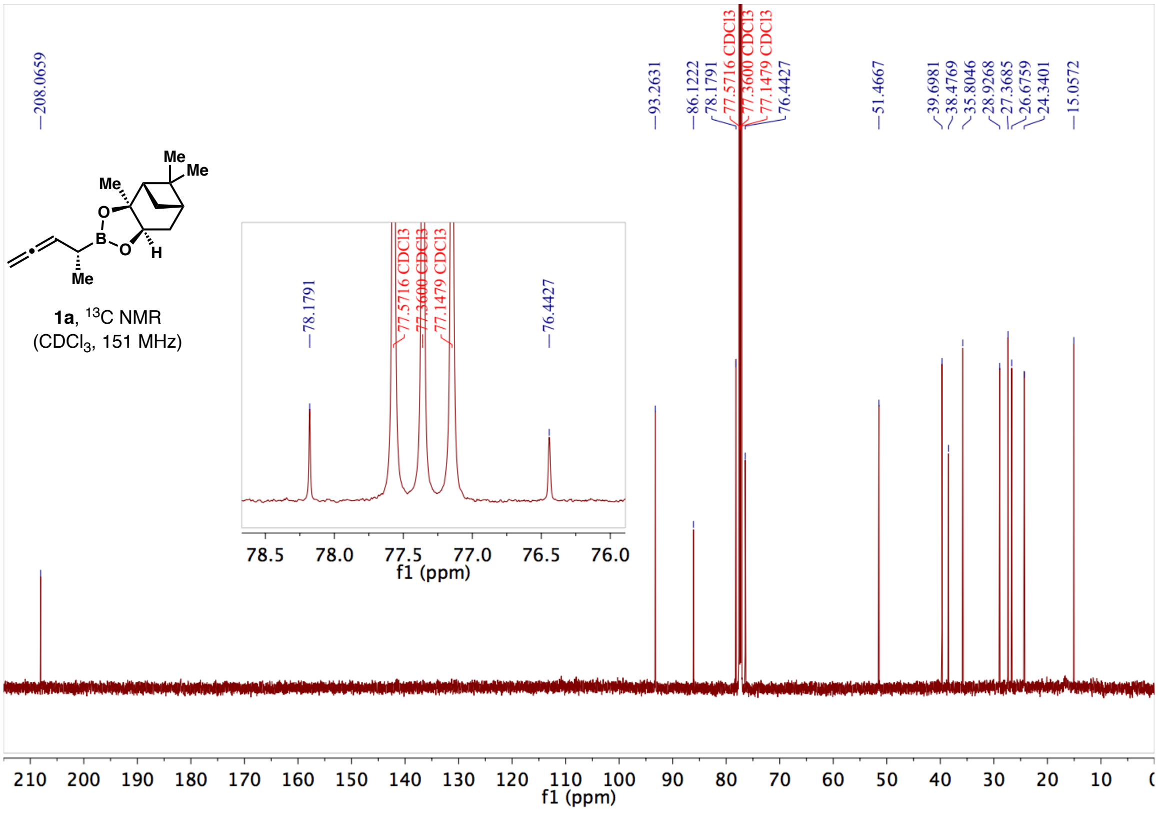
208.0659
93.2631
86.1222
78.1791
77.5716 CDCl3
77.3600 CDCl3
77.1479 CDCl3
76.4427
51.4667
39.6981
38.4769
35.8046
28.9268
27.3685
26.6759
24.3401
15.0572
Me
Me
Me
O
B
O
H
Me
1a, ^{13}C NMR
($CDCl_3$, 151 MHz)
f1 (ppm)

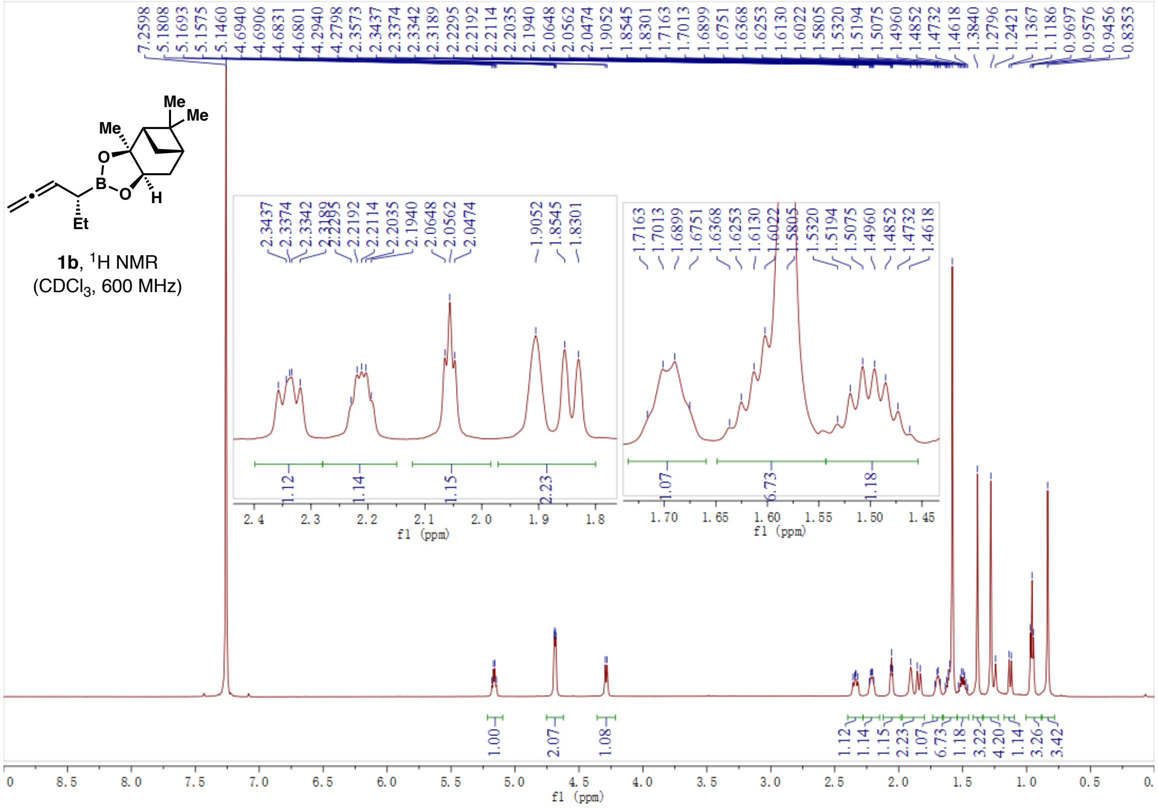

1b, ^{1}H NMR
($CDCl_3$, 600 MHz)

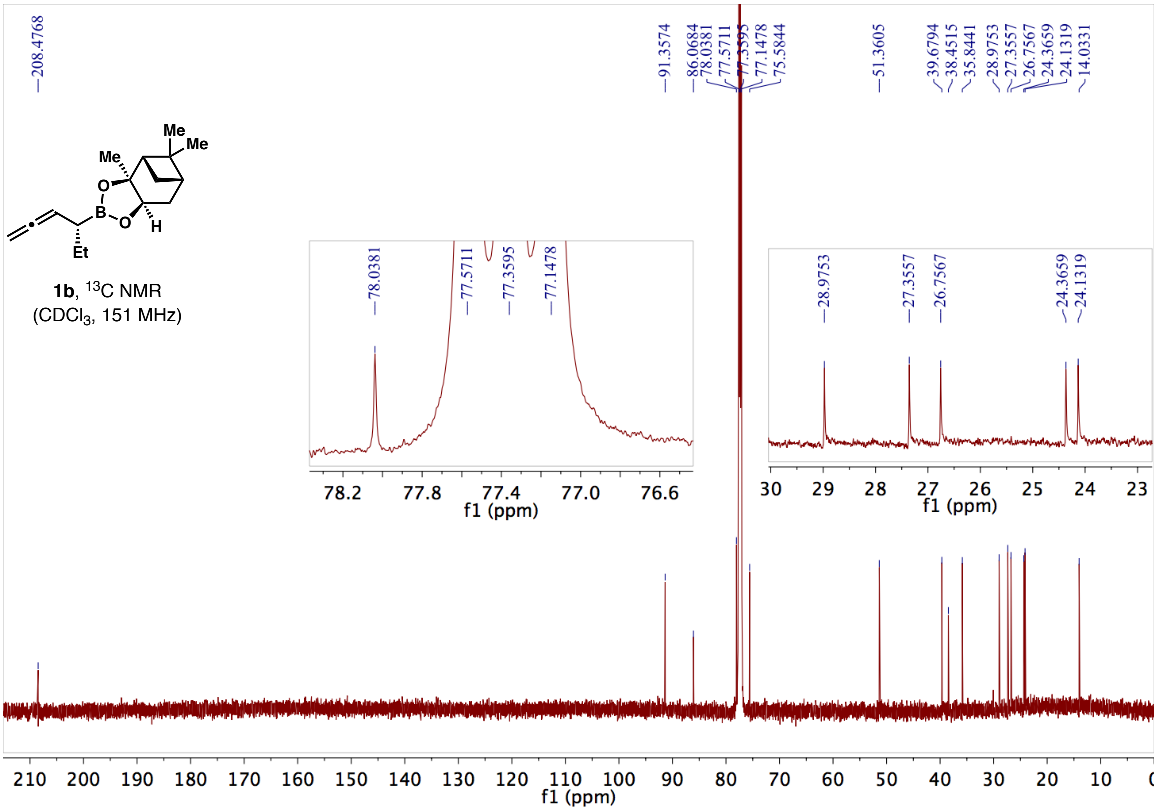

1b, ^{13}C NMR
($CDCl_3$, 151 MHz)

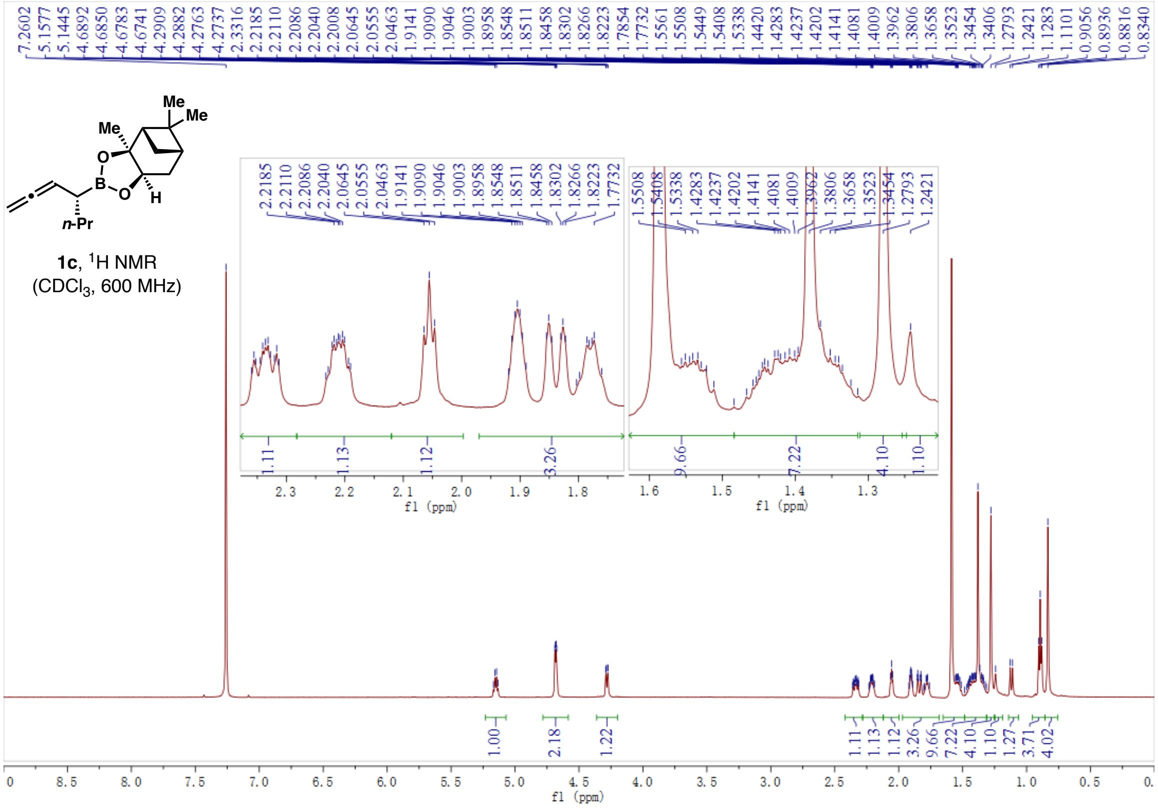

1c, ^{1}H NMR
(CDCl$_3$, 600 MHz)

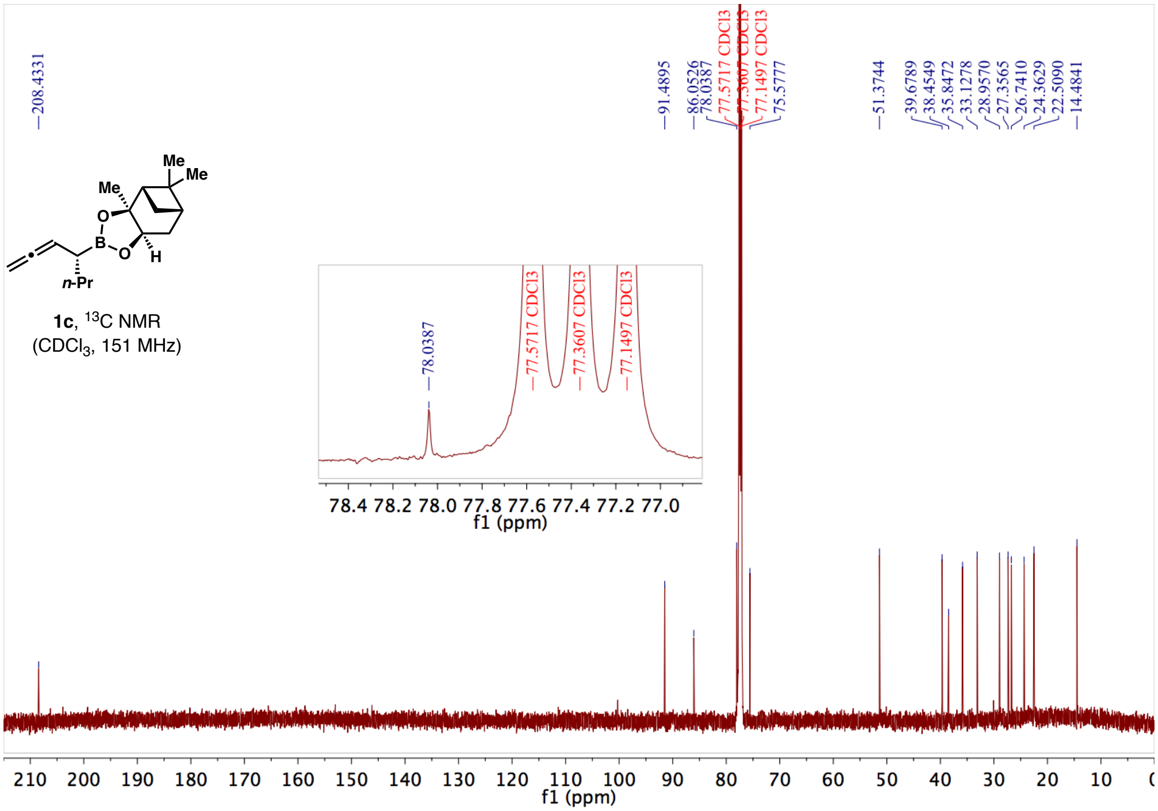

1c, ^{13}C NMR
(CDCl$_3$, 151 MHz)

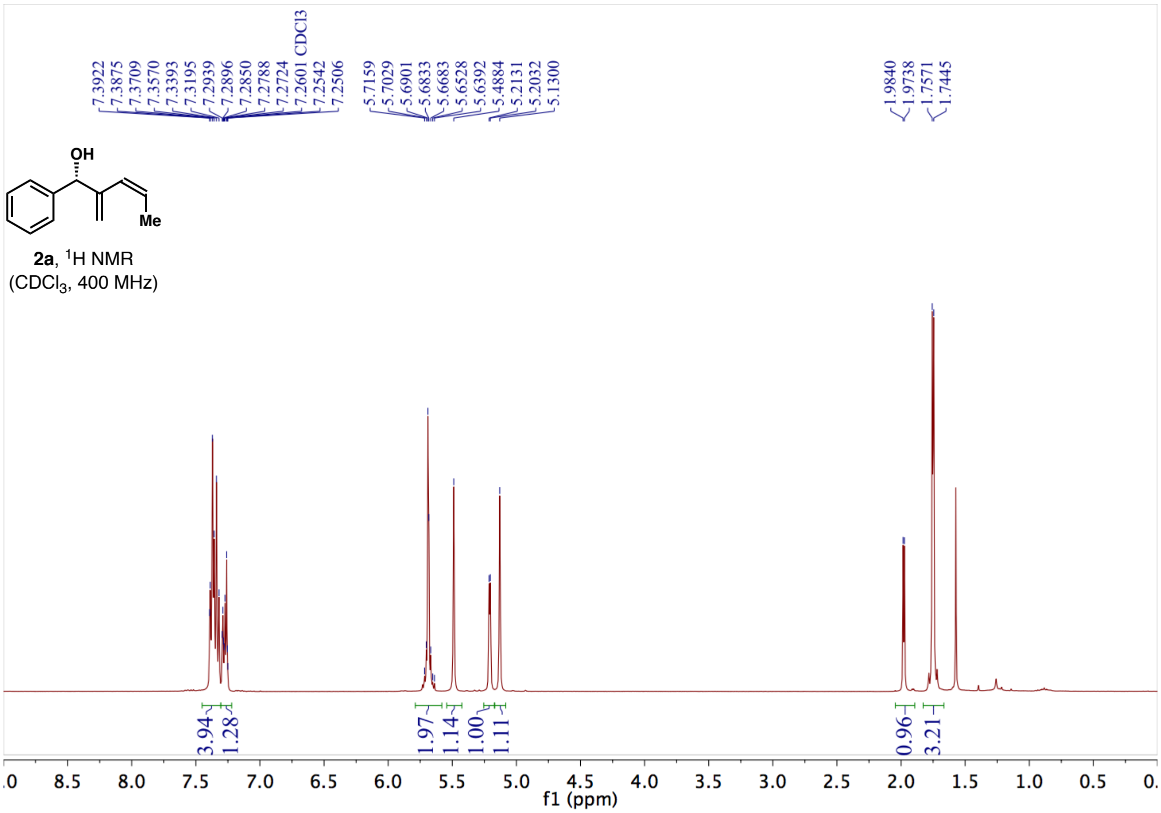
7.3922
7.3875
7.3709
7.3570
7.3393
7.3195
7.2939
7.2896
7.2850
7.2788
7.2724
7.2601 CDCl3
7.2542
7.2506
5.7159
5.7029
5.6901
5.6833
5.6683
5.6528
5.6392
5.4884
5.2131
5.2032
5.1300
1.9840
1.9738
1.7571
1.7445
OH
Me
2a, 1H NMR
(CDCl3, 400 MHz)
3.94
1.28
1.97
1.14
1.00
1.11
0.96
3.21
f1 (ppm)

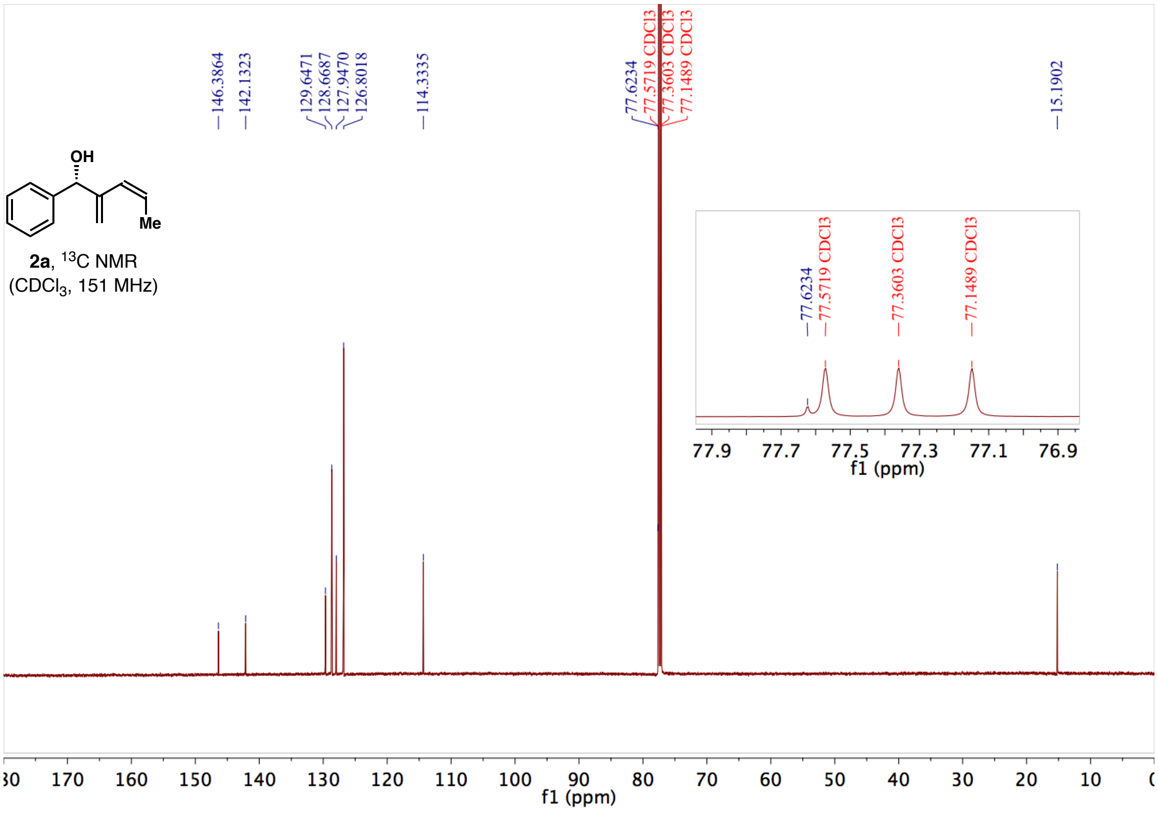
146.3864
142.1323
129.6471
128.6687
127.9470
126.8018
114.3335
77.6234
77.5719 CDCl3
77.3603 CDCl3
77.1489 CDCl3
15.1902
OH
Me
2a, 13C NMR
(CDCl3, 151 MHz)
f1 (ppm)

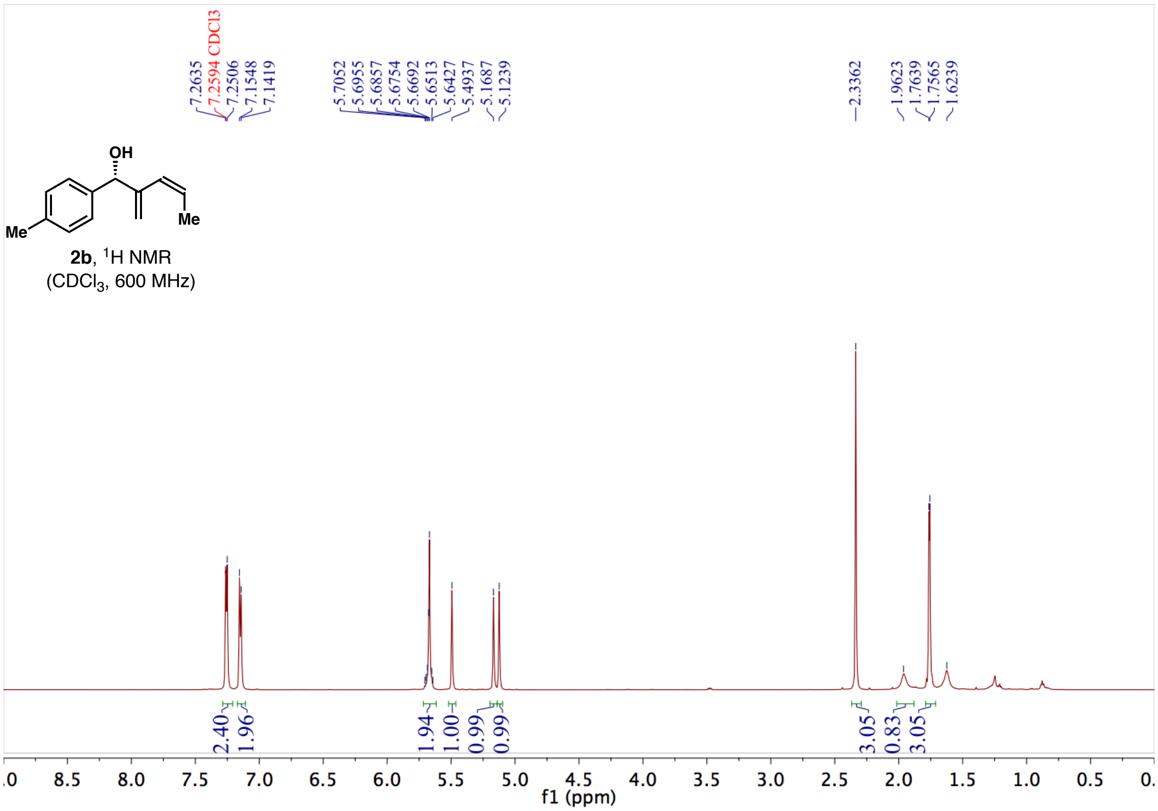
7.2635
7.2594 CDCl3
7.2506
7.1548
7.1419
5.7052
5.6955
5.6857
5.6754
5.6692
5.6513
5.6427
5.4937
5.1687
5.1239
2.3362
1.9623
1.7639
1.7565
1.6239
OH
Me
Me
2b, 1H NMR
(CDCl3, 600 MHz)
2.40
1.96
1.94
1.00
0.99
0.99
3.05
0.83
3.05
f1 (ppm)

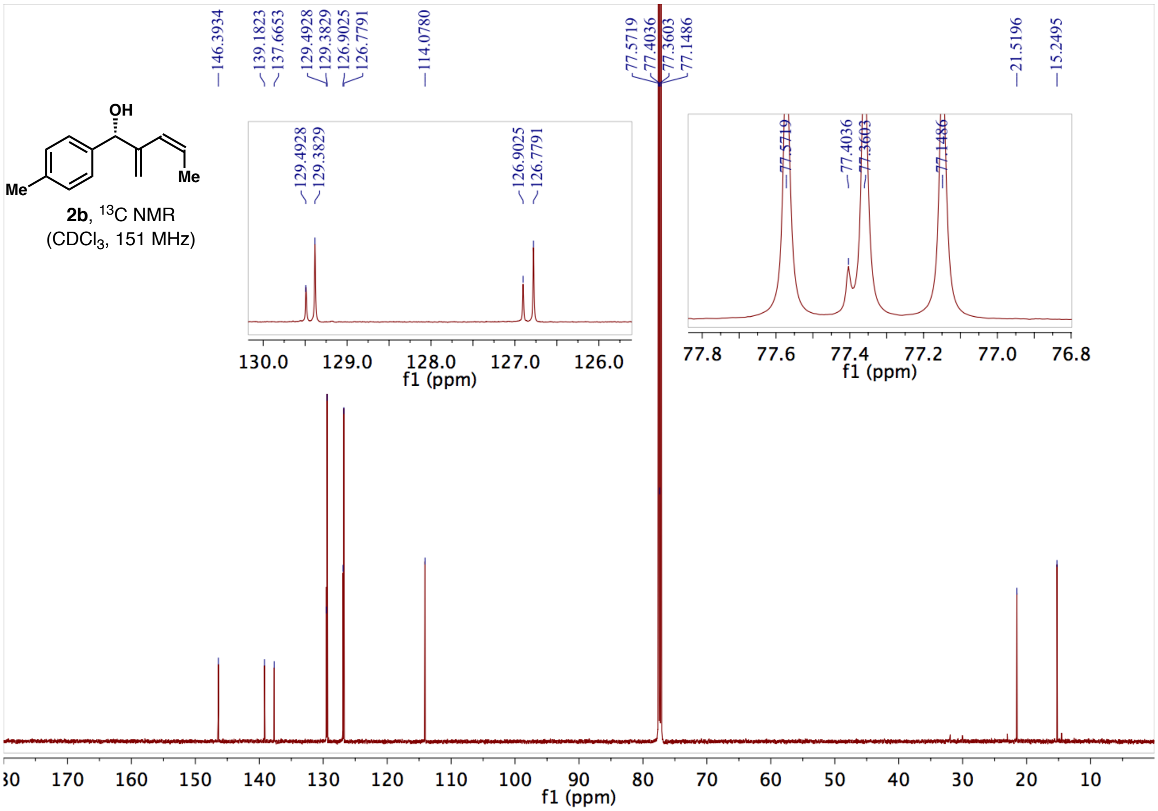
146.3934
139.1823
137.6653
129.4928
129.3829
126.9025
126.7791
114.0780
77.5719
77.4036
77.3603
77.1486
21.5196
15.2495
OH
Me
Me
2b, 13C NMR
(CDCl3, 151 MHz)
f1 (ppm)

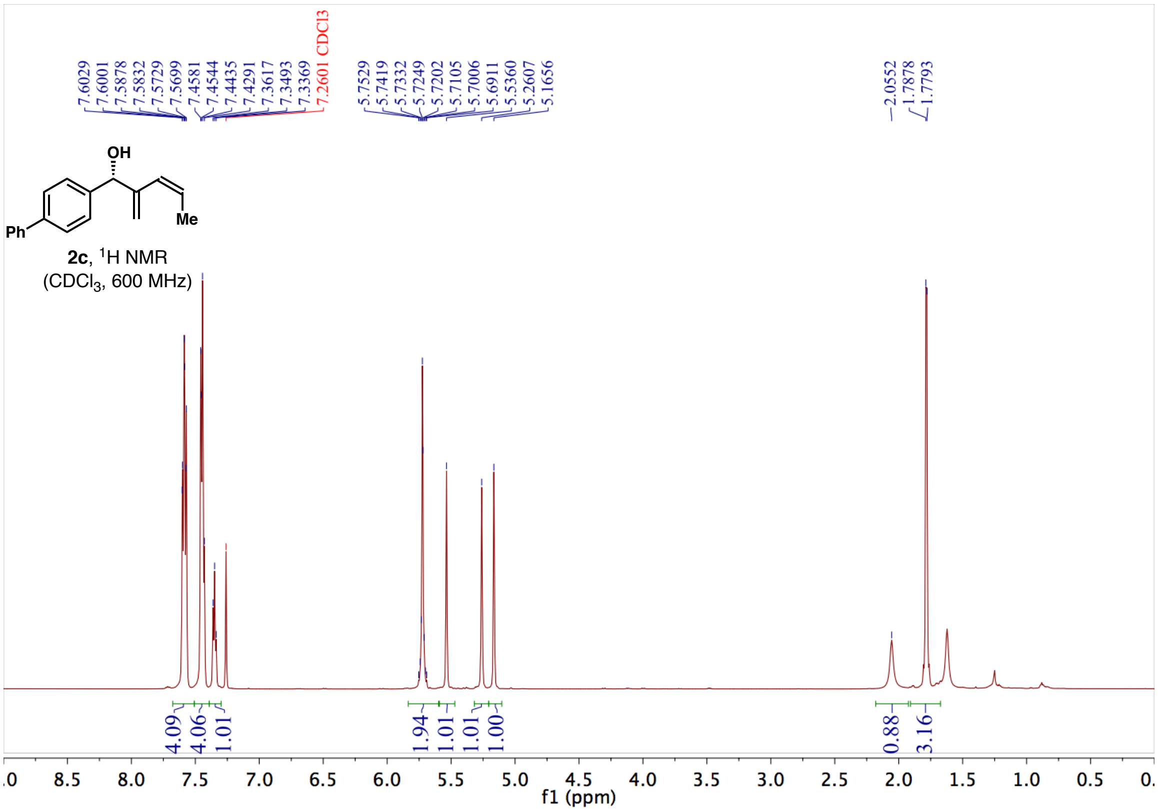
7.6029
7.6001
7.5878
7.5832
7.5729
7.5699
7.4581
7.4544
7.4435
7.4291
7.3617
7.3493
7.3369
7.2601 CDCl3
5.7529
5.7419
5.7332
5.7249
5.7202
5.7105
5.7006
5.6911
5.5360
5.2607
5.1656
2.0552
1.7878
1.7793
OH
Ph
Me
2c, 1H NMR
(CDCl3, 600 MHz)
4.09
4.06
1.01
1.94
1.01
1.01
1.00
0.88
3.16
f1 (ppm)

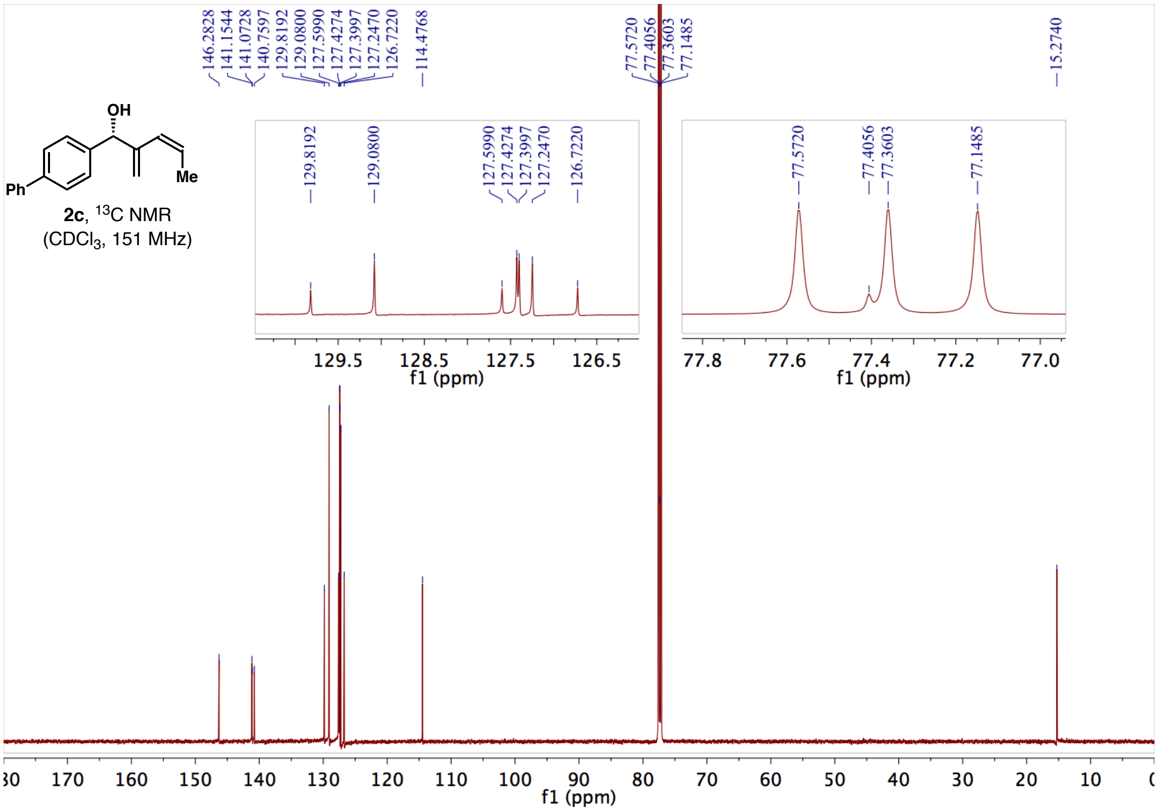
146.2828
141.1544
141.0728
140.7597
129.8192
129.0800
127.5990
127.4274
127.3997
127.2470
126.7220
114.4768
77.5720
77.4056
77.3603
77.1485
15.2740
OH
Ph
Me
2c, 13C NMR
(CDCl3, 151 MHz)
f1 (ppm)

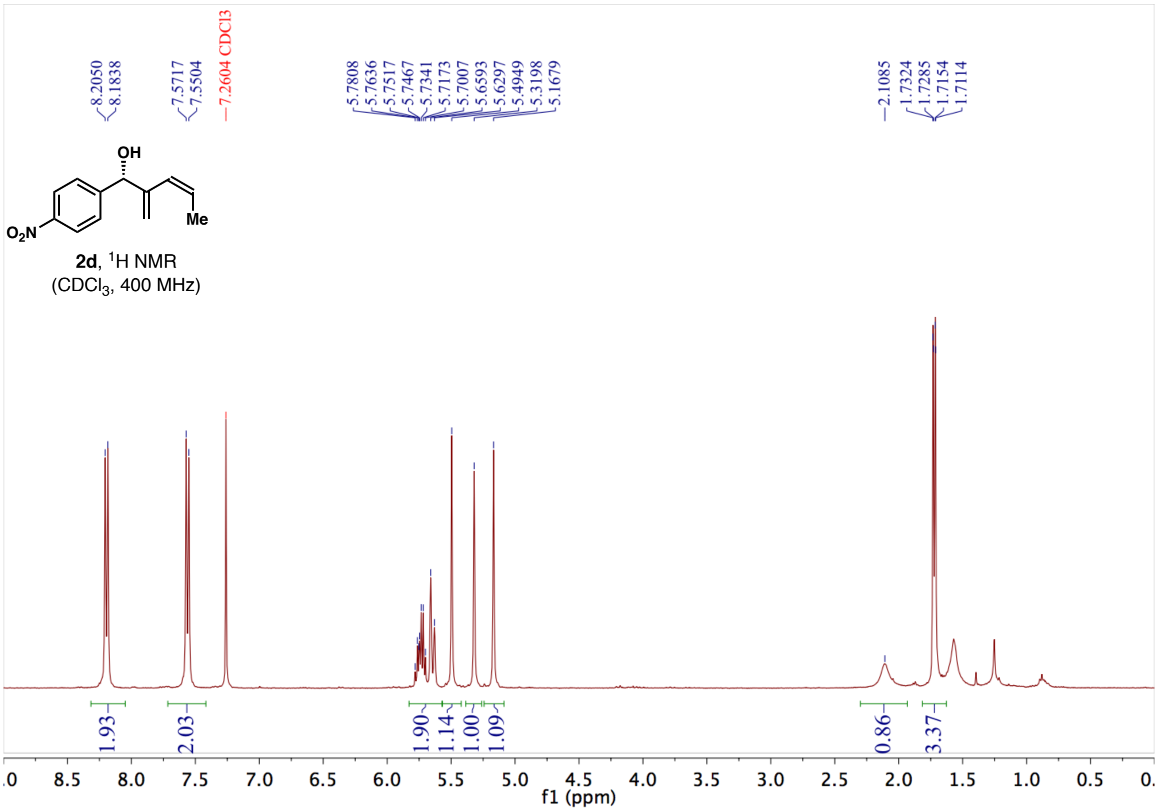
8.2050
8.1838
7.5717
7.5504
7.2604 CDCl3
5.7808
5.7636
5.7517
5.7467
5.7341
5.7173
5.7007
5.6593
5.6297
5.4949
5.3198
5.1679
2.1085
1.7324
1.7285
1.7154
1.7114
OH
O2N
Me
2d, 1H NMR
(CDCl3, 400 MHz)
1.93
2.03
1.90
1.14
1.00
1.09
0.86
3.37
f1 (ppm)

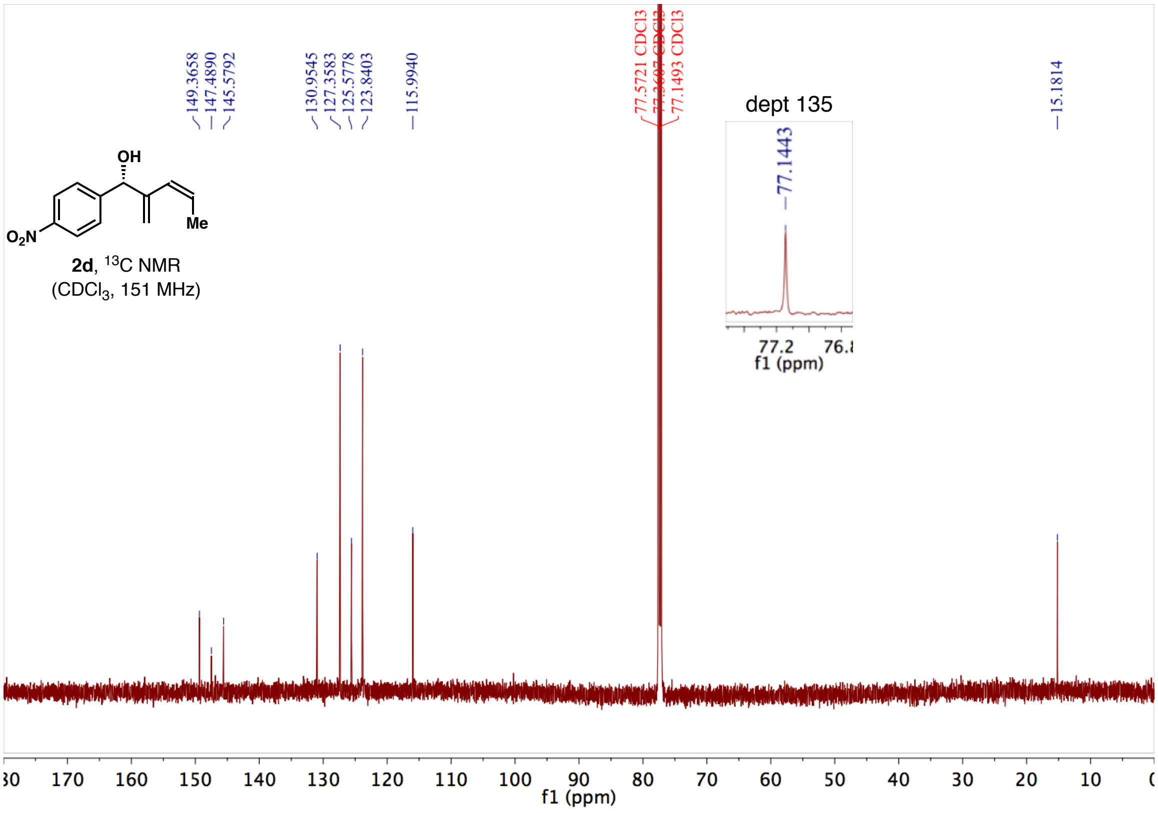
149.3658
147.4890
145.5792
130.9545
127.3583
125.5778
123.8403
115.9940
77.5721 CDCl3
77.1493 CDCl3
15.1814
dept 135
77.1443
77.2
f1 (ppm)
OH
O2N
Me
2d, 13C NMR
(CDCl3, 151 MHz)
f1 (ppm)

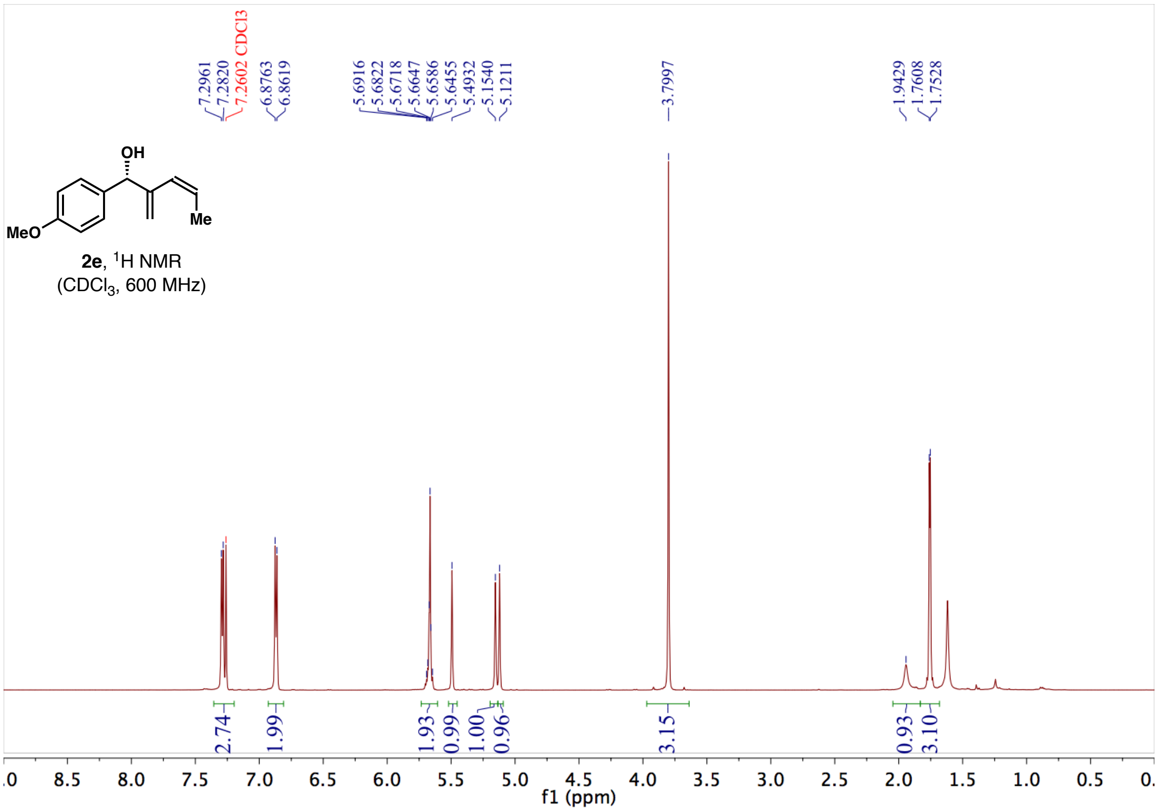
7.2961
7.2820
7.2602 CDCl3
6.8763
6.8619
5.6916
5.6822
5.6718
5.6647
5.6586
5.6455
5.4932
5.1540
5.1211
3.7997
1.9429
1.7608
1.7528
OH
MeO
Me
2e, 1H NMR
(CDCl3, 600 MHz)
2.74
1.99
1.93
0.99
1.00
0.96
3.15
0.93
3.10
f1 (ppm)

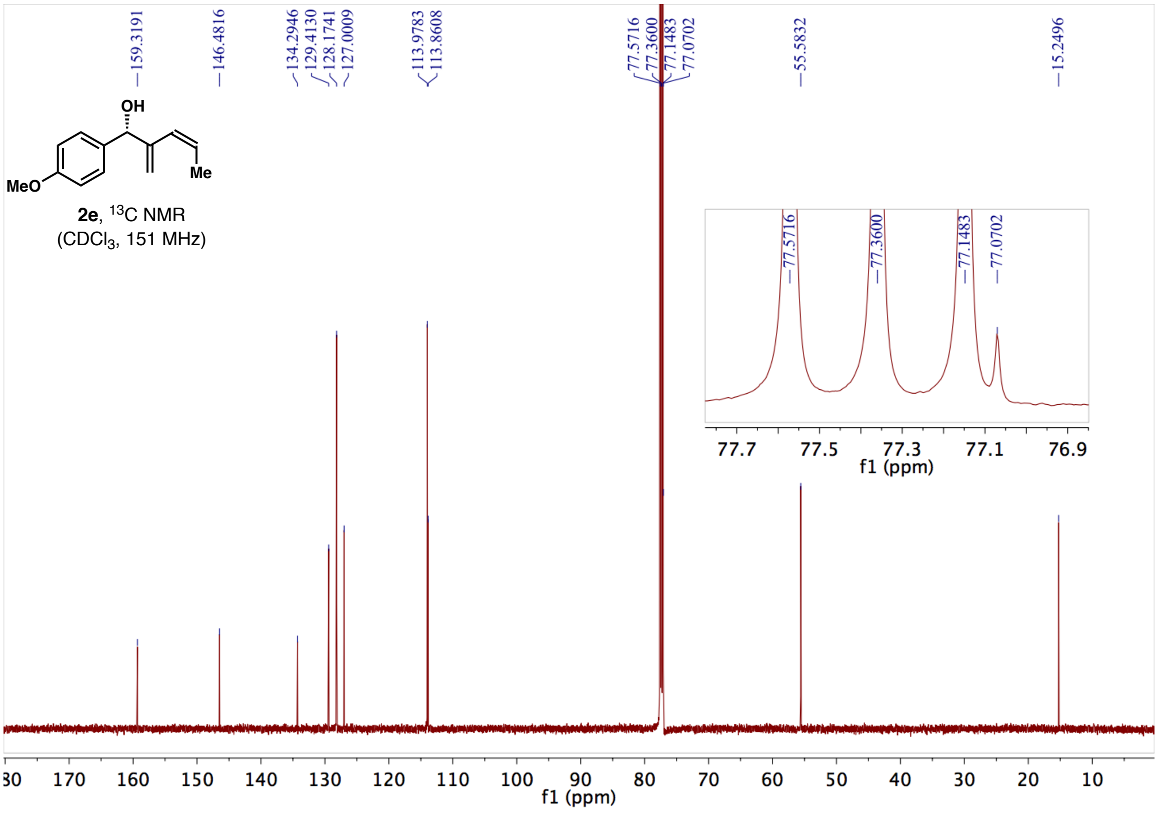
159.3191
146.4816
134.2946
129.4130
128.1741
127.0009
113.9783
113.8608
77.5716
77.3600
77.1483
77.0702
55.5832
15.2496
OH
MeO
Me
2e, 13C NMR
(CDCl3, 151 MHz)
f1 (ppm)

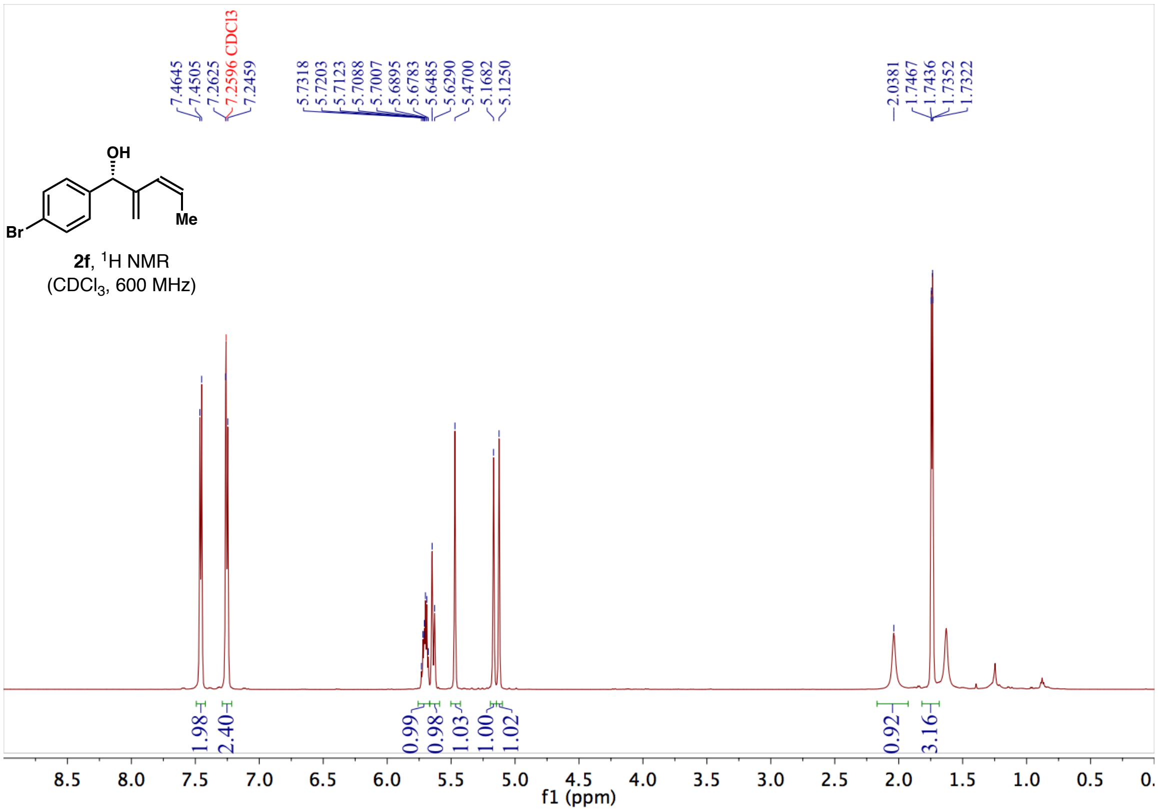
2f, ¹H NMR
(CDCl3, 600 MHz)
f1 (ppm)

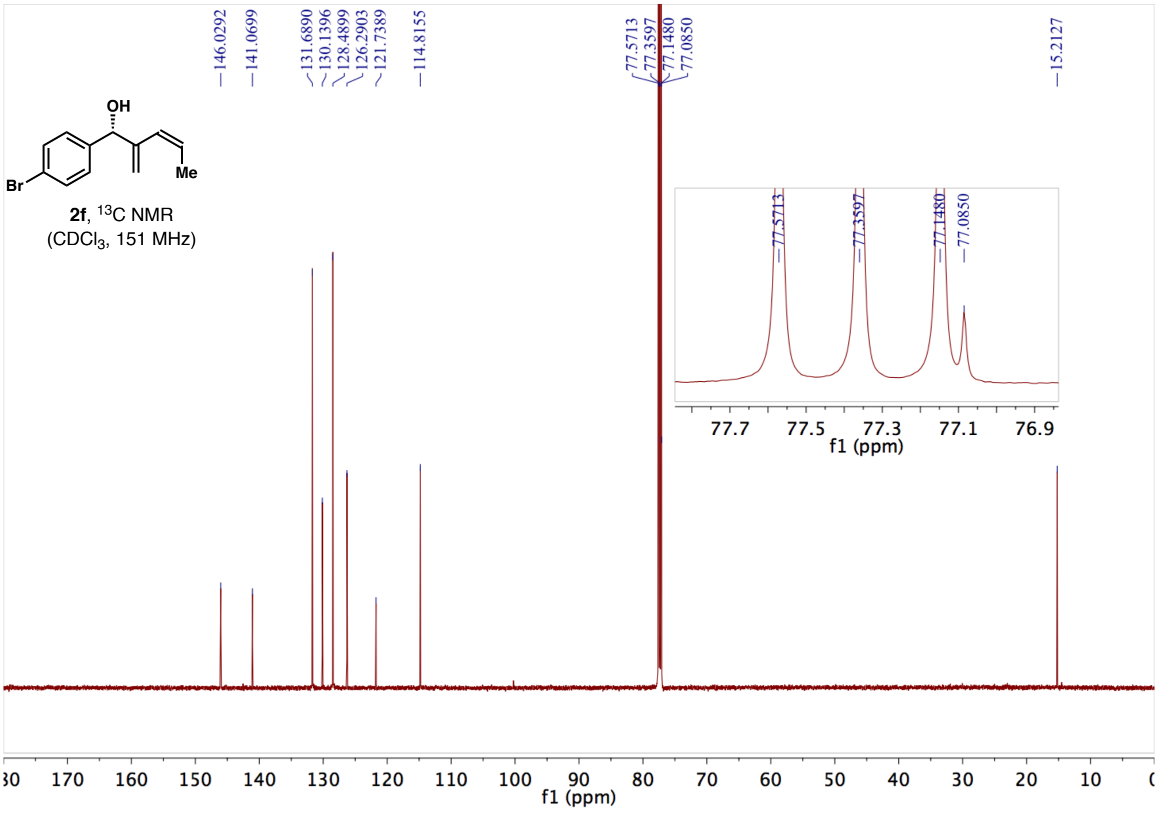
2f, ¹³C NMR
(CDCl3, 151 MHz)
f1 (ppm)

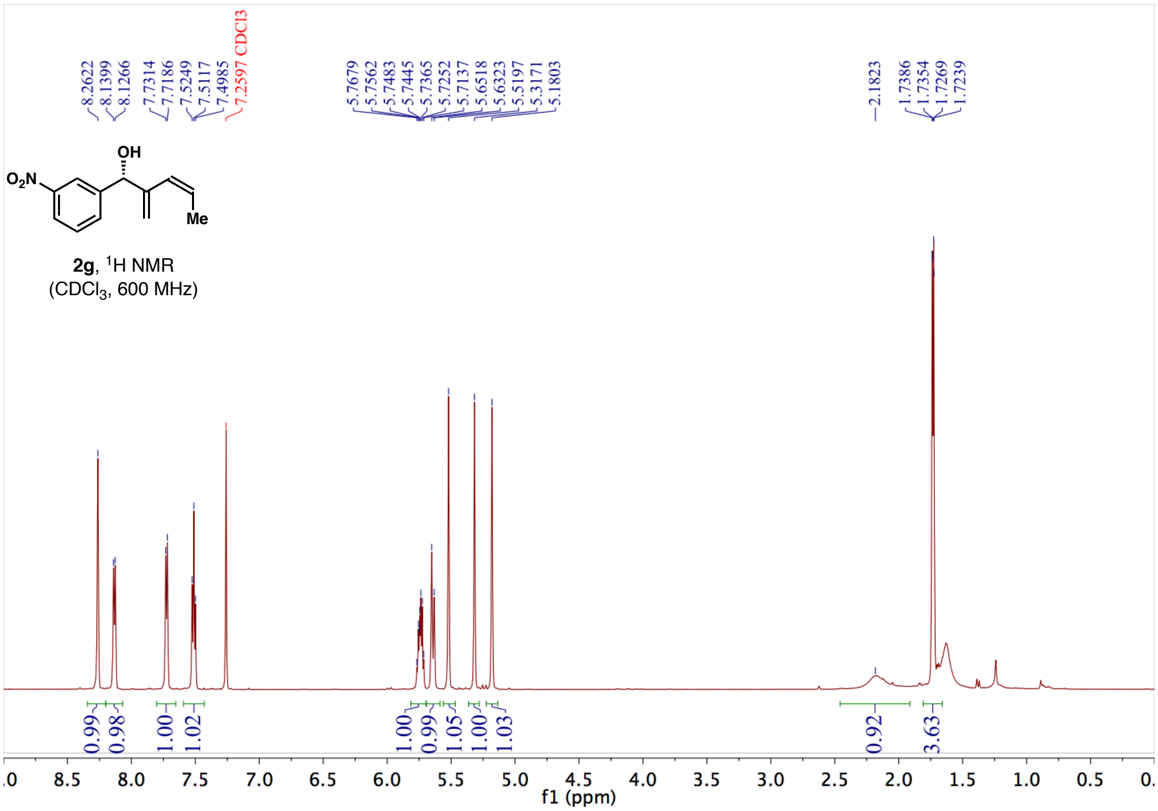
2g, 1H NMR
(CDCl3, 600 MHz)
OH
O2N
Me
f1 (ppm)

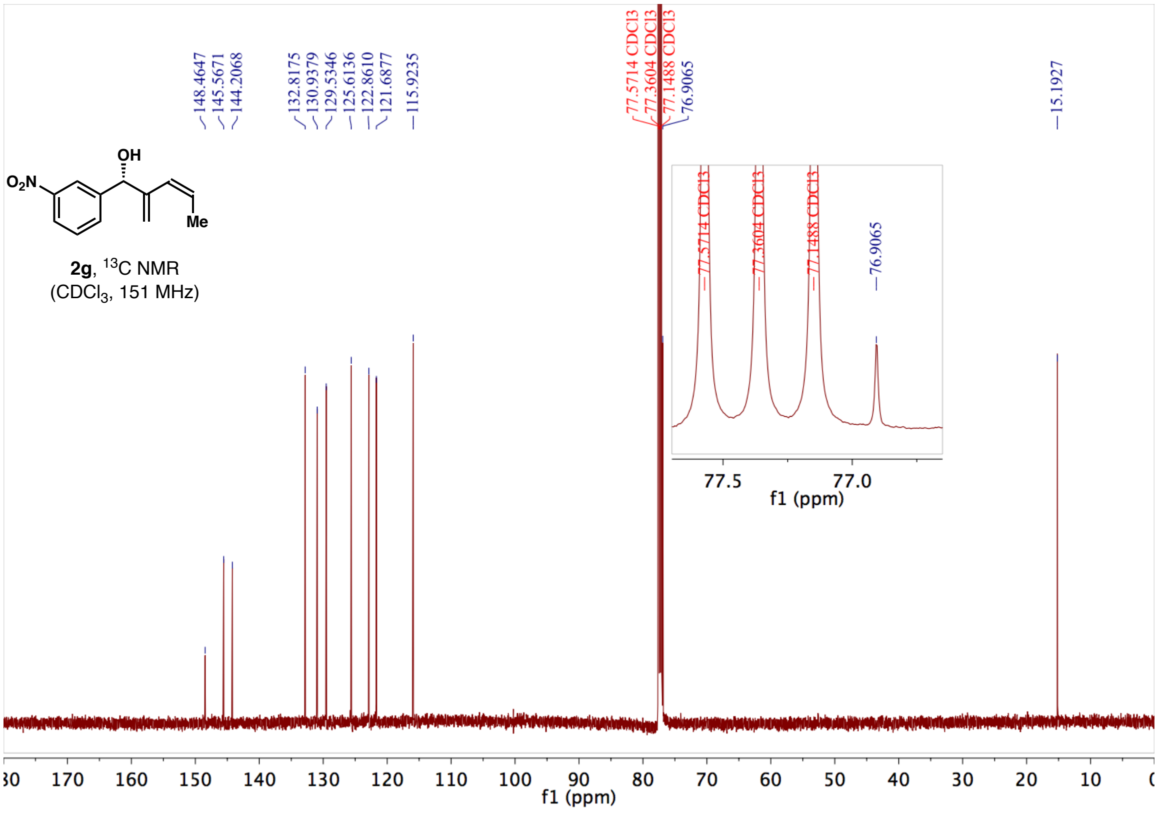
2g, 13C NMR
(CDCl3, 151 MHz)
OH
O2N
Me
f1 (ppm)

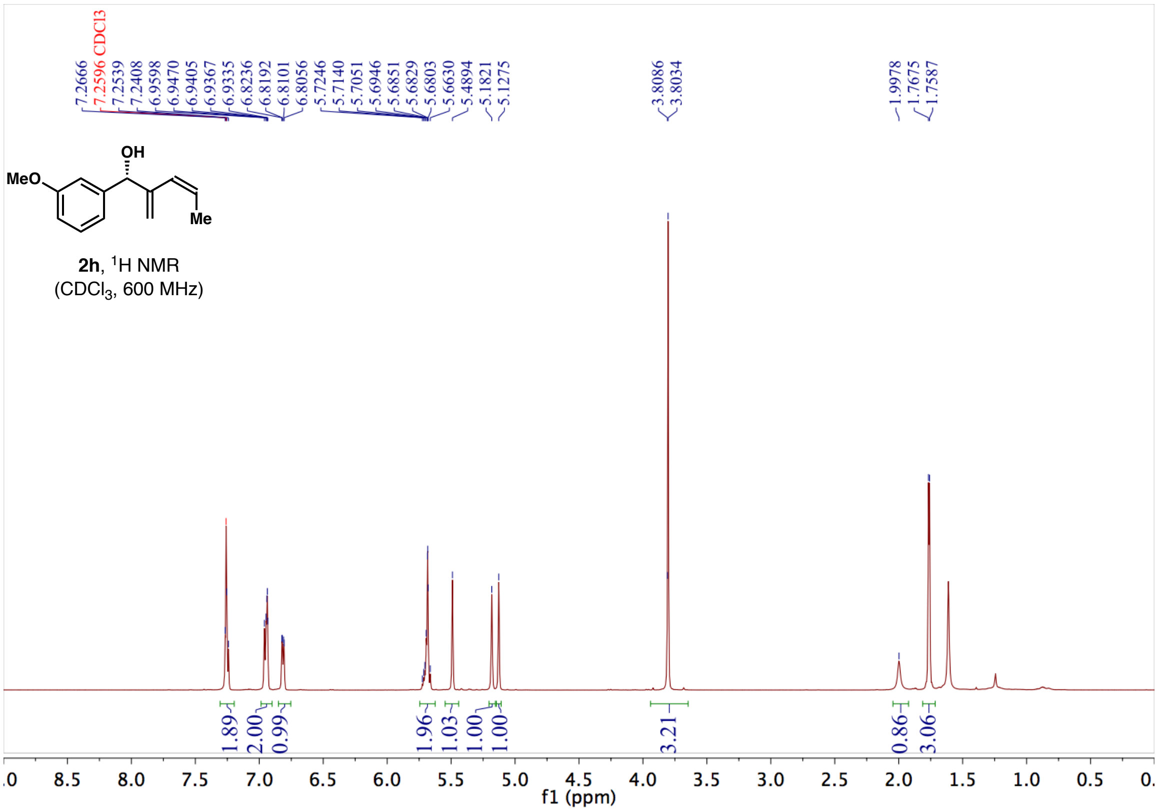
2h, ¹H NMR
(CDCl3, 600 MHz)
f1 (ppm)

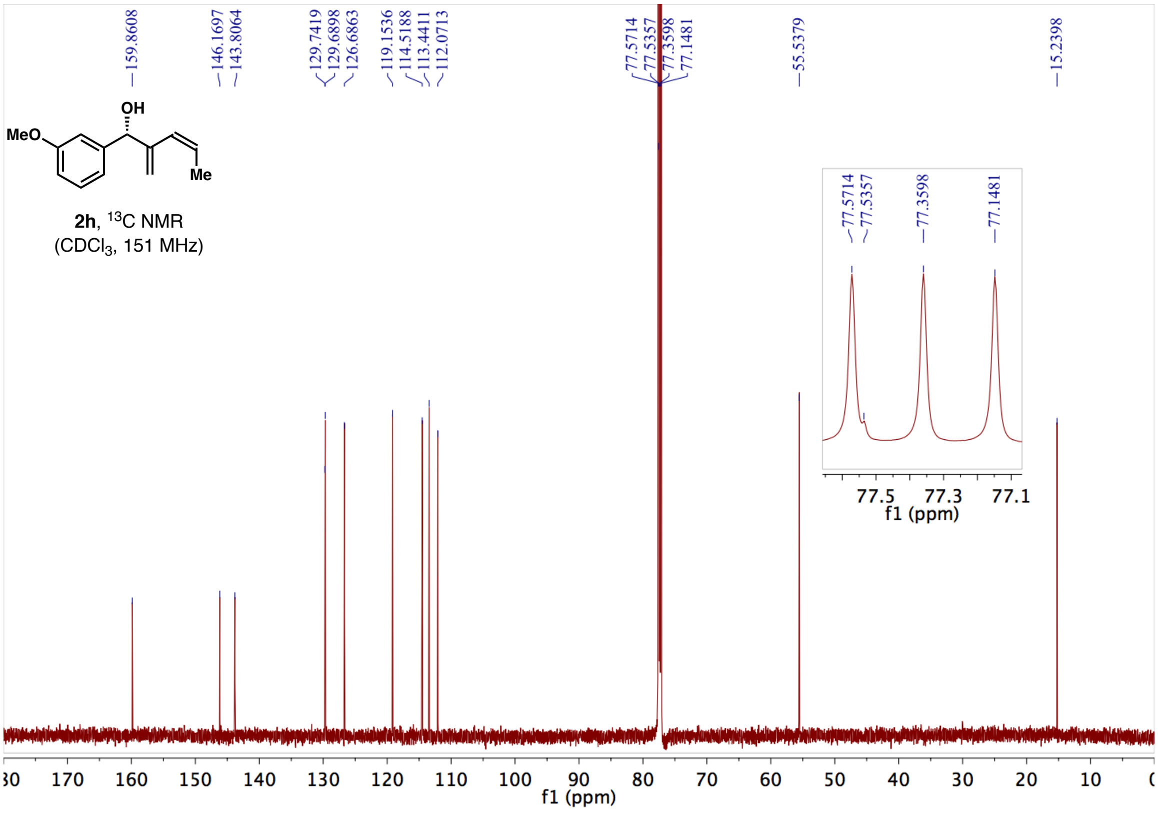
2h, ¹³C NMR
(CDCl3, 151 MHz)
f1 (ppm)

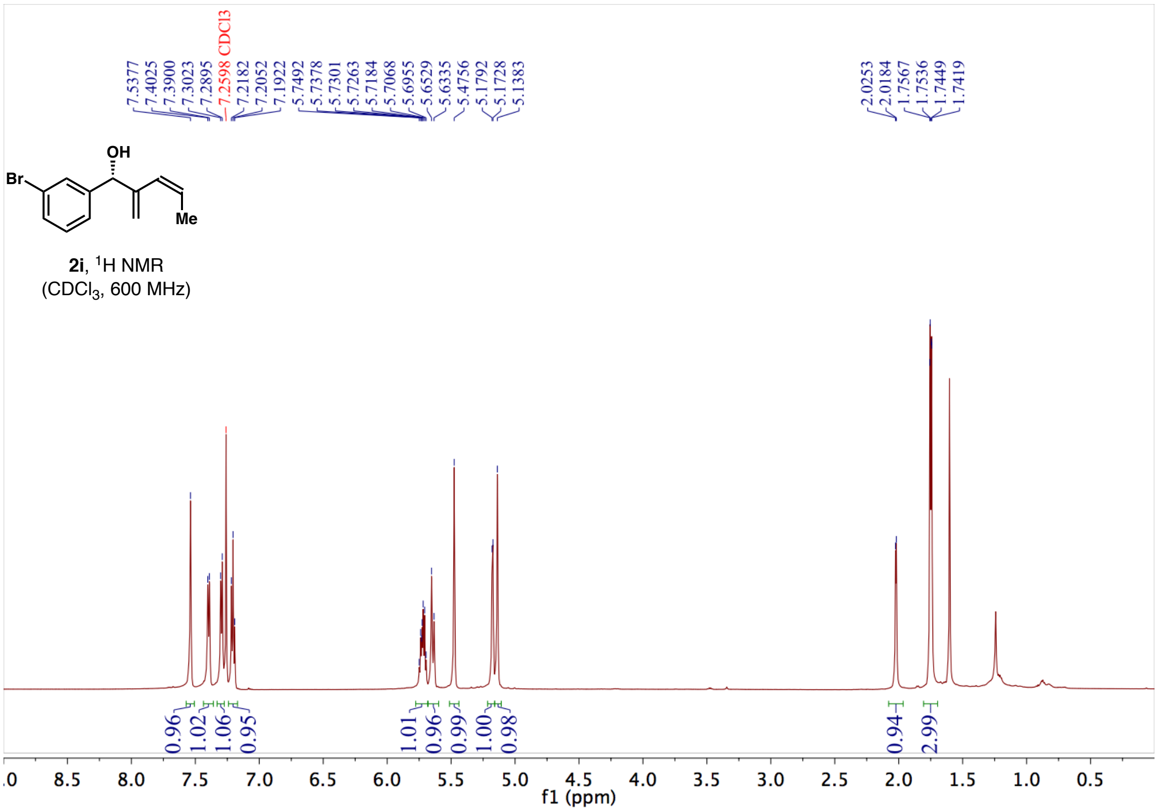
7.5377
7.4025
7.3900
7.3023
7.2895
7.2598 CDCl3
7.2182
7.2052
7.1922
5.7492
5.7378
5.7301
5.7263
5.7184
5.7068
5.6955
5.6529
5.6335
5.4756
5.1792
5.1728
5.1383
2.0253
2.0184
1.7567
1.7536
1.7449
1.7419
OH
Br
Me
2i, 1H NMR
(CDCl3, 600 MHz)
0.96
1.02
1.06
0.95
1.01
0.96
0.99
1.00
0.98
0.94
2.99
f1 (ppm)

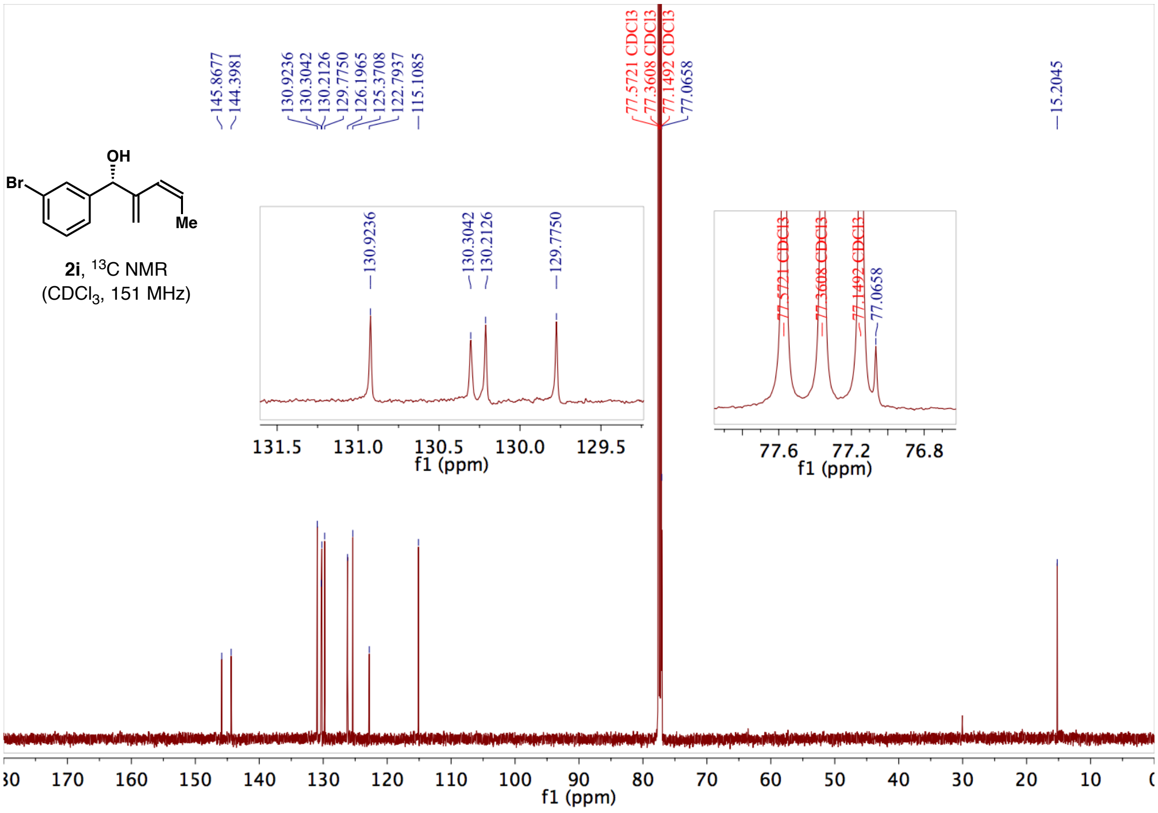
145.8677
144.3981
130.9236
130.3042
130.2126
129.7750
126.1965
125.3708
122.7937
115.1085
77.5721 CDCl3
77.3608 CDCl3
77.1492 CDCl3
77.0658
15.2045
OH
Br
Me
2i, 13C NMR
(CDCl3, 151 MHz)
f1 (ppm)

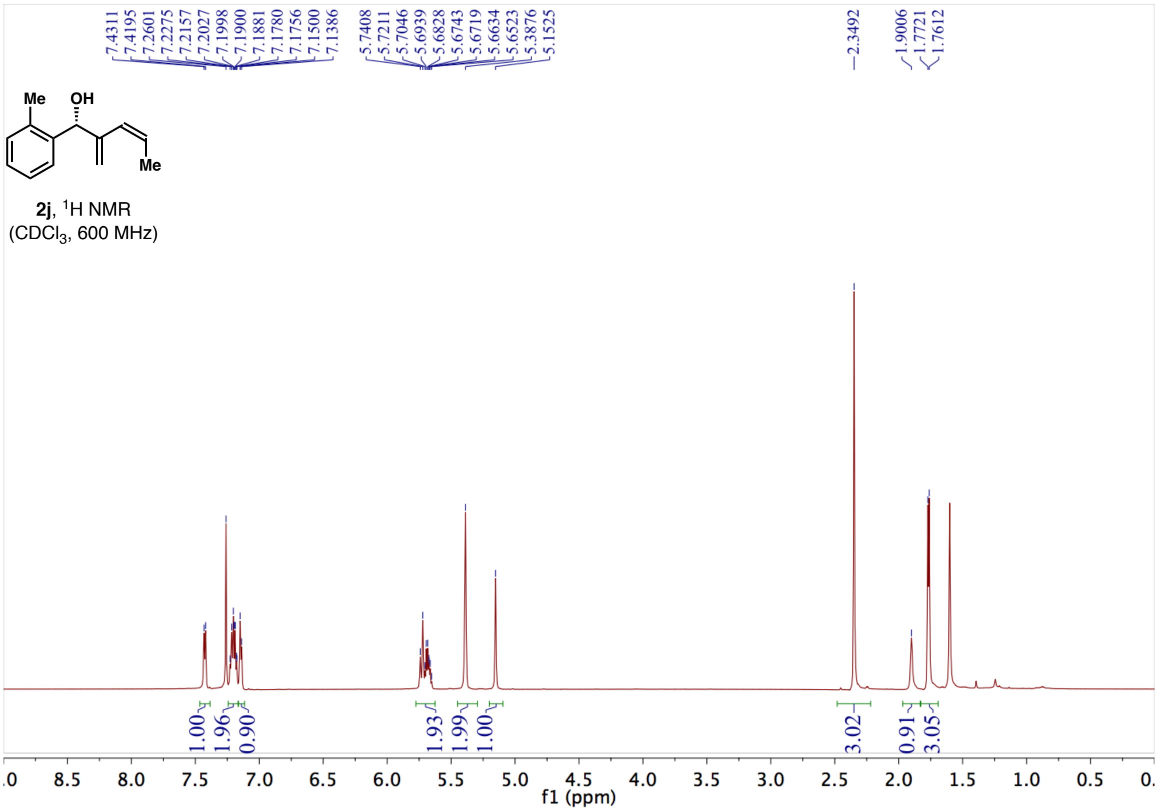
7.4311
7.4195
7.2601
7.2275
7.2157
7.2027
7.1998
7.1900
7.1881
7.1780
7.1756
7.1500
7.1386
5.7408
5.7211
5.7046
5.6939
5.6828
5.6743
5.6719
5.6634
5.6523
5.3876
5.1525
2.3492
1.9006
1.7721
1.7612
Me
OH
Me
2j, 1H NMR
(CDCl3, 600 MHz)
1.00
1.96
0.90
1.93
1.99
1.00
3.02
0.91
3.05
f1 (ppm)

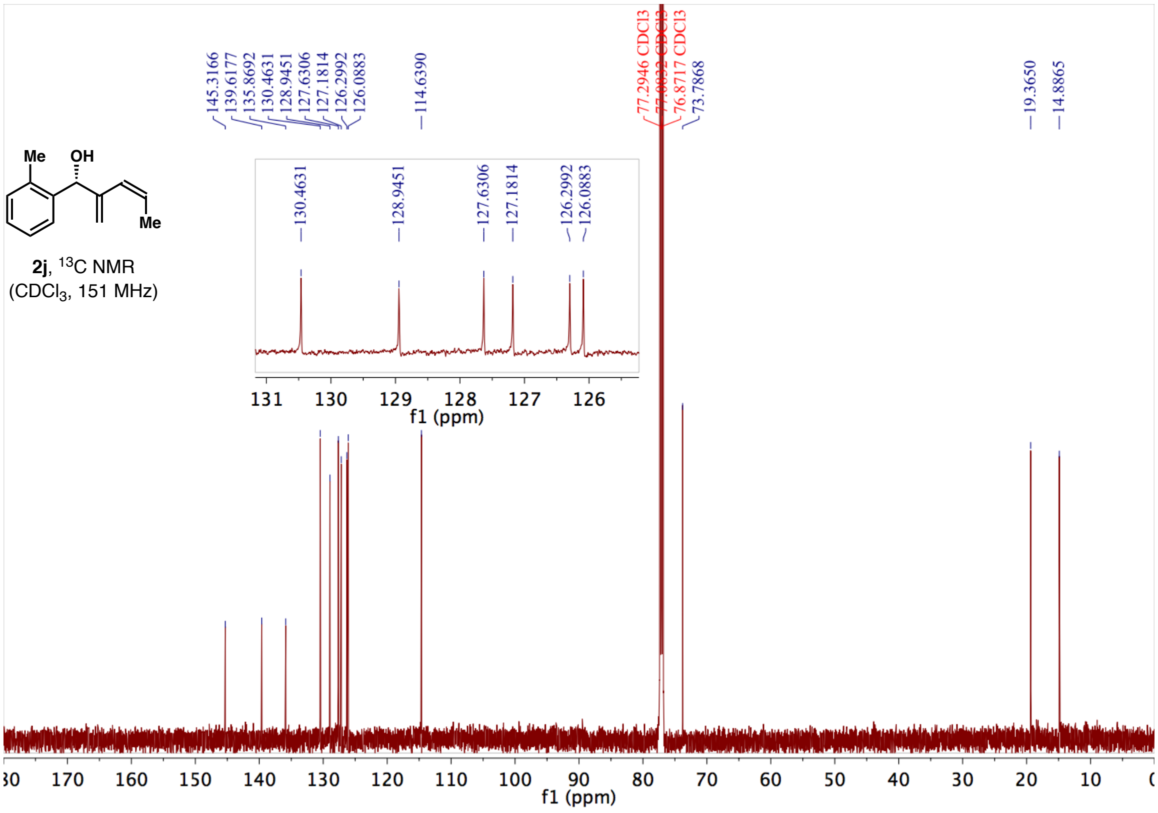
145.3166
139.6177
135.8692
130.4631
128.9451
127.6306
127.1814
126.2992
126.0883
114.6390
77.2946 CDCl3
77.0832 CDCl3
76.8717 CDCl3
73.7868
19.3650
14.8865
Me
OH
Me
2j, 13C NMR
(CDCl3, 151 MHz)
f1 (ppm)

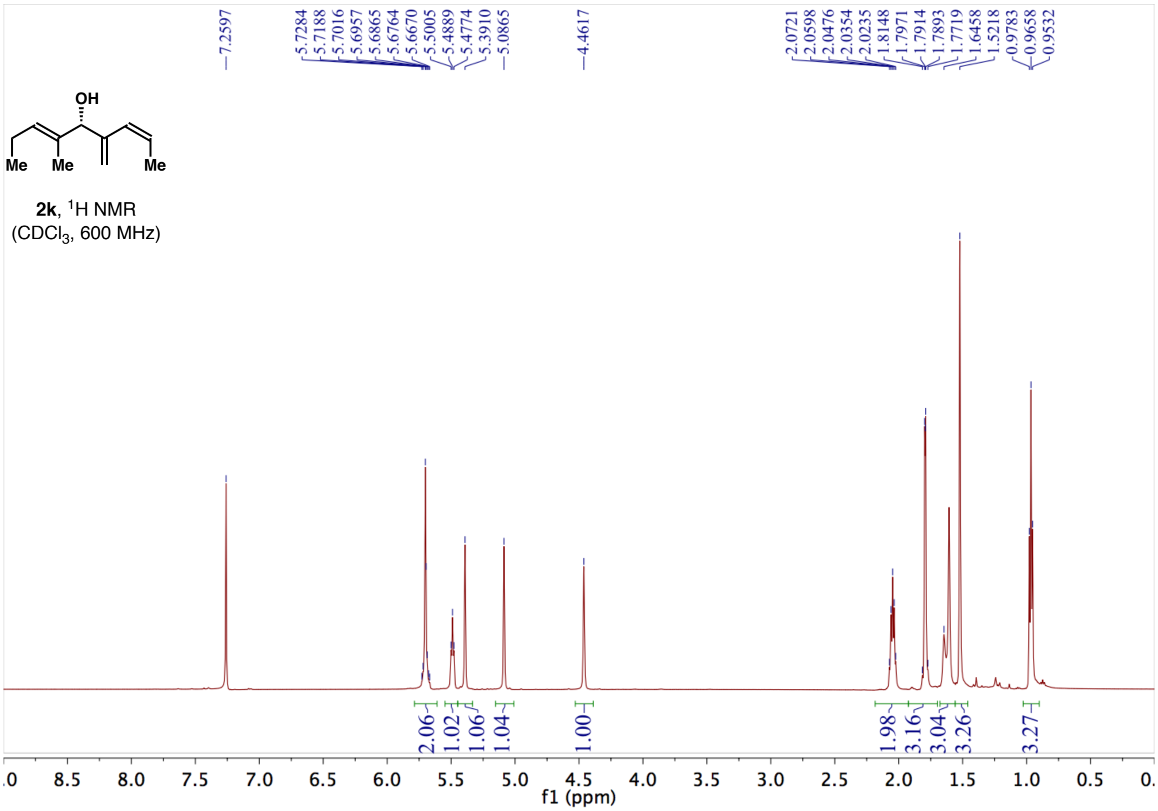

7.2597
5.7284
5.7188
5.7016
5.6957
5.6865
5.6764
5.6670
5.5005
5.4889
5.4774
5.3910
5.0865
4.4617
2.0721
2.0598
2.0476
2.0354
2.0235
1.8148
1.7971
1.7914
1.7893
1.7719
1.6458
1.5218
0.9783
0.9658
0.9532
OH
Me
Me
Me
2k, 1H NMR
(CDCl3, 600 MHz)
2.06
1.02
1.06
1.04
1.00
1.98
3.16
3.04
3.26
3.27
f1 (ppm)

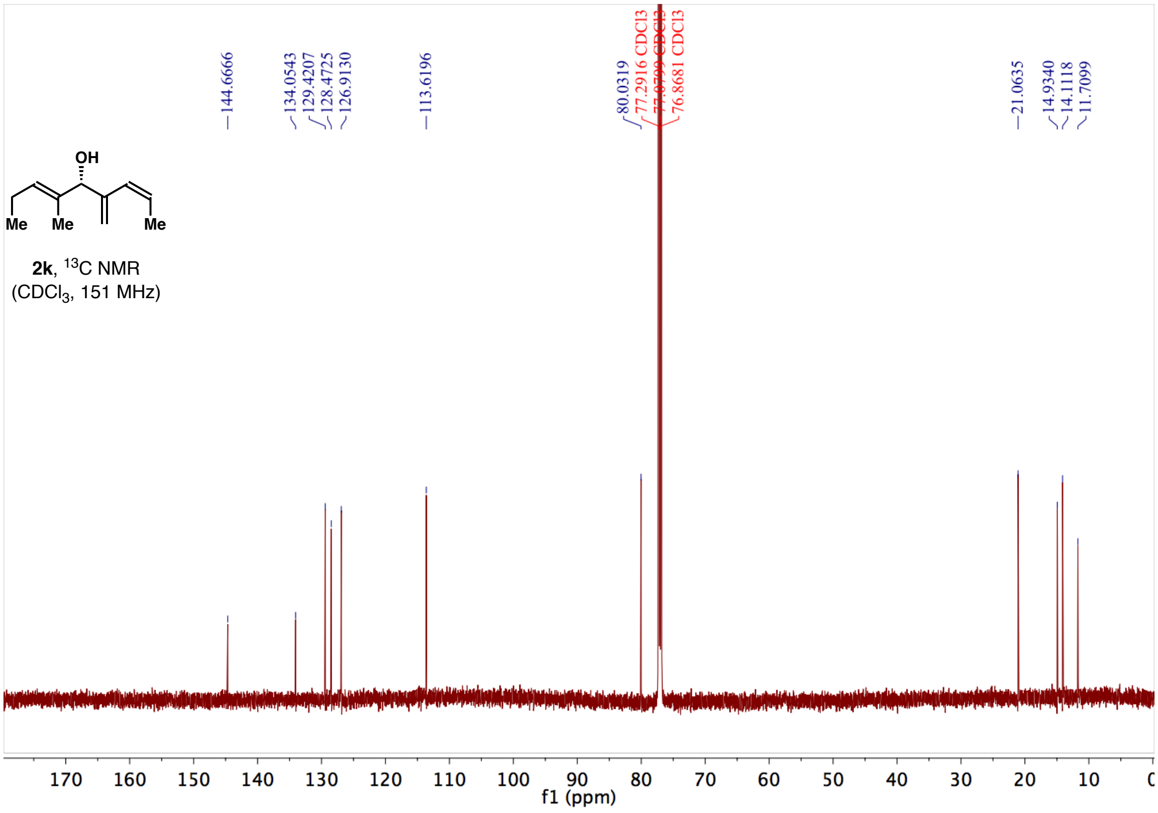

144.6666
134.0543
129.4207
128.4725
126.9130
113.6196
80.0319
77.2916 CDCl3
76.8681 CDCl3
21.0635
14.9340
14.1118
11.7099
OH
Me
Me
Me
2k, 13C NMR
(CDCl3, 151 MHz)
f1 (ppm)

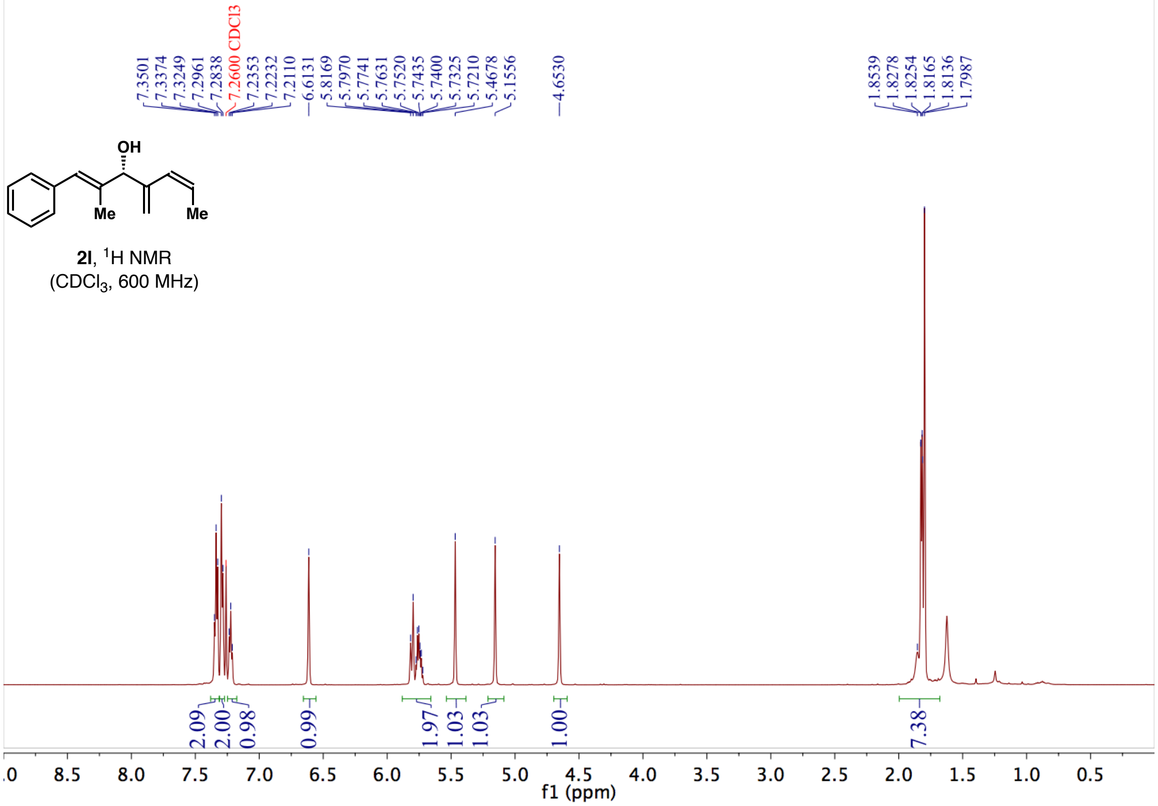
7.3501
7.3374
7.3249
7.2961
7.2838
7.2600 CDCl3
7.2353
7.2232
7.2110
6.6131
5.8169
5.7970
5.7741
5.7631
5.7520
5.7435
5.7400
5.7325
5.7210
5.4678
5.1556
4.6530
1.8539
1.8278
1.8254
1.8165
1.8136
1.7987
OH
Me
Me
2l, ¹H NMR
(CDCl3, 600 MHz)
2.09
2.00
0.98
0.99
1.97
1.03
1.03
1.00
7.38
f1 (ppm)

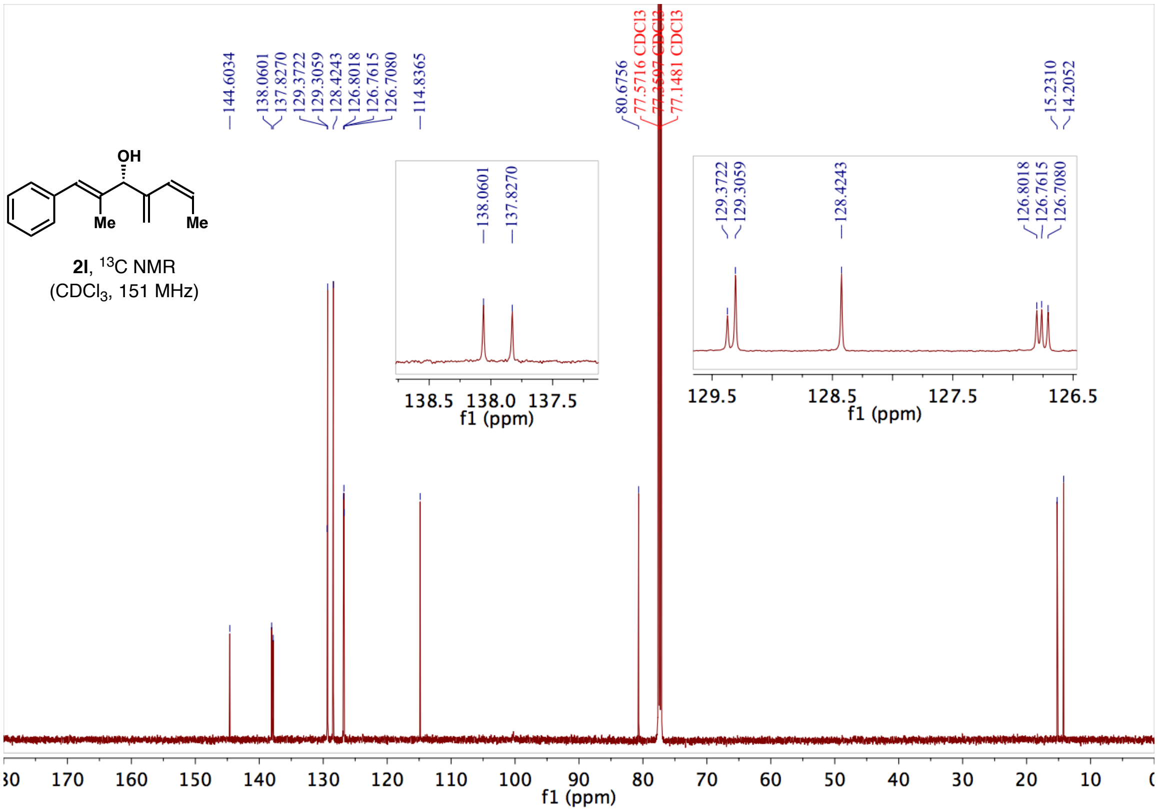
144.6034
138.0601
137.8270
129.3722
129.3059
128.4243
126.8018
126.7615
126.7080
114.8365
80.6756
77.5716 CDCl3
77.3597 CDCl3
77.1481 CDCl3
15.2310
14.2052
OH
Me
Me
2l, ¹³C NMR
(CDCl3, 151 MHz)
f1 (ppm)

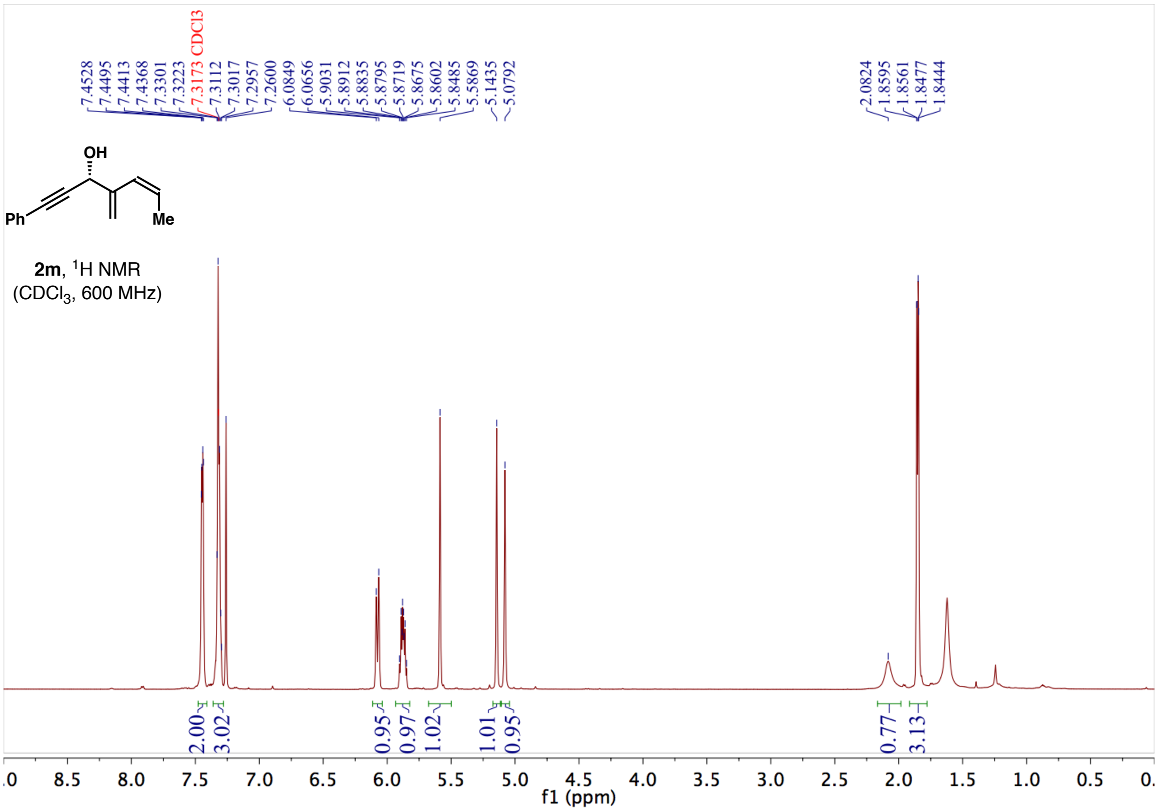
OH
Ph
Me
2m, ¹H NMR
(CDCl₃, 600 MHz)
f1 (ppm)

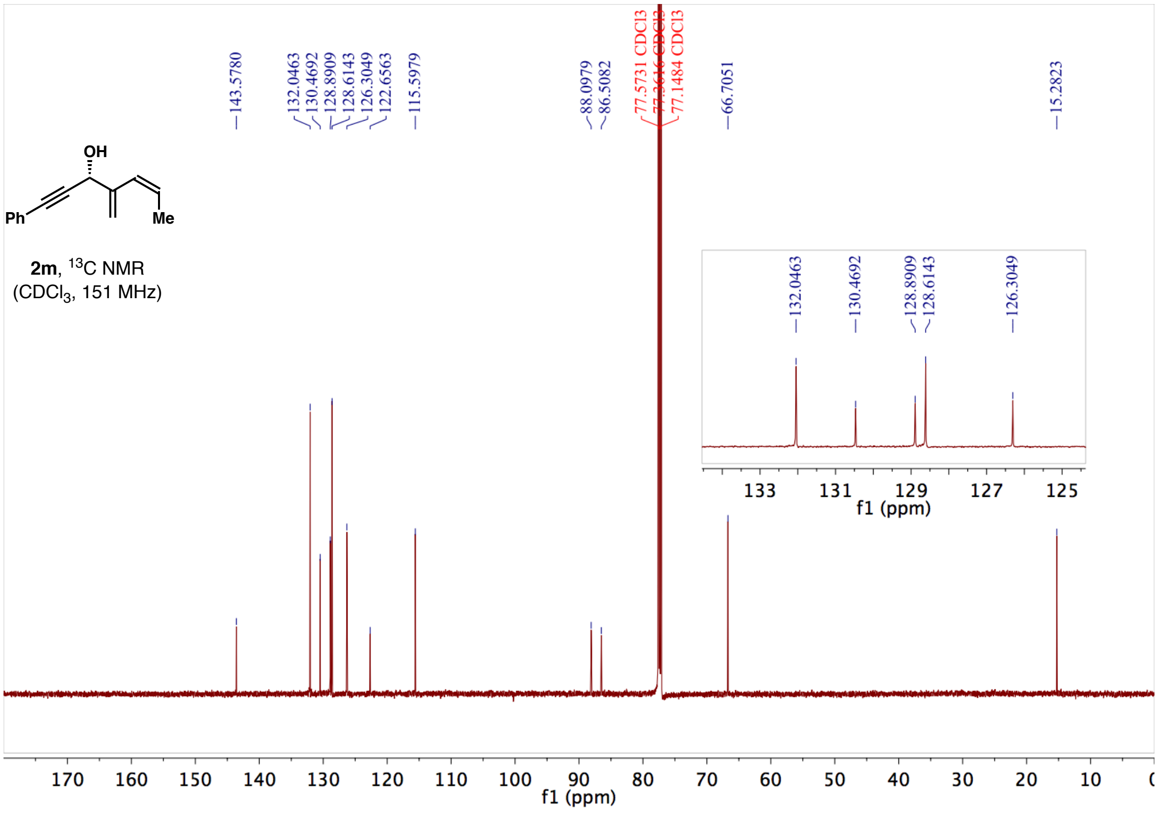
OH
Ph
Me
2m, ¹³C NMR
(CDCl₃, 151 MHz)
f1 (ppm)

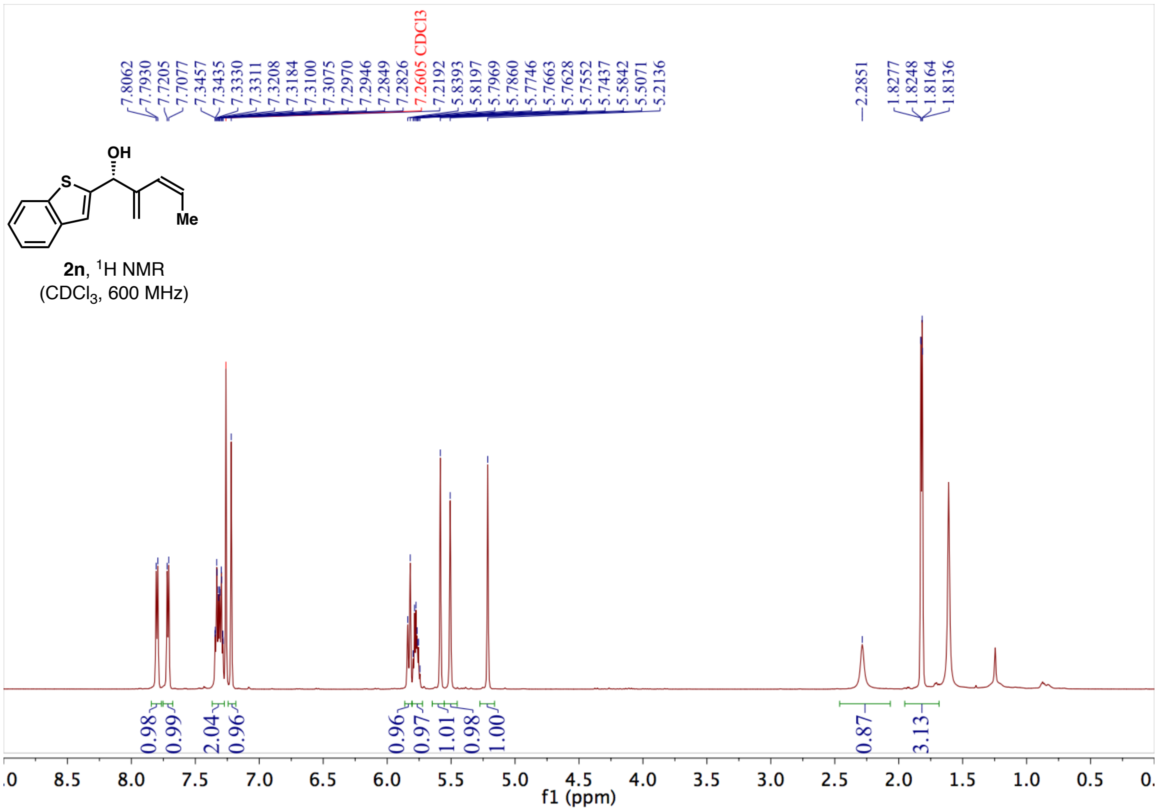

2n, ¹H NMR
(CDCl3, 600 MHz)
f1 (ppm)

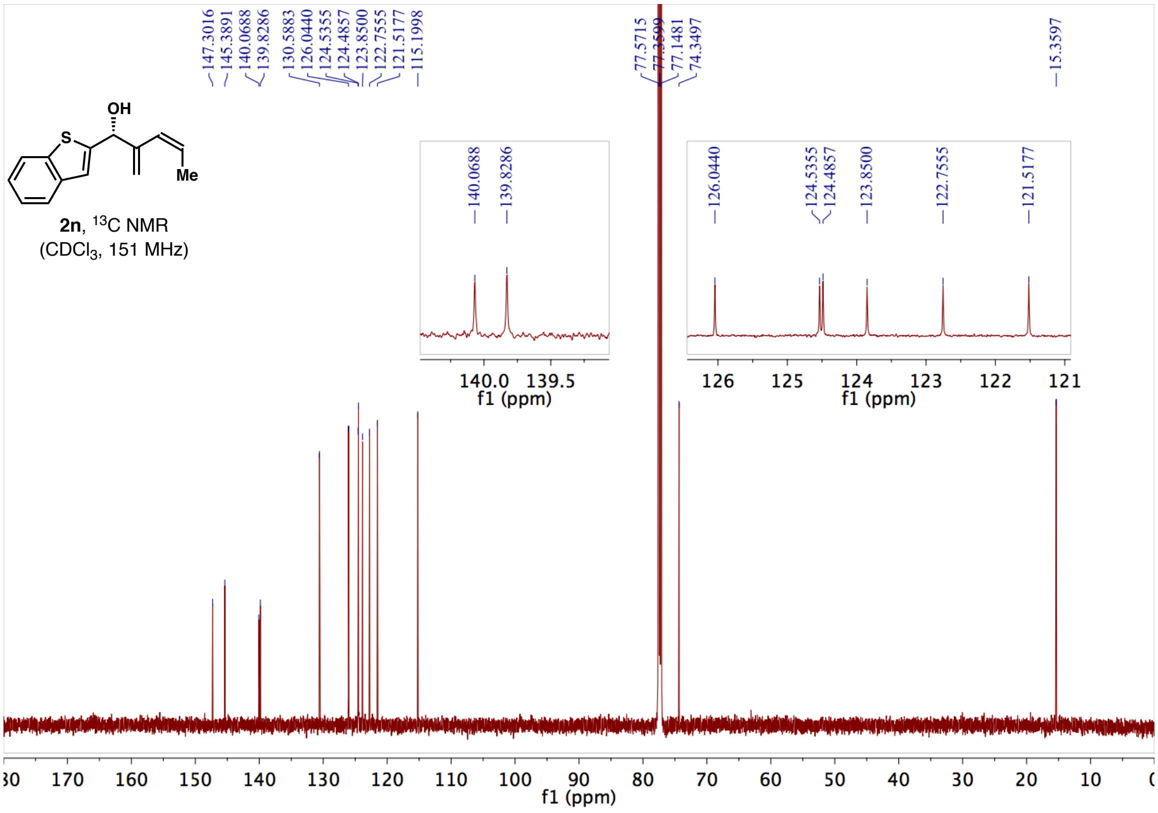

2n, ¹³C NMR
(CDCl3, 151 MHz)
f1 (ppm)

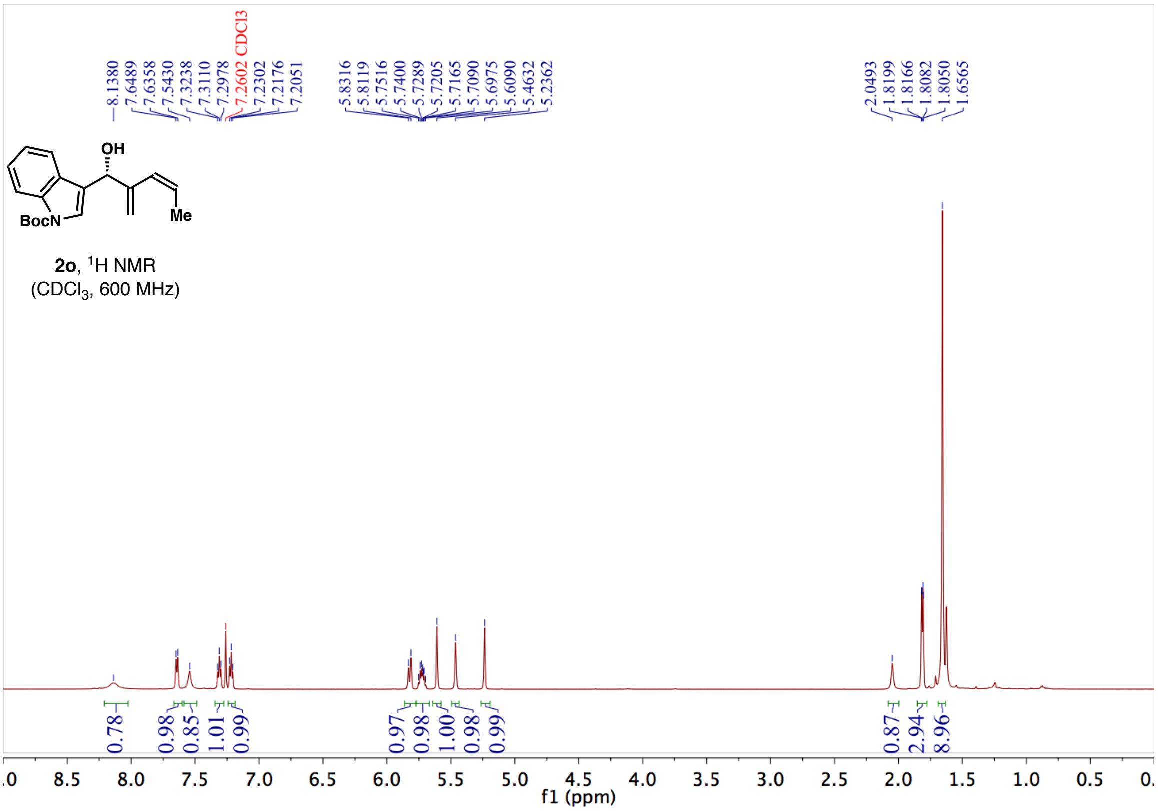
8.1380
7.6489
7.6358
7.5430
7.3238
7.3110
7.2978
7.2602 CDCl3
7.2302
7.2176
7.2051
5.8316
5.8119
5.7516
5.7400
5.7289
5.7205
5.7165
5.7090
5.6975
5.6090
5.4632
5.2362
2.0493
1.8199
1.8166
1.8082
1.8050
1.6565
OH
BocN
Me
2o, 1H NMR
(CDCl3, 600 MHz)
0.78
0.98
0.85
1.01
0.99
0.97
0.98
1.00
0.98
0.99
0.87
2.94
8.96
8.5
8.0
7.5
7.0
6.5
6.0
5.5
5.0
4.5
4.0
3.5
3.0
2.5
2.0
1.5
1.0
0.5
f1 (ppm)

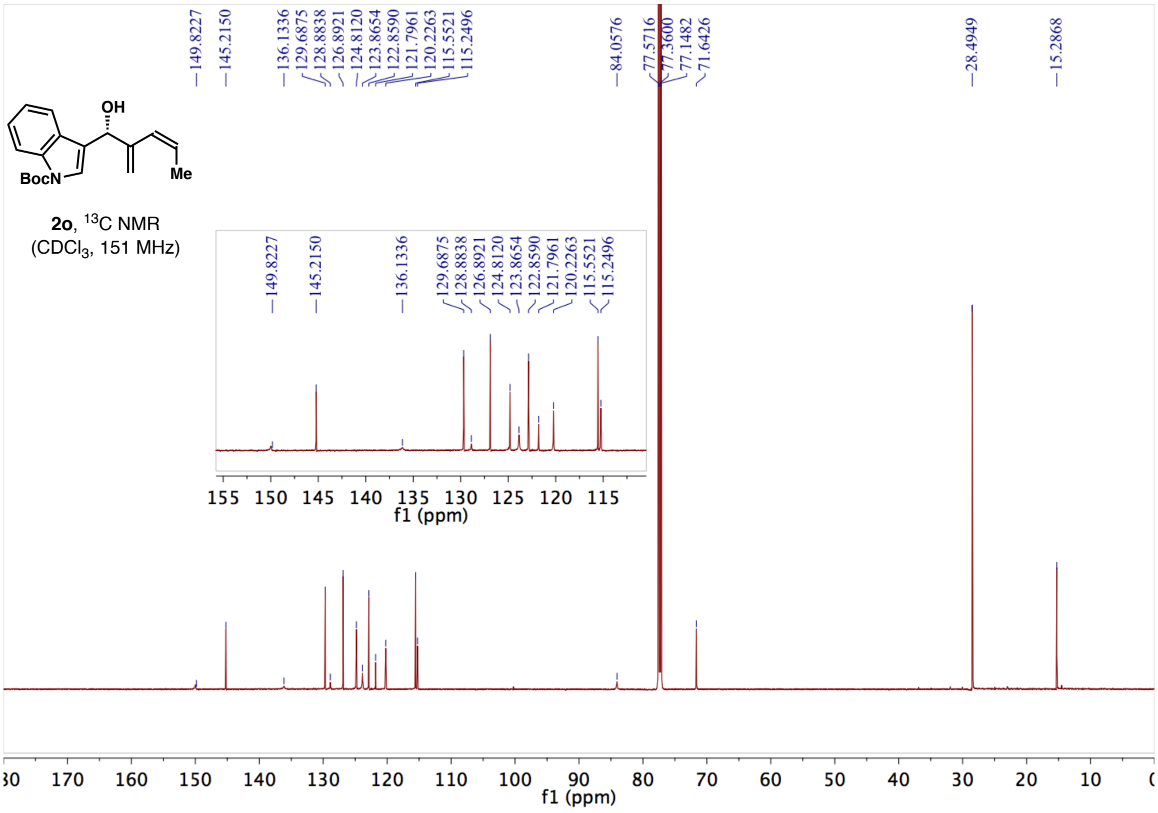
149.8227
145.2150
136.1336
129.6875
128.8838
126.8921
124.8120
123.8654
122.8590
121.7961
120.2263
115.5521
115.2496
84.0576
77.5716
77.3600
77.1482
71.6426
28.4949
15.2868
OH
BocN
Me
2o, 13C NMR
(CDCl3, 151 MHz)
155
150
145
140
135
130
125
120
115
f1 (ppm)
170
160
150
140
130
120
110
100
90
80
70
60
50
40
30
20
10
f1 (ppm)

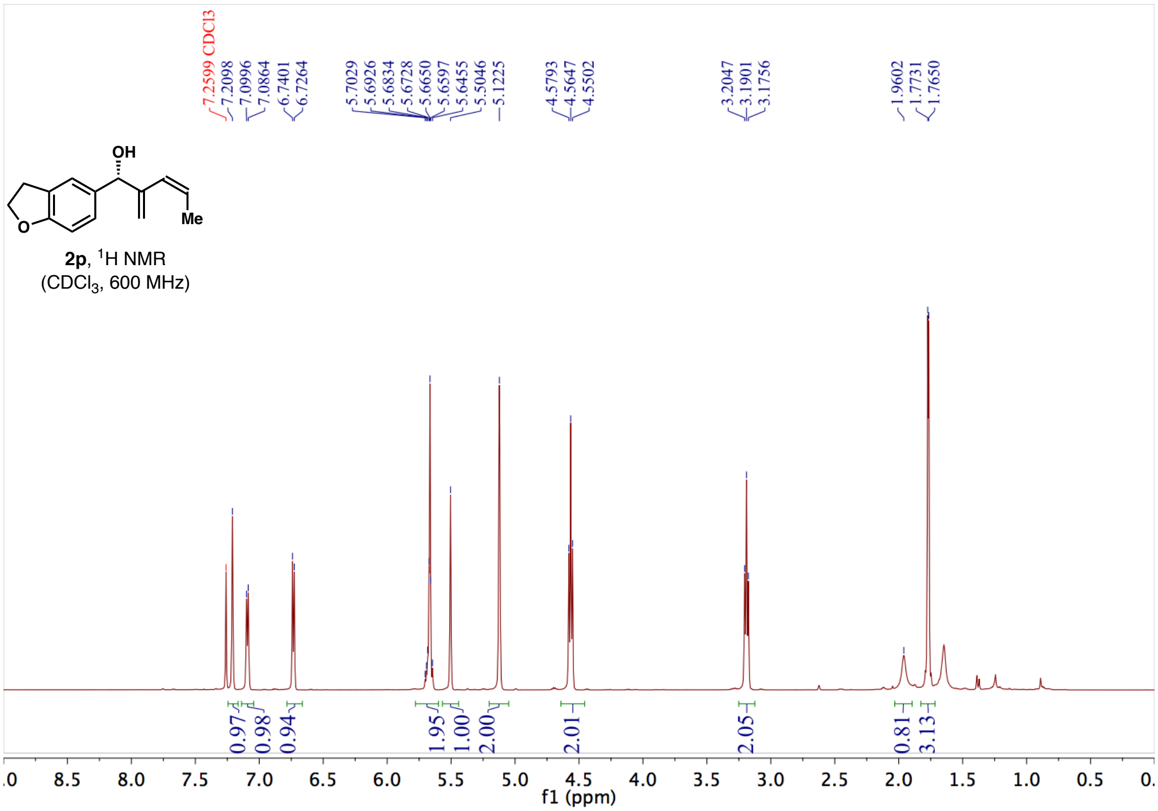
7.2599 CDCl3
7.2098
7.0996
7.0864
6.7401
6.7264
5.7029
5.6926
5.6834
5.6728
5.6650
5.6597
5.6455
5.5046
5.1225
4.5793
4.5647
4.5502
3.2047
3.1901
3.1756
1.9602
1.7731
1.7650
OH
O
Me
2p, 1H NMR
(CDCl3, 600 MHz)
0.97
0.98
0.94
1.95
1.00
2.00
2.01
2.05
0.81
3.13
f1 (ppm)

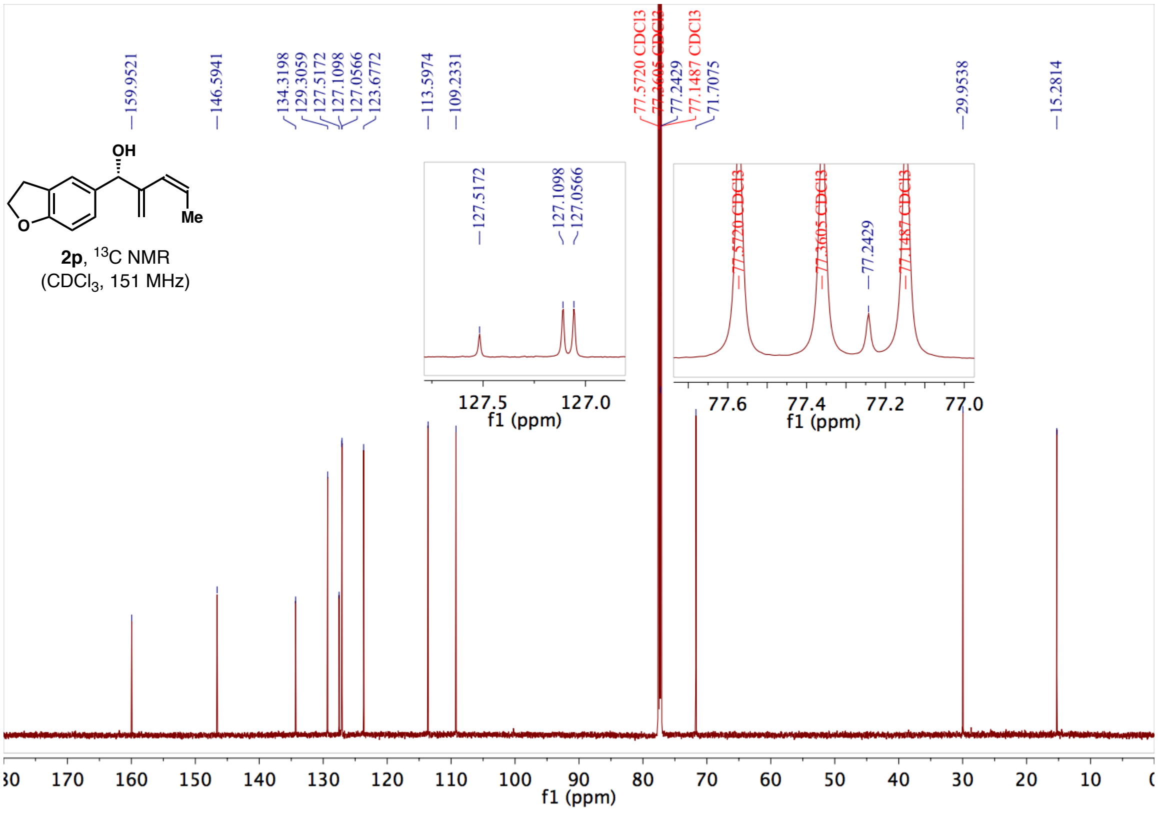
159.9521
146.5941
134.3198
129.3059
127.5172
127.1098
127.0566
123.6772
113.5974
109.2331
77.5720 CDCl3
77.3605 CDCl3
77.2429
77.1487 CDCl3
71.7075
29.9538
15.2814
OH
O
Me
2p, 13C NMR
(CDCl3, 151 MHz)
f1 (ppm)

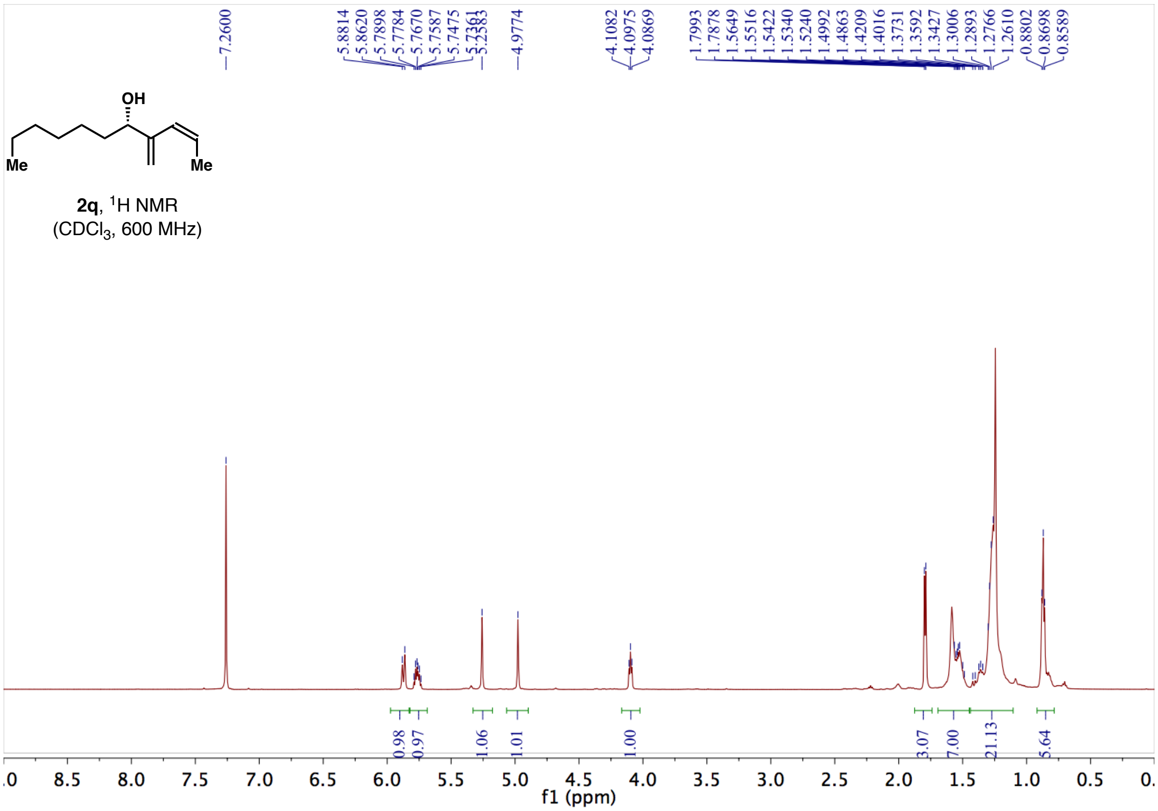
7.2600
5.8814
5.8620
5.7898
5.7784
5.7670
5.7587
5.7475
5.7361
5.2583
4.9774
4.1082
4.0975
4.0869
1.7993
1.7878
1.5649
1.5516
1.5422
1.5340
1.5240
1.4992
1.4863
1.4209
1.4016
1.3731
1.3592
1.3427
1.3006
1.2893
1.2766
1.2610
0.8802
0.8698
0.8589
OH
Me
Me
2q, 1H NMR
(CDCl3, 600 MHz)
0.98
0.97
1.06
1.01
1.00
3.07
7.00
21.13
5.64
f1 (ppm)

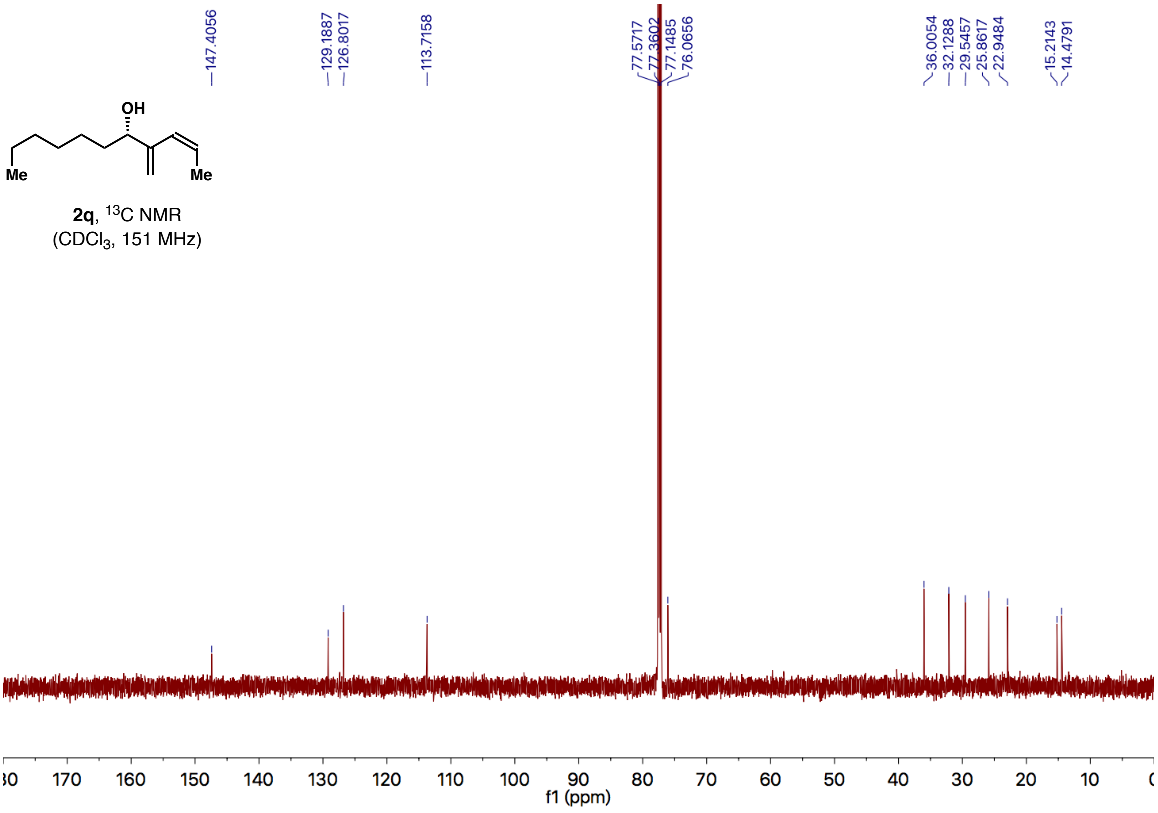
147.4056
129.1887
126.8017
113.7158
77.5717
77.3602
77.1485
76.0656
36.0054
32.1288
29.5457
25.8617
22.9484
15.2143
14.4791
OH
Me
Me
2q, 13C NMR
(CDCl3, 151 MHz)
f1 (ppm)

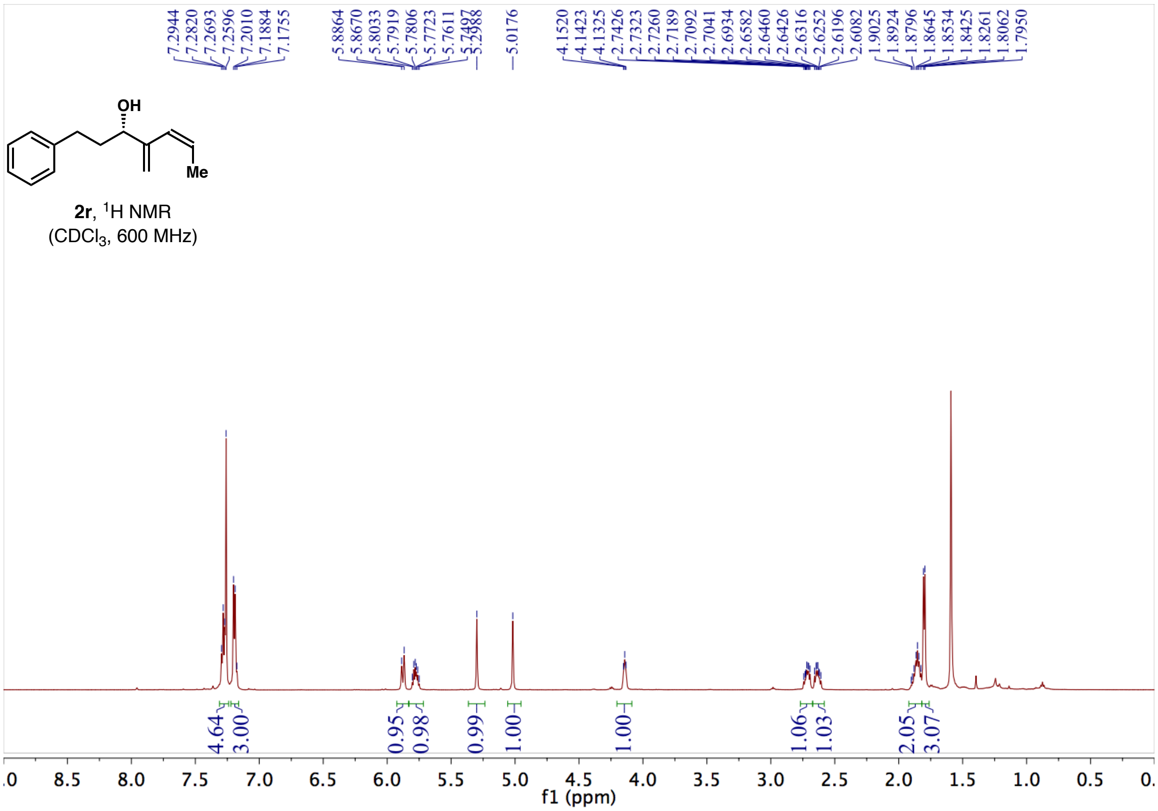
2r, ¹H NMR
(CDCl3, 600 MHz)
f1 (ppm)

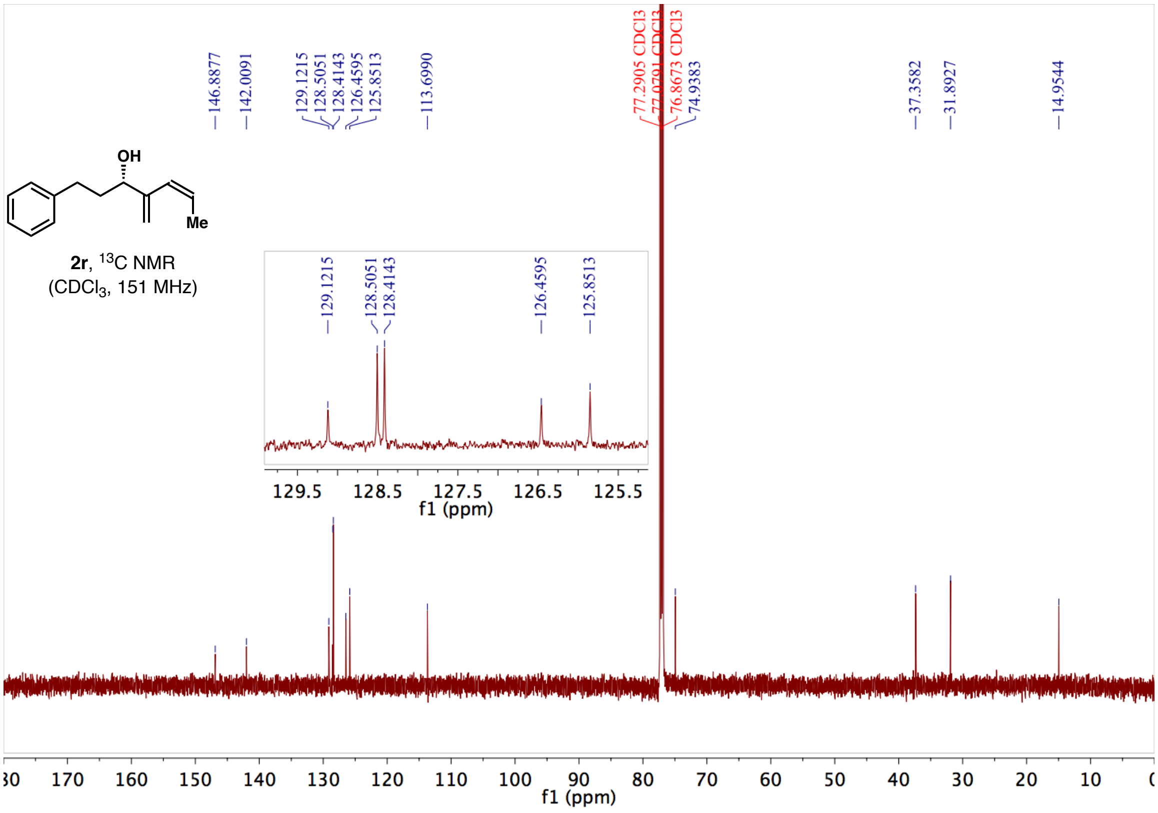
2r, ¹³C NMR
(CDCl3, 151 MHz)
f1 (ppm)

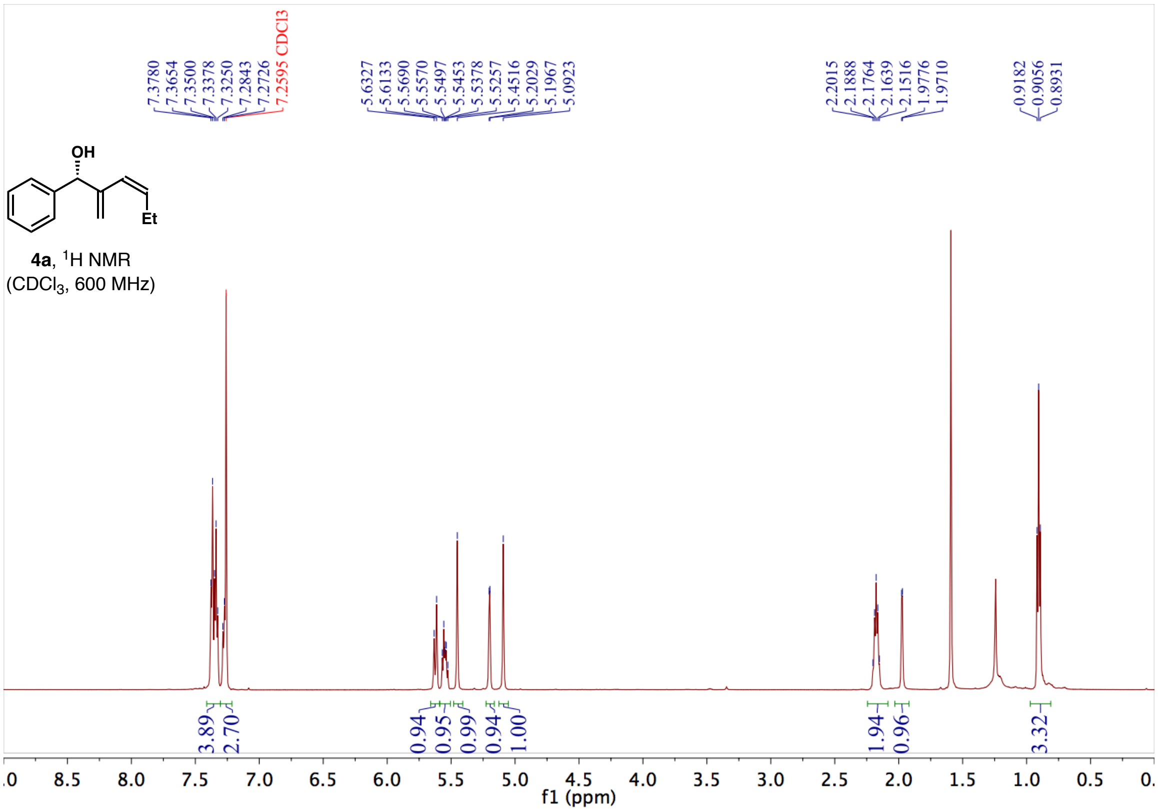
7.3780
7.3654
7.3500
7.3378
7.3250
7.2843
7.2726
7.2595 CDCl3
5.6327
5.6133
5.5690
5.5570
5.5497
5.5453
5.5378
5.5257
5.4516
5.2029
5.1967
5.0923
2.2015
2.1888
2.1764
2.1639
2.1516
1.9776
1.9710
0.9182
0.9056
0.8931
OH
Et
4a, 1H NMR
(CDCl3, 600 MHz)
3.89
2.70
0.94
0.95
0.99
0.94
1.00
1.94
0.96
3.32
f1 (ppm)

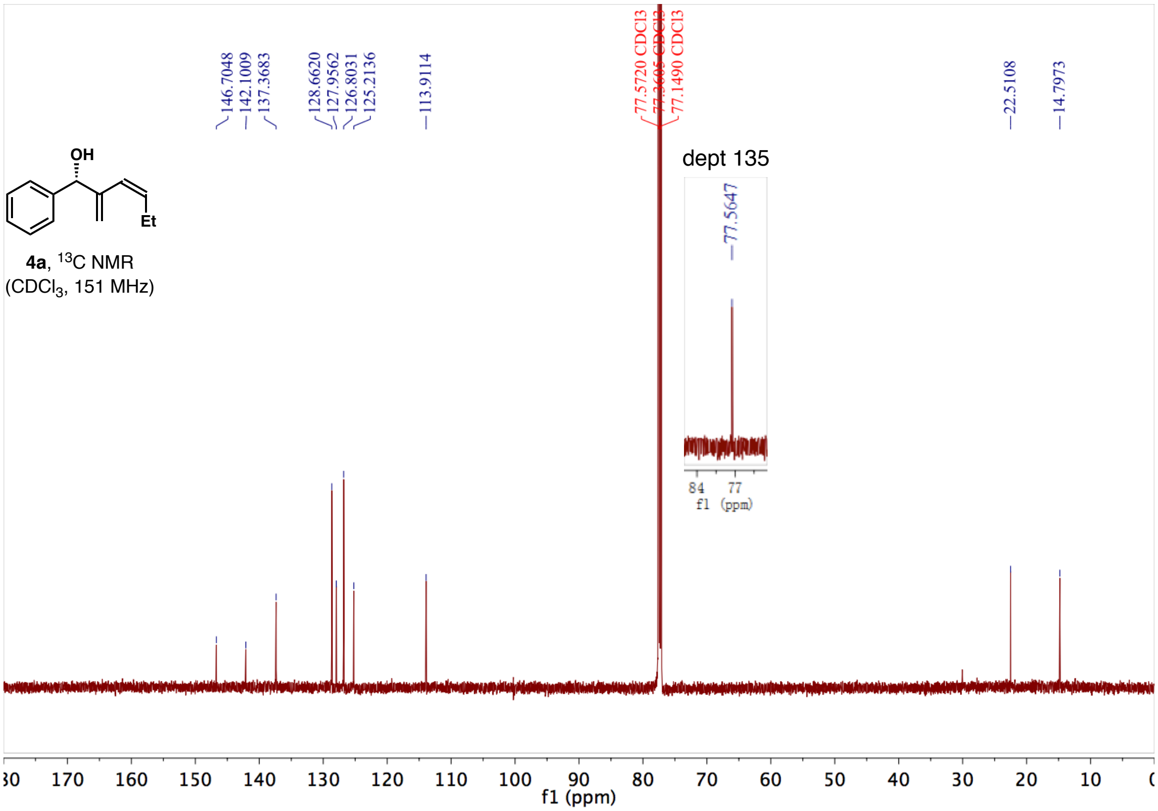
146.7048
142.1009
137.3683
128.6620
127.9562
126.8031
125.2136
113.9114
77.5720 CDCl3
77.1490 CDCl3
22.5108
14.7973
dept 135
77.5647
f1 (ppm)
OH
Et
4a, 13C NMR
(CDCl3, 151 MHz)
f1 (ppm)

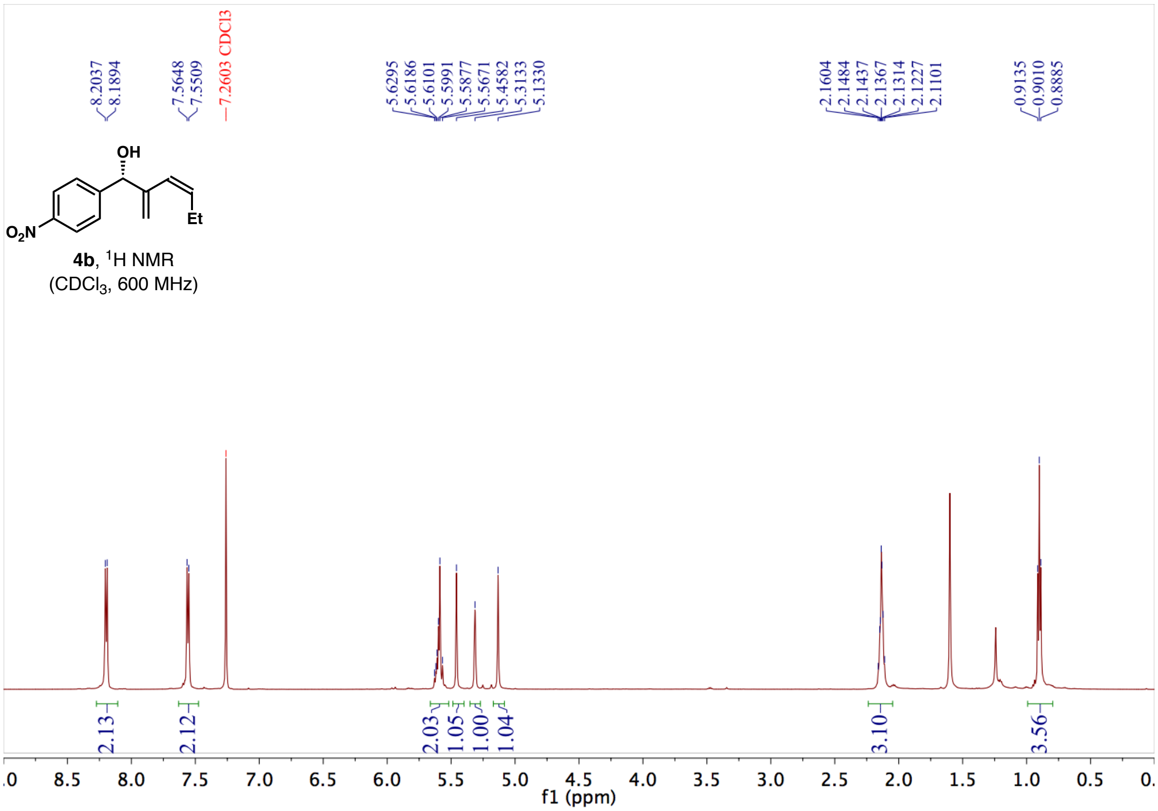

8.2037
8.1894
7.5648
7.5509
7.2603 CDCl3
5.6295
5.6186
5.6101
5.5991
5.5877
5.5671
5.4582
5.3133
5.1330
2.1604
2.1484
2.1437
2.1367
2.1314
2.1227
2.1101
0.9135
0.9010
0.8885
OH
O2N
Et
4b, 1H NMR
(CDCl3, 600 MHz)
2.13
2.12
2.03
1.05
1.00
1.04
3.10
3.56
f1 (ppm)

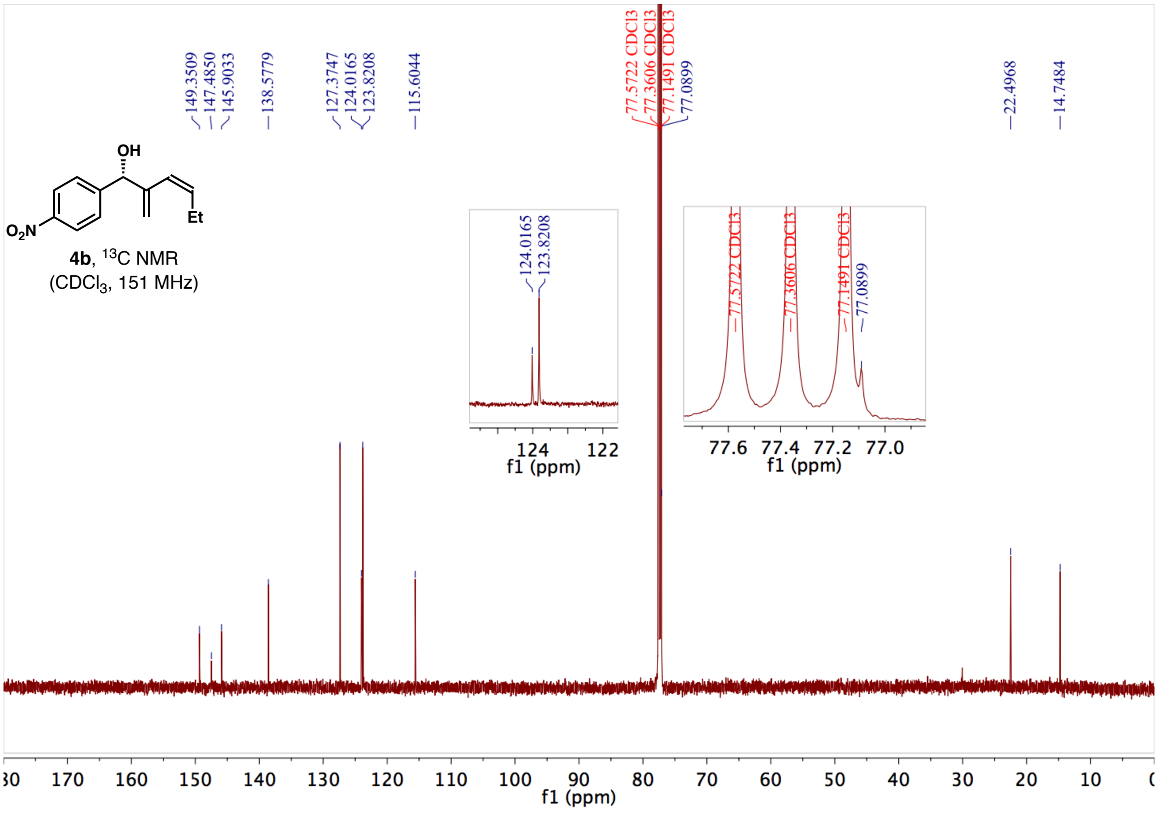

149.3509
147.4850
145.9033
138.5779
127.3747
124.0165
123.8208
115.6044
77.5722 CDCl3
77.3606 CDCl3
77.1491 CDCl3
77.0899
22.4968
14.7484
OH
O2N
Et
4b, 13C NMR
(CDCl3, 151 MHz)
f1 (ppm)

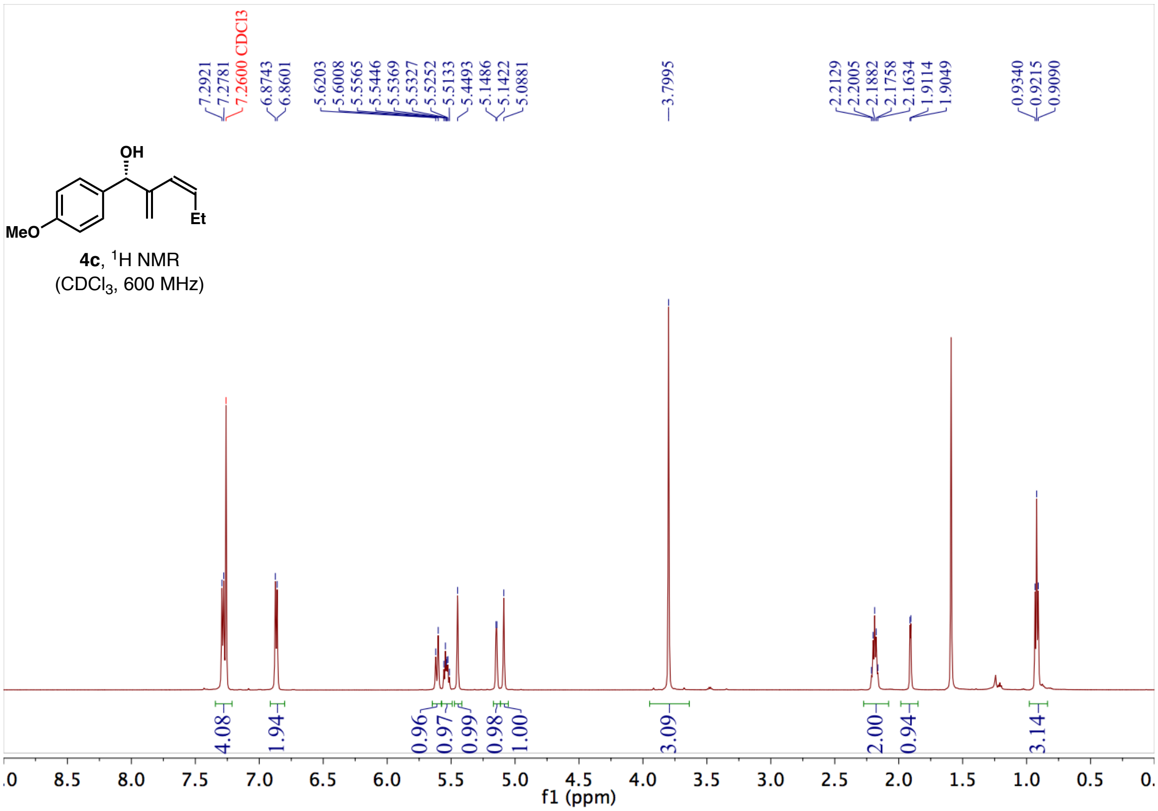
4c, ¹H NMR
(CDCl3, 600 MHz)
f1 (ppm)

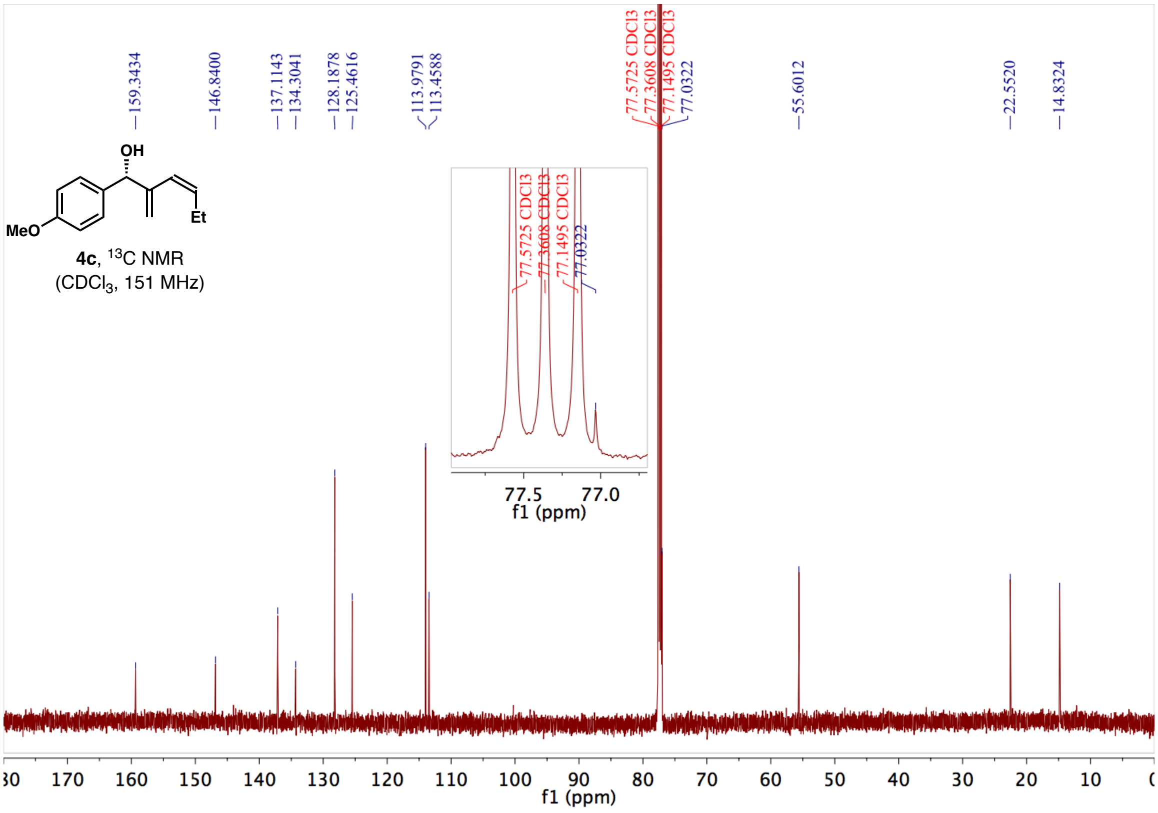
4c, ¹³C NMR
(CDCl3, 151 MHz)
f1 (ppm)

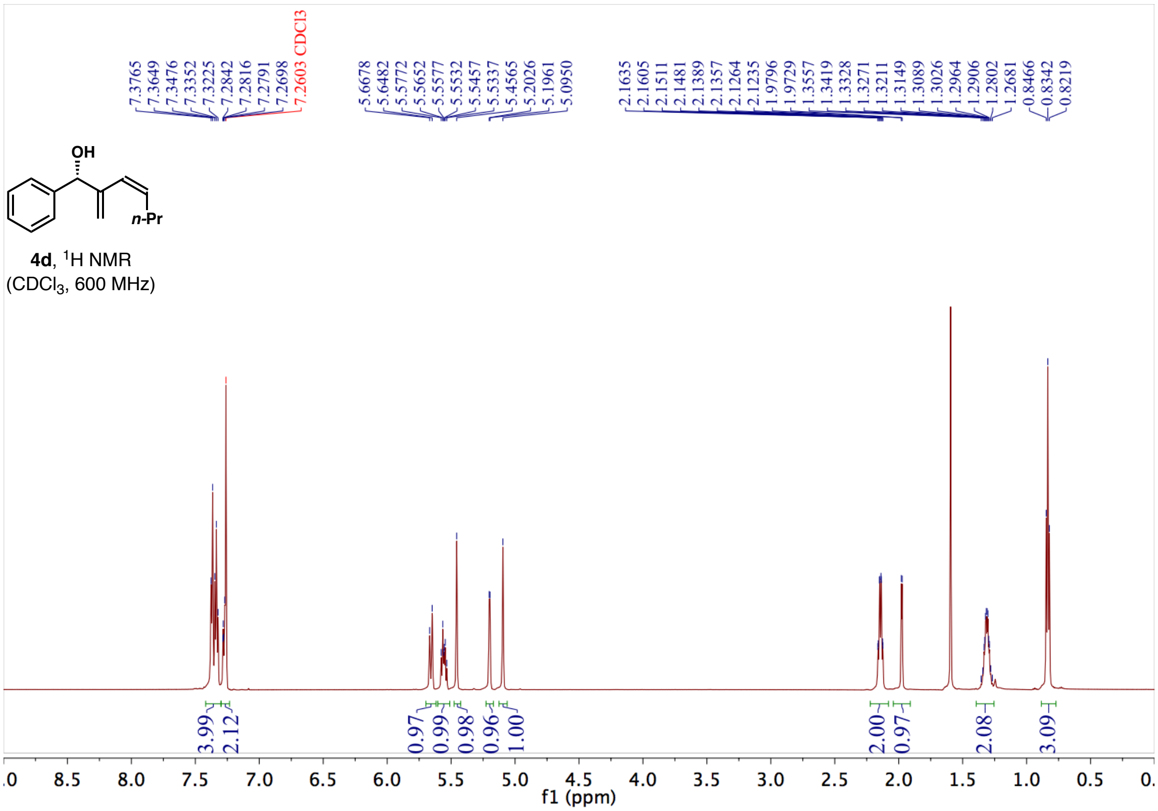
OH
n-Pr
4d, ^{1}H NMR
(CDCl$_3$, 600 MHz)
7.2603 CDCl3
3.99
2.12
0.97
0.99
0.98
0.96
1.00
2.00
0.97
2.08
3.09
f1 (ppm)

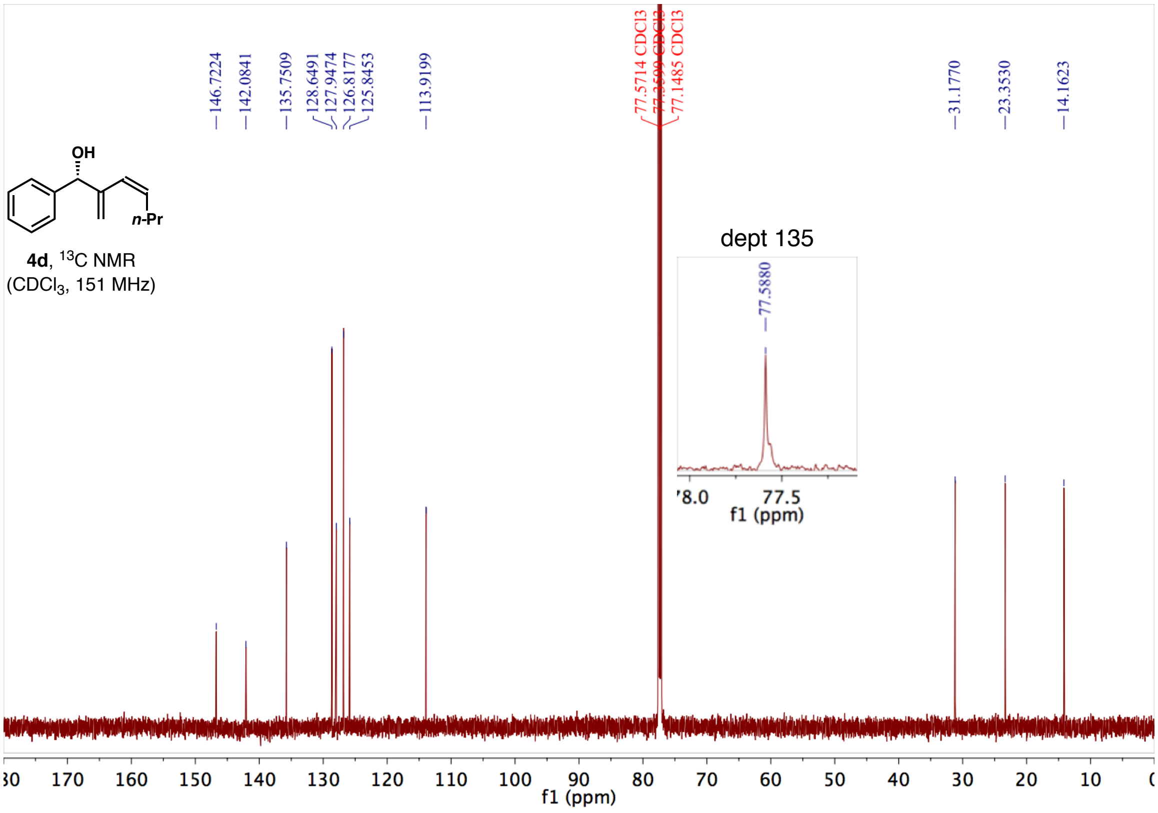
OH
n-Pr
4d, ^{13}C NMR
(CDCl$_3$, 151 MHz)
146.7224
142.0841
135.7509
128.6491
127.9474
126.8177
125.8453
113.9199
77.5714 CDCl3
77.1485 CDCl3
31.1770
23.3530
14.1623
dept 135
77.5880
f1 (ppm)

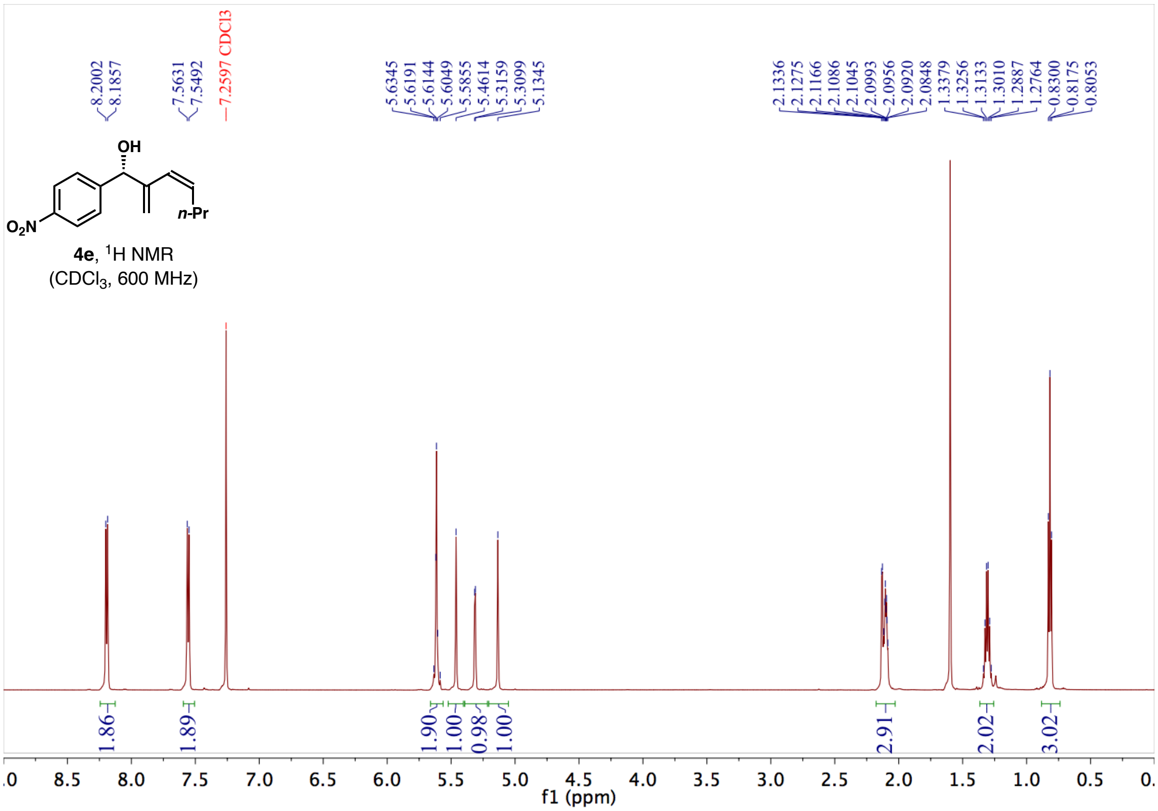
8.2002
8.1857
7.5631
7.5492
7.2597 CDCl3
5.6345
5.6191
5.6144
5.6049
5.5855
5.4614
5.3159
5.3099
5.1345
2.1336
2.1275
2.1166
2.1086
2.1045
2.0993
2.0956
2.0920
2.0848
1.3379
1.3256
1.3133
1.3010
1.2887
1.2764
0.8300
0.8175
0.8053
OH
O_2N
n-Pr
4e, ^{1}H NMR
($CDCl_3$, 600 MHz)
1.86
1.89
1.90
1.00
0.98
1.00
2.91
2.02
3.02
f1 (ppm)

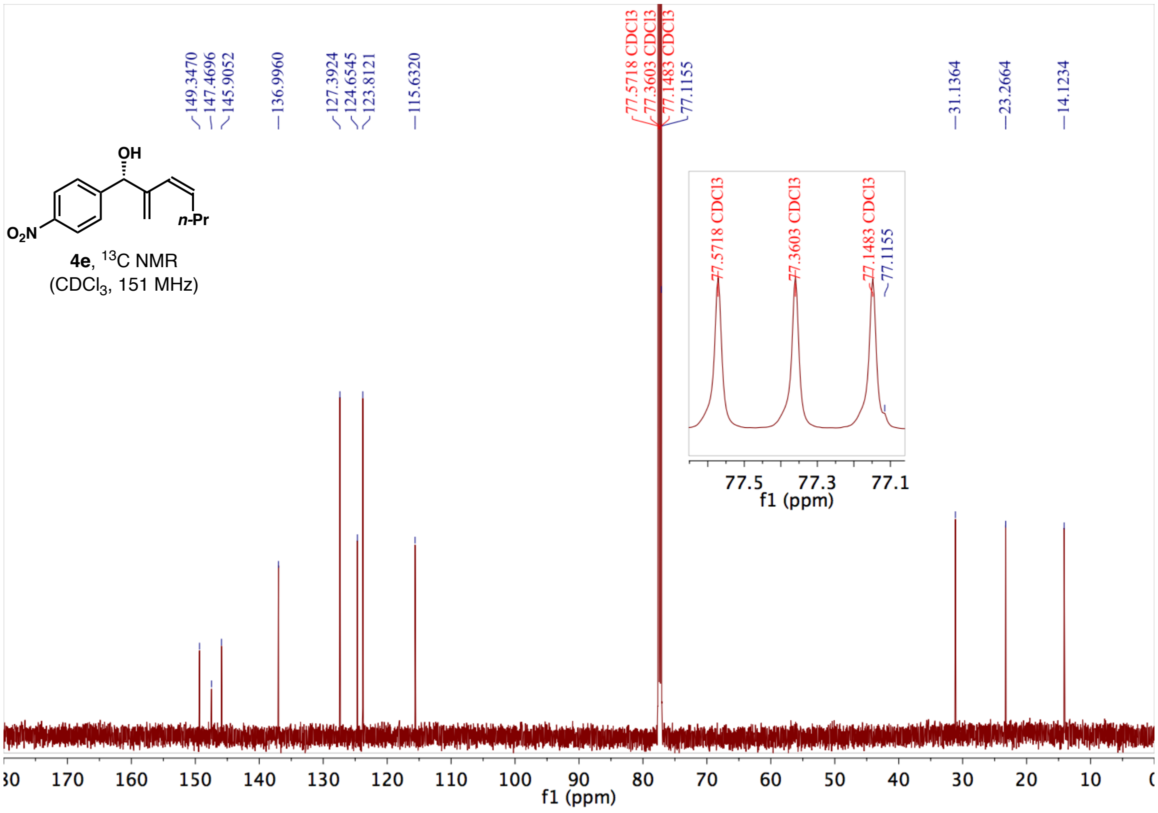
149.3470
147.4696
145.9052
136.9960
127.3924
124.6545
123.8121
115.6320
77.5718 CDCl3
77.3603 CDCl3
77.1483 CDCl3
77.1155
31.1364
23.2664
14.1234
OH
O_2N
n-Pr
4e, ^{13}C NMR
($CDCl_3$, 151 MHz)
f1 (ppm)

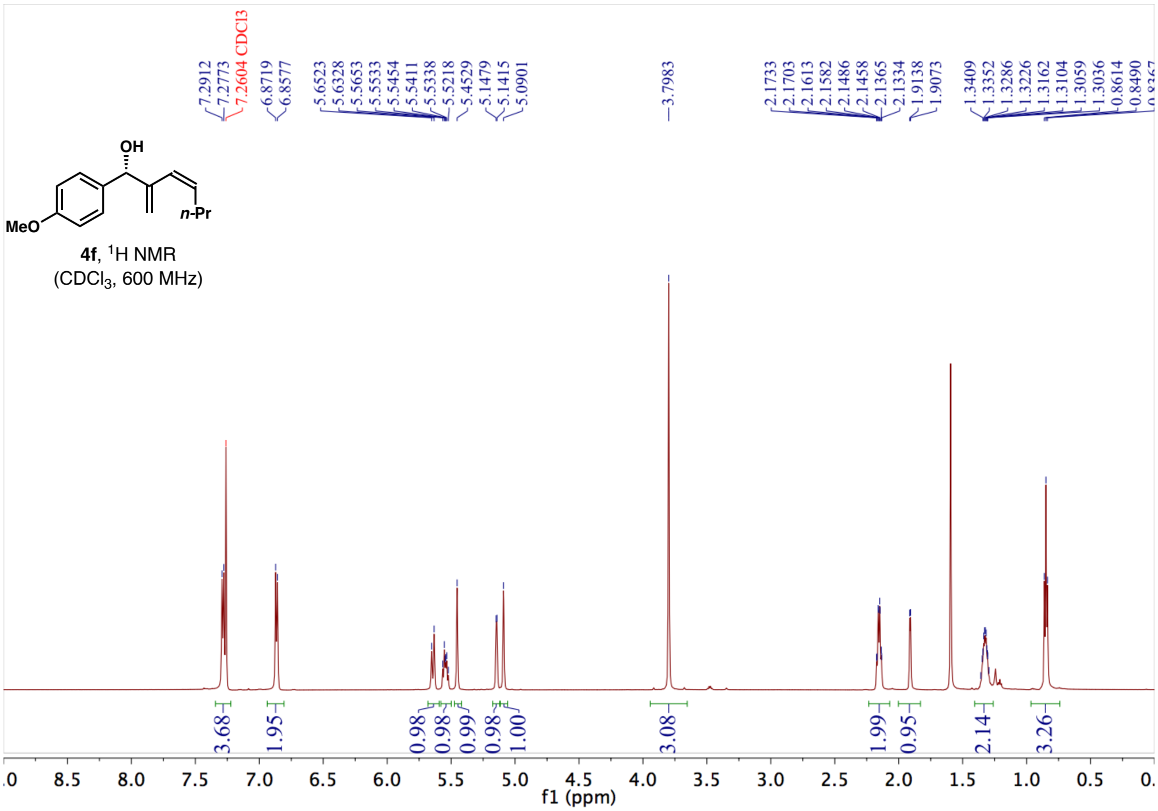
4f, 1H NMR
(CDCl3, 600 MHz)
OH
MeO
n-Pr
f1 (ppm)

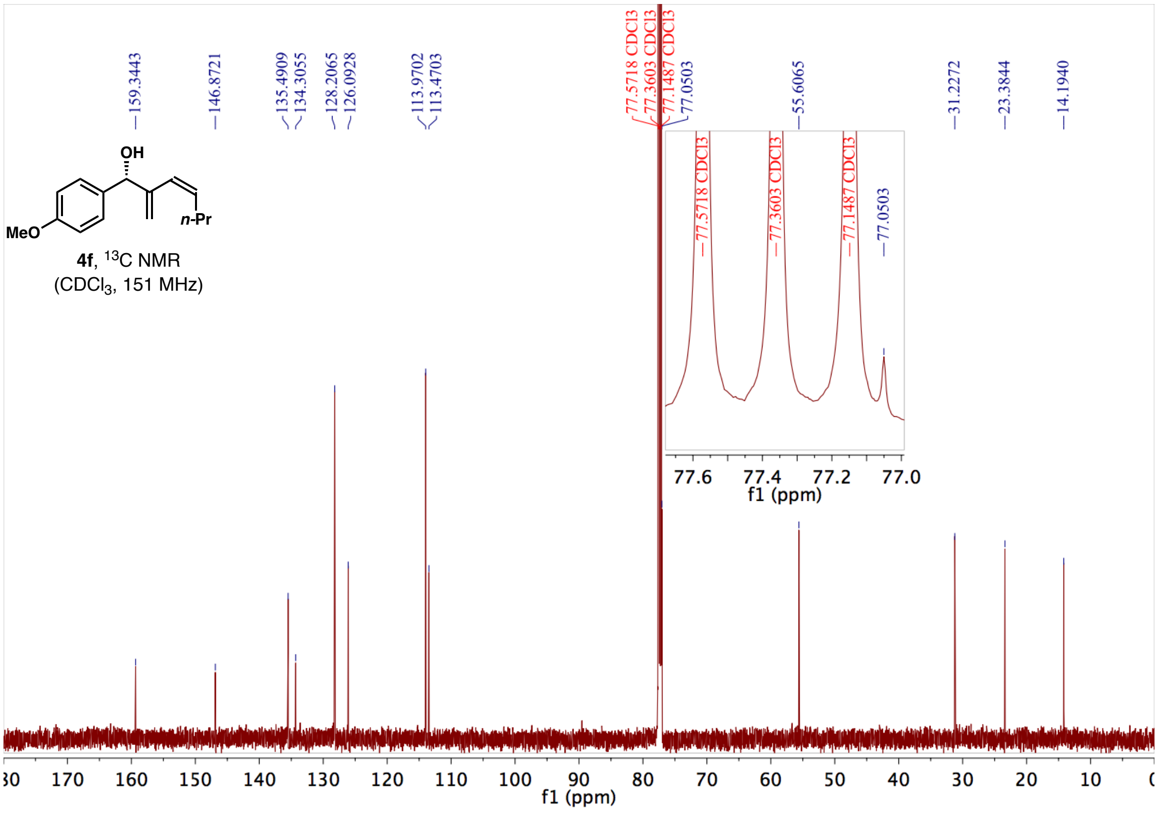
4f, 13C NMR
(CDCl3, 151 MHz)
OH
MeO
n-Pr
f1 (ppm)

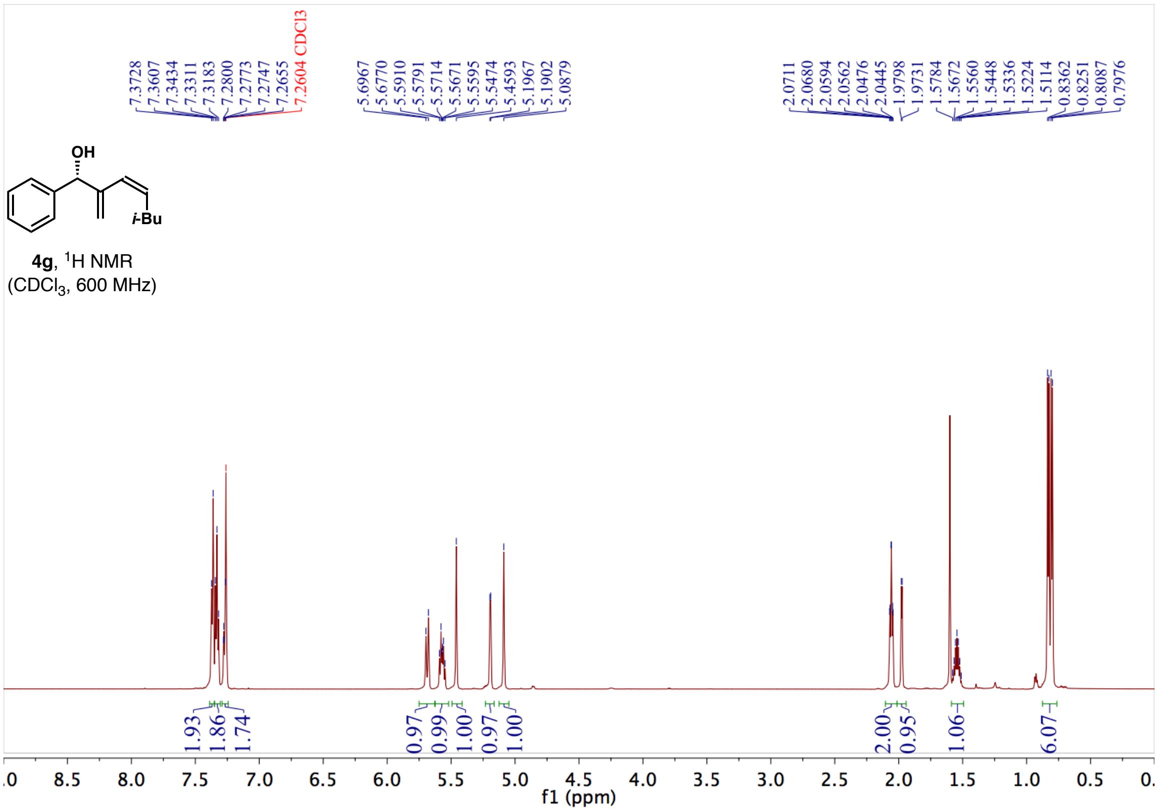

OH
i-Bu
4g, ¹H NMR
(CDCl3, 600 MHz)
7.3728
7.3607
7.3434
7.3311
7.3183
7.2800
7.2773
7.2747
7.2655
7.2604 CDCl3
5.6967
5.6770
5.5910
5.5791
5.5714
5.5671
5.5595
5.5474
5.4593
5.1967
5.1902
5.0879
2.0711
2.0680
2.0594
2.0562
2.0476
2.0445
1.9798
1.9731
1.5784
1.5672
1.5560
1.5448
1.5336
1.5224
1.5114
0.8362
0.8251
0.8087
0.7976
1.93
1.86
1.74
0.97
0.99
1.00
0.97
1.00
2.00
0.95
1.06
6.07
f1 (ppm)

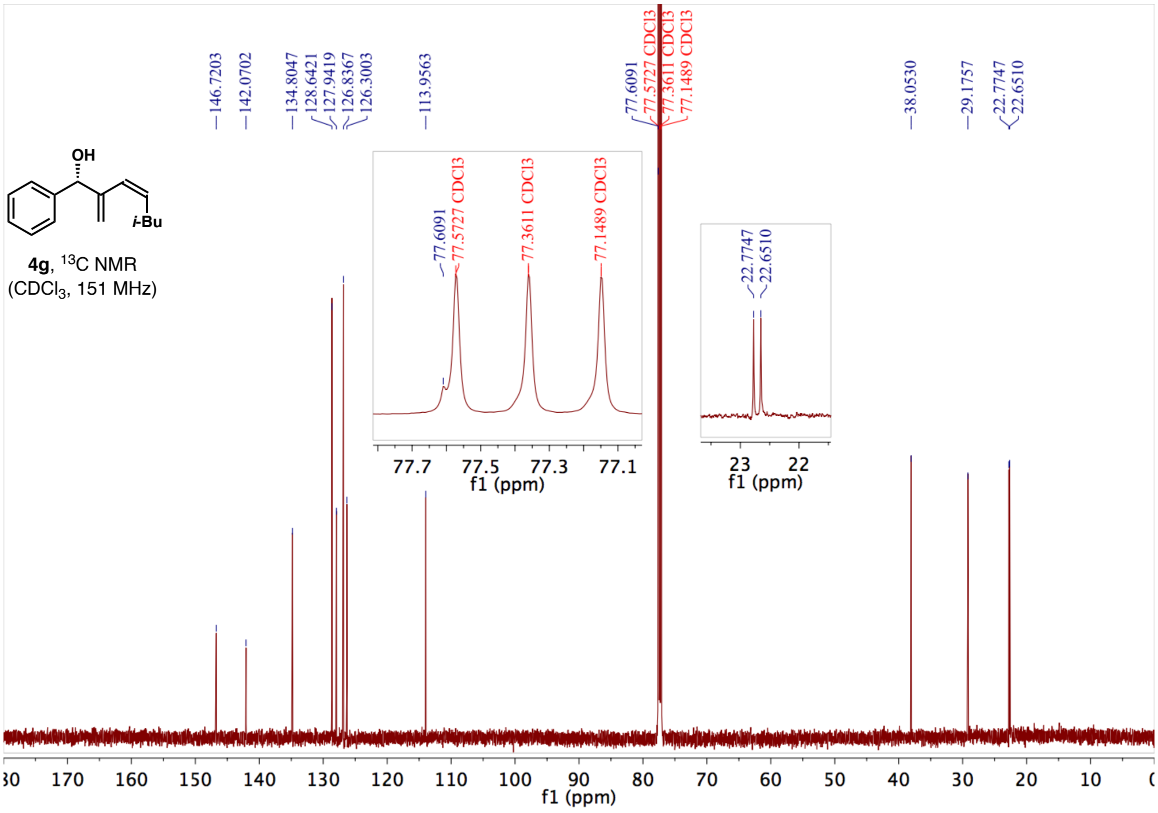

OH
i-Bu
4g, ¹³C NMR
(CDCl3, 151 MHz)
146.7203
142.0702
134.8047
128.6421
127.9419
126.8367
126.3003
113.9563
77.6091
77.5727 CDCl3
77.3611 CDCl3
77.1489 CDCl3
38.0530
29.1757
22.7747
22.6510
f1 (ppm)

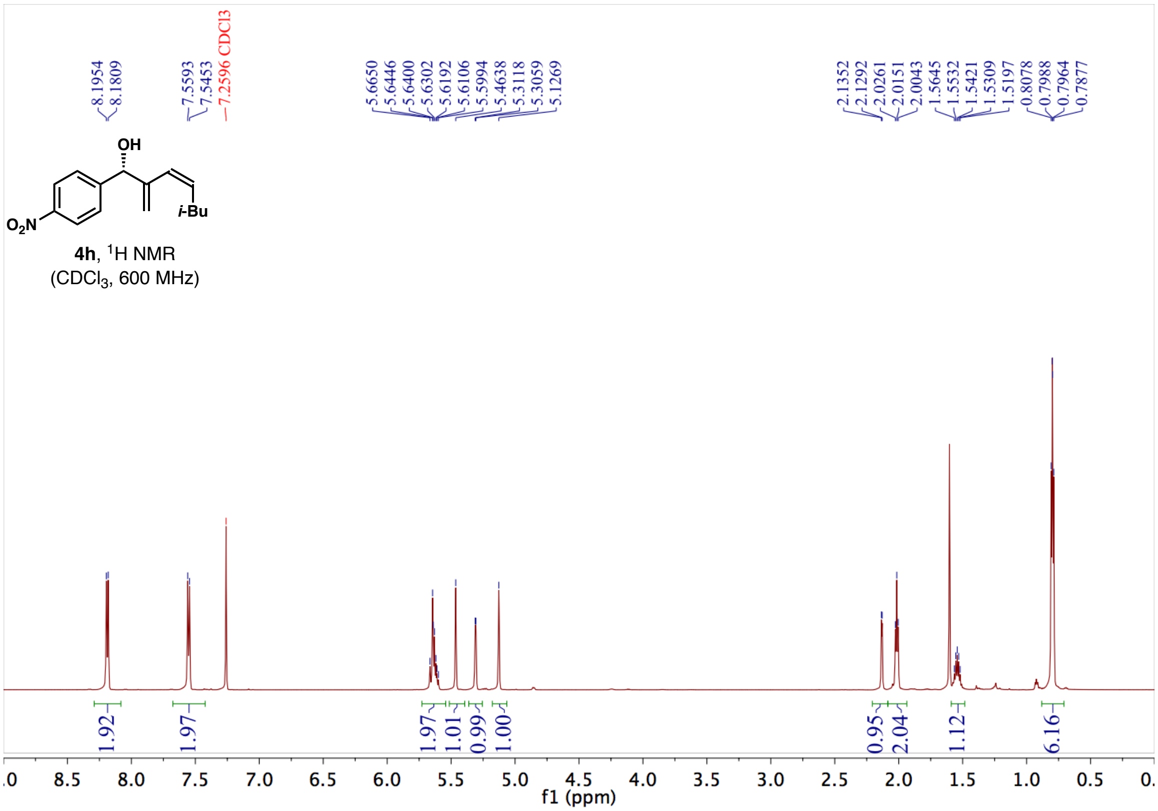
8.1954
8.1809
7.5593
7.5453
7.2596 CDCl3
5.6650
5.6446
5.6400
5.6302
5.6192
5.6106
5.5994
5.4638
5.3118
5.3059
5.1269
2.1352
2.1292
2.0261
2.0151
2.0043
1.5645
1.5532
1.5421
1.5309
1.5197
0.8078
0.7988
0.7964
0.7877
OH
O2N
i-Bu
4h, 1H NMR
(CDCl3, 600 MHz)
1.92
1.97
1.97
1.01
0.99
1.00
0.95
2.04
1.12
6.16
8.5
8.0
7.5
7.0
6.5
6.0
5.5
5.0
4.5
4.0
3.5
3.0
2.5
2.0
1.5
1.0
0.5
f1 (ppm)

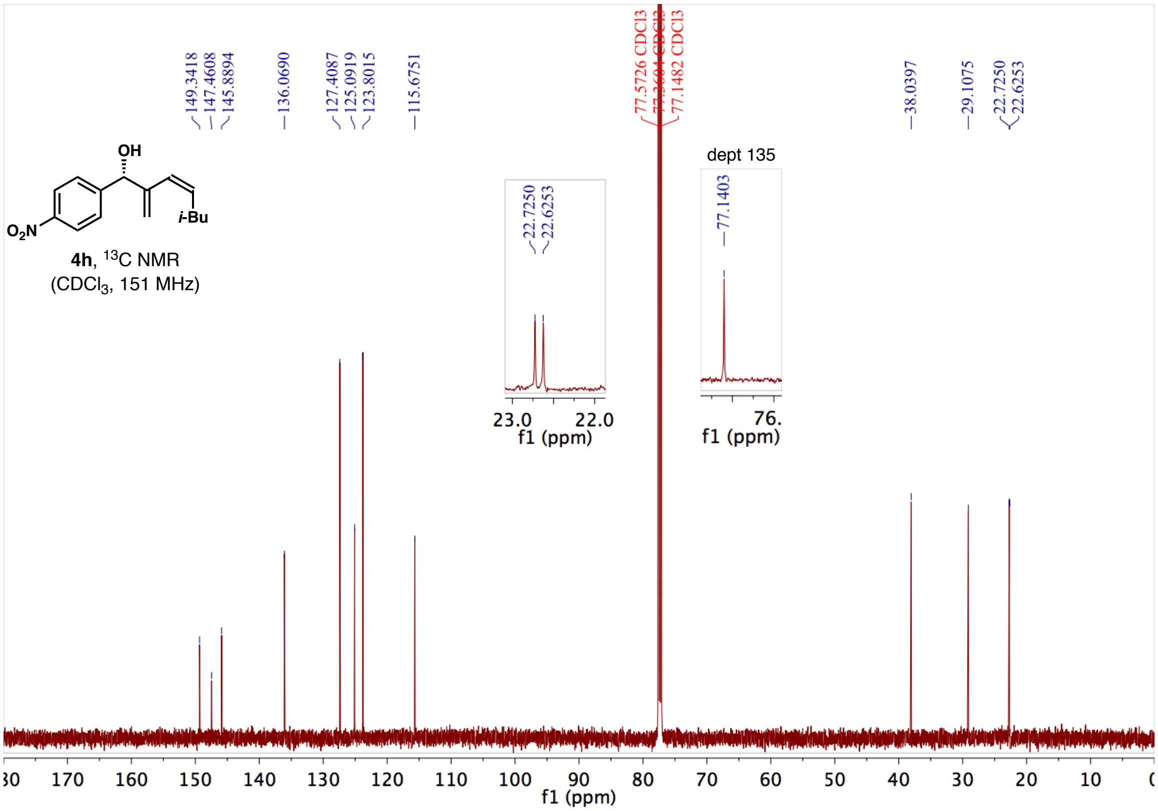
149.3418
147.4608
145.8894
136.0690
127.4087
125.0919
123.8015
115.6751
77.5726 CDCl3
77.1482 CDCl3
38.0397
29.1075
22.7250
22.6253
OH
O2N
i-Bu
4h, 13C NMR
(CDCl3, 151 MHz)
22.7250
22.6253
23.0
22.0
f1 (ppm)
dept 135
77.1403
76.
f1 (ppm)
170
160
150
140
130
120
110
100
90
80
70
60
50
40
30
20
10
f1 (ppm)

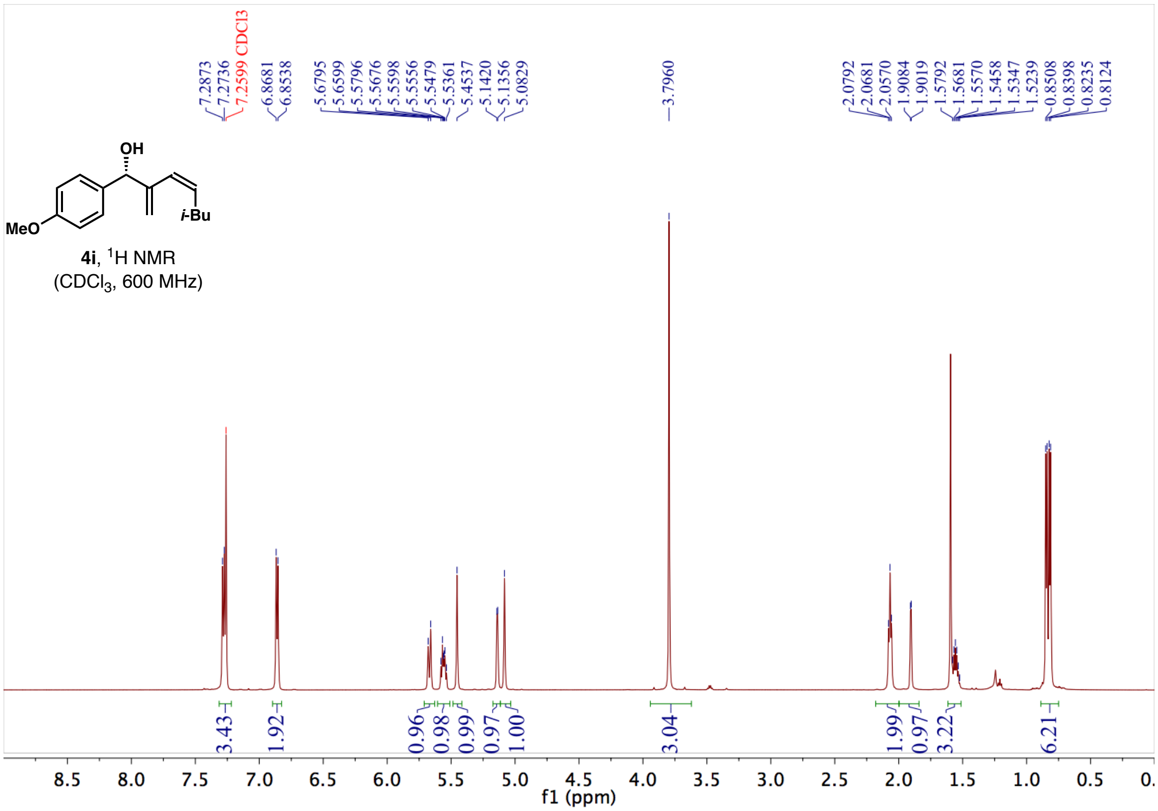
4i, ¹H NMR
(CDCl3, 600 MHz)
MeO
OH
i-Bu
f1 (ppm)

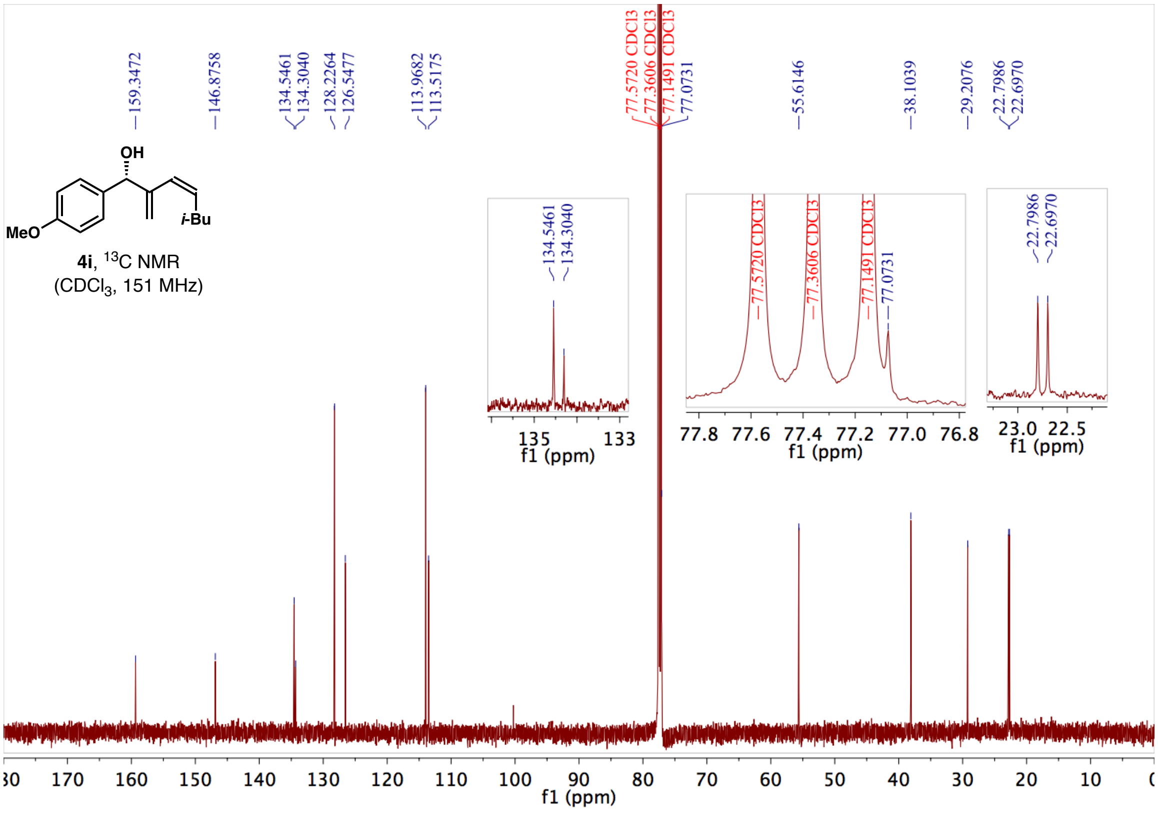
4i, ¹³C NMR
(CDCl3, 151 MHz)
MeO
OH
i-Bu
f1 (ppm)
